# Optimizing Veteran-Facing Materials for Vending Machine-Dispensed HIV Self-Testing: Formative Qualitative Study

**DOI:** 10.2196/93868

**Published:** 2026-07-23

**Authors:** Tessa Rife-Pennington, Michael P Douglas, Wendy Xie, Jennifer Cocohoba

**Affiliations:** 1 Pharmacy Service San Francisco VA Health Care System San Francisco, CA United States; 2 School of Pharmacy University of California, San Francisco San Francisco, CA United States; 3 Thomas J Long School of Pharmacy University of the Pacific San Francisco, CA United States

**Keywords:** veterans, HIV self-testing, vending machines, formative research, user-centered design, participatory action research, thematic analysis, study materials

## Abstract

**Background:**

Veterans face stigma, privacy concerns, and access barriers to HIV screening. For studies that use at-home HIV self-testing (HIVST) kits distributed through vending machines (VMs), recruitment and educational materials must communicate study purpose and participation options clearly, minimize confusion and stigma, and provide actionable next steps for participants who test outside of clinical settings.

**Objective:**

This study aimed to elicit structured feedback from a small group of veteran advocates living with HIV on recruitment flyers and education, survey, and interview materials for a Veterans Health Administration pilot evaluation of VM-dispensed HIVST kits and to document how this feedback informed prelaunch revisions.

**Methods:**

Using participatory action research, we recruited veteran advocates with lived and living expertise with HIV (August 2025). Veteran advocates completed structured written reviews of study materials and returned written feedback forms; feedback was also discussed during a 1-hour virtual focus group in September 2025. We analyzed written feedback and the focus group transcript using a rapid, team-based consensus thematic approach. Two study team members independently reviewed each feedback source and documented key recommendations and candidate feedback domains using analytic notes; the team then met to cluster feedback into domains and reach consensus on final domain labels and definitions. To ensure findings directly informed material improvement, we created a revision matrix mapping each feedback domain to the relevant study material or materials, a summary of feedback, and the resulting changes made. This matrix served as an audit trail linking feedback to the “feedback and revisions” tables presented in the Results section.

**Results:**

Four veteran advocates provided structured written feedback on study materials, and 3 participated in the 1-hour focus group. Across study materials, veteran advocates desired (1) clearer, plain-language descriptions of study purpose, eligibility, and participation pathways; (2) reduced potential for confusion between research recruitment, VM access, and HIVST kit promotion; (3) reduced text density and participant burden; and (4) more actionable “next steps,” including human support and linkage-to-care resources appropriate for at-home self-testing. Revisions included a streamlined recruitment flyer with simplified calls to action and clearer survey versus interview pathways; a more cohesive and condensed education packet oriented around self-testing steps, results interpretation, and support resources; questionnaire updates to reduce redundancy and improve usability; and an interview guide with improved flow, more participant-centered framing, and optional questions on emotional reactions and support needs.

**Conclusions:**

In this small formative review, veteran advocate feedback was systematically mapped to prelaunch revisions across multiple study materials. Transparently documenting stakeholder input and material adaptations may support future refinement of veteran-facing HIV screening and self-testing materials in Veterans Affairs and similar settings.

## Introduction

HIV screening is a critical component of prevention and early diagnosis, yet many US veterans experience barriers to routine testing [1]. Prior qualitative work in the Veterans Health Administration (VHA) suggests that concerns about stigma, confidentiality, and access can influence whether veterans seek HIV testing or follow-up care [1]. At-home HIV self-testing (HIVST) offers a complementary strategy that can expand options for screening outside traditional clinic encounters and may be particularly acceptable for individuals who prefer privacy and convenience [2]. However, HIVST remains a relatively novel approach among veterans and in VHA settings, and research studies evaluating HIVST programs must communicate clearly what participation involves and what to do after testing. Published evaluations of HIVST in veteran populations and within VHA settings remain limited, with much of the HIVST evidence base focused on nonveteran populations and distribution models outside federal health systems [3,4]. In addition, while vending machine (VM) distribution of HIVST kits has been examined outside the United States in nonveteran populations and community-based settings, there is minimal published evidence on VM HIVST kit delivery for US veterans [5,6].

Veterans are an important population for low-barrier HIV screening because HIV prevention and testing needs may intersect with mental health conditions, substance use, housing instability, rural residence, transportation barriers, and concerns about confidentiality [1]. Although the VHA provides integrated HIV prevention and treatment services, prior work suggests that routine HIV testing can still be affected by patient-, clinician-, and system-level barriers [7,8]. For veterans who prefer privacy or are not reached during routine clinical encounters, VM-based HIVST may offer a complementary access point within Veterans Affairs (VA) and VA-partnered settings [9].

Given the relative novelty of HIVST, and particularly VM-based HIVST kit distribution in VHA settings, formative input from veteran advocates living with HIV can strengthen implementation readiness by ensuring that participant-facing materials are understandable, acceptable, and actionable [10,11]. Structured veteran feedback can identify unclear terminology, confusing participation pathways, overly dense content, or language that inadvertently increases stigma, and it can guide revisions that reduce burden and improve usability. Despite increasing interest in low-barrier HIVST kit distribution models, including VM distribution, few reports describe how veterans are engaged to refine recruitment, educational, and data collection methods before a program launch, limiting reproducibility and the ability to translate these approaches into other VHA settings.

The objective of this formative study was to elicit structured feedback from a small group of veteran advocates living with HIV on recruitment, educational, survey, and interview materials for a VHA pilot evaluation of VM-dispensed HIVST kits and to transparently document how this feedback informed prelaunch revisions to these study materials.

## Methods

### Study Design and Setting

This was a formative qualitative study using a participatory action research [12-14] approach to refine veteran-facing recruitment and study materials prior to the launch of a pilot evaluation of VM-dispensed HIVST kits [10,11,15]. This approach was grounded in the premise that individuals with lived experience can identify potential barriers, unclear language, and acceptability concerns that may not be apparent to study teams before implementation [12-14]. Consistent with the formative purpose of the study, the analysis prioritized actionable feedback for material refinement rather than theory generation or broad characterization of veteran perspectives [10,11,15]. Reporting was guided by relevant qualitative reporting principles, and a completed SRQR (Standards for Reporting Qualitative Research) checklist is provided as Multimedia Appendix 1 [16]. The parent study is a RE-AIM (reach, effectiveness, adoption, implementation, and maintenance)-guided preimplementation and implementation evaluation of oral-fluid HIVST kits distributed through preexisting VMs in VA health care and supportive housing settings [9].

This work was conducted at the San Francisco VA Health Care System, which serves more than 310,000 US veterans at the San Francisco VA Medical Center and 9 community-based outpatient clinics located in downtown San Francisco, Oakland, San Bruno, Santa Rosa, Clearlake, Ukiah, and Eureka, California. In the parent program, HIVST kits are stocked in 15 VMs located at the San Francisco VA Medical Center, community-based outpatient clinics (except San Bruno), and 6 partnered supportive housing buildings. Veteran advocate review activities were completed remotely (asynchronous written feedback and a virtual focus group), enabling participation across the San Francisco Bay Area.

### Participants and Recruitment

We used participatory action research methodology to recruit 4 veteran advocates with HIV who resided in California [12-14]. Because the goal was targeted prelaunch materials refinement rather than thematic saturation or representative sampling, the study used a small purposive sample of veteran advocates to identify actionable feedback before implementation. Veterans were recruited through 2 nurse practitioners with expertise in HIV prevention and treatment who were embedded in outpatient primary care and infectious diseases clinics. Referred veterans were contacted by phone to solicit interest and participation (August 2025).

Veteran advocates living with HIV were intentionally recruited because the study team sought feedback from veterans with experience navigating HIV-related stigma, testing, disclosure, linkage to care, and VA-based HIV services. Their perspectives were considered particularly relevant for identifying language, design elements, and follow-up instructions that could reduce stigma and support veterans who may test alone outside a clinical encounter. However, because all reviewers were living with HIV, their baseline HIV knowledge and prior testing experiences may differ from those of veterans who have never tested, test infrequently, or have limited HIV knowledge.

We did not collect individual sociodemographic characteristics because this was a small, prelaunch formative materials review rather than a study designed to characterize veteran perspectives. Collecting detailed demographic information could have increased identifiability among a very small group of veteran advocates recruited through local HIV care networks. No minimum descriptive categories were reported because, with only 4 participants, even broad combinations of characteristics could risk deductive disclosure.

### Study Materials for Review

Research study materials for review by veteran advocates included recruitment flyers (Multimedia Appendix 2), education handouts (Multimedia Appendix 3), an electronic web-based questionnaire (Multimedia Appendix 4), and a semistructured qualitative interview guide (Multimedia Appendix 5). Table 1 describes the initial design plan and features of the study materials, which were developed collaboratively by the study principal investigator (PI) and program manager.

**Table 1:** Research study materials for veteran advocate feedback.

Study materials	Initial design plan and features
Recruitment flyers	Designed to be colorful, engaging, and include either a graphic image of an HIVST^a^ or representative images of diverse populations; publicly available images sourced [17] Included 7 different options
Education handouts	Planned to be packaged with each individual HIVST kit Included 3 standard handouts to be packaged with all HIVST kits: the Centers for Disease Control and Prevention HIV 101 fact sheet; the US Department of Veterans Affairs Rapid Oral HIV fact sheet; and the TakeMeHome fact sheet Included 5 handout options by California county (Alameda, Humboldt, Lake and Mendocino, San Francisco, and Sonoma); 1 handout was packaged with the HIVST kits based on VM^b^ location and respective county List and pin map of community-based resources for HIV testing and treatment
Electronic web-based questionnaire	Available for completion by veterans who obtained an HIVST kit dispensed via a VM Designed to evaluate the following: participant demographics and characteristics, sexual partners and practices, and drug use practices; a use plan for the HIVST kit, results, and steps after testing; and perspectives and experiences accessing and using the VM-dispensed HIVST kit Included a direct link and QR code to the questionnaire for electronic completion
Semistructured qualitative interview guide	Available for completion by veterans who obtained and used an HIVST kit dispensed via a VM Designed to assess perspectives and experiences accessing and using a VM-dispensed HIVST

^a^HIVST: HIV self-testing.

^b^VM: vending machine.

### Procedures

In September 2025, veterans were mailed electronic and paper copies of a feedback instructions sheet (Multimedia Appendix 6), a feedback documentation form (Multimedia Appendix 7), and research study materials. Veterans were instructed to mail (paper or electronic) copies of the feedback form in advance of the focus group with a 2-week turnaround time.

The research study team independently reviewed and collaboratively discussed feedback forms received prior to the focus group, which was held virtually via Zoom (Zoom Communications) for 1 hour, and was audio recorded and transcribed (September 2025). The focus group was moderated by the study PI using a standardized script, and an assistant moderator provided technical support, monitored the chat, and took notes [9]. The moderator and assistant moderator debriefed immediately after the focus group [18].

### Feedback Prompts

The feedback documentation form and focus group guide included structured, open-ended prompts tailored to each study material. For the recruitment flyers, veterans were asked whether the flyer answered key questions about the study (eg, what the study is about, what participation involves, why it matters, eligibility, and how to get involved), and to comment on visual relevance and representation (including veterans and diverse populations), attention-grabbing design, readability and technical language, and ease of acting on the information (including the usefulness of the QR code and website and low-tech alternatives), along with concrete suggestions to improve engagement.

For the education handouts, prompts asked for overall impressions, relevance, clarity (including confusing sections), appropriateness of the educational level, and recommendations to remove or add materials and make the handouts more engaging. For the web-based questionnaire, veterans were asked about their overall experience and first impressions, relevance, clarity and flow, perceived intrusiveness (including whether questions about HIV self-test results felt intrusive), completion time, device used, technical issues, ease of completion, and suggestions to improve engagement and user-friendliness. For the qualitative interview guide, prompts focused on overall impressions, relevance, clarity and flow, intrusiveness, recommended deletions or additions, wording changes, and ways to make the interview more engaging. During the focus group, the moderator followed the same topical structure and used standard probes (eg, asking participants to elaborate or provide examples) to clarify and deepen feedback.

### Researcher Characteristics and Reflexivity

The study PI is a clinical pharmacist practitioner and harm reduction coordinator involved in the parent HIVST VM program and moderated the focus group. Because the study team developed the materials under review, we used structured feedback forms, a standardized focus group guide, team debriefing, and an audit trail–based revision matrix to support reflexivity and reduce the risk that revisions reflected only study team assumptions.

### Qualitative Analysis

We analyzed written veteran advocate feedback documents and the transcript of the audio-recorded focus group using a rapid, team-based consensus thematic approach designed for formative refinement of study materials [10,11,19,20]. Given the small number of veteran advocates and feedback sources, we did not develop a formal codebook or conduct independent line-by-line coding. Instead, 2 study team members independently reviewed each feedback source in full and documented key recommendations and candidate feedback domains using analytic notes. The team then met to discuss areas of convergence and divergence, iteratively cluster feedback into domains, and reach consensus on final domain labels and their definitions [21,22].

To ensure findings directly informed materials improvement, we created a revision matrix that mapped each feedback domain to (1) the specific study material it applied to, (2) a summary of the feedback, and (3) the resulting change or changes made. This matrix served as an audit trail linking veteran feedback to specific adaptations and informed the “feedback and revisions” tables presented in the Results section [23]. We enhanced rigor through team debriefing, documentation of analytic decisions and revisions in the matrix, and triangulation across written and group feedback sources. We did not conduct participant checking because the purpose was rapid prelaunch materials refinement, and revisions were needed before implementation. To reduce the risk of deductive identification in this small sample, we present concise paraphrased examples of feedback rather than verbatim quotes. Accordingly, findings are reported as actionable feedback domains for material refinement rather than as fully saturated qualitative themes. Feedback domains and recommendations were translated into proposed edits by the study PI, then reviewed by the study team via electronic review and verbal discussion and iteratively revised by the PI until a consensus was reached.

### Data Storage

Collected data were stored in password-protected files on the San Francisco VA Health Care System network, and access was restricted to research study staff. Veteran advocates were assigned alpha-numeric identifiers, and files with identifying information will be destroyed upon study completion.

**Ethical Considerations**

We obtained approval from the University of California, San Francisco Institutional Review Board and the San Francisco Veterans Affairs Human Subjects Committee in August 2025 (25-43931). Veteran advocate participants provided verbal consent to participate and received compensation aligned with the time commitment for each activity (written material review and focus group participation) per protocols; 1 participant completed electronic material review only and received the written-only compensation amount.

## Results

Four veteran advocates completed the structured written review of the study materials. Three of the 4 also participated in the 1-hour virtual focus group; 1 completed the written review only [10]. Because the activity was designed to refine study materials before launch rather than to generate generalizable themes, results are presented as summarized, paraphrased feedback domains and linked to material revisions in Tables 2-5.

**Table 2:** Veteran advocate feedback and informed revisions to recruitment flyer.

Feedback theme	Summarized veteran advocate feedback	Veteran advocate–informed revisions made
Clarify purpose and “why it matters”	Clearer statement of purpose and benefit of the project beyond compensation	Updated the title or heading and purpose statement
Stronger call to action, clarify eligibility criteria	Clearer, simpler instructions on exactly how to participate Confusion regarding eligibility Need to clearly distinguish survey participation from optional interview participation	Clarified eligibility at the top Explicitly separated survey and interview participation pathways and described each option in plain language Added a simplified “Getting Started” action
Clarify participation pathways and time burden	Needed a clearer explanation of what “participation” means and how the survey differs from the optional interview	Added brief plain-language descriptions of the survey (confidential online survey) and interview (1-h interview)
Reduce text, highlight key details	Flyers were too wordy and technical; emphasize essentials (eligibility, what participation involves, and compensation)	Modernized the font, visuals, and layout Removed detailed step-by-step procedural content and condensed the flyer to essential information Reorganized content into short, labeled sections to improve readability
Pictures and imagery	Visuals should more clearly reflect veterans (including diverse representation) or include more graphic icons	Replaced images of people and HIV self-test with graphic icons Moved away from photo-heavy designs toward a simplified, text-forward layout with clear section headings
QR code and low-tech alternatives	Liked the QR code option; include phone number and website	Clarified that survey participation begins by scanning the QR code on the HIV self-test kit Provided direct study team contact information (phone and email) for the optional interview
Reduce confusion between recruitment and testing promotion	Unclear whether flyers were recruiting for the study or promoting HIV self-testing	Removed confusing language, unnecessary content, and streamlined sections and flow
Compensation clarity and visual emphasis	Compensation details should be easy to find, quickly understood, and emphasized visually (eg, dollar signs or pictures)	Moved compensation into a standalone “Payment” section and simplified wording (including the cap for the survey incentive) Visual emphasis elements were not added due to institutional review board requirements for recruitment materials

**Table 3:** Veteran advocate feedback and informed revisions to education handouts.

Feedback theme	Summarized veteran advocate feedback	Veteran advocate–informed revisions made
Clarify packet purpose and audience	Some content felt mismatched to at-home testing (eg, clinic-based rapid oral testing language) Unclear which handouts applied to veterans using the self-test versus general information Requested a more cohesive, self-test–specific packet	Streamlined the packet to target veterans who use a vending machine–dispensed HIV self-testing kit Oriented all materials around self-testing workflow (what HIV is, what results mean, and next steps and support)
Prioritize the core steps	Information overload (multiple handouts plus lengthy tables and maps of resources) Asked to emphasize essentials for someone testing alone How to perform the test, timing, and what to do after the results	Reduced the overall number and length of handouts Removed or de-emphasized nonessential materials (eg, TakeMeHome fact sheet and extensive resource tables or maps) Created a concise “how-to” sheet (including timing and a QR-linked video) plus a results interpretation and next-steps handout
Human support; crisis and mental health resources	Emphasized the need for a real person to contact, clear linkage to confirmatory testing, and crisis and mental health resources for veterans who may feel distressed after unexpected results when testing alone	Added explicit follow-up instructions and support pathways (contact VA^a^ primary care clinician or pharmacist for confirmatory testing and next steps) Included Veterans Crisis Line information
Visual design consistency	Liked the clarity and visual simplicity of the CDC^b^ HIV 101 fact sheet format Recommended carrying forward a consistent, cohesive look and simplifying the layout across the packet	Retained the CDC HIV 101 fact sheet Designed new self-test instructions and results pages to mirror a concise, step-based flow and consistent, plain-language presentation across the packet
Simplify resource navigation	Resource information was too long and dense (tables, maps, and long lists); asked for a shorter, easier-to-scan set of local options plus clear direction on where to start	Replaced extensive county tables with a brief, scannable list of free local resources by county Included a simple option to request mailed self-tests (TakeMeHome link) alongside VA follow-up instructions

^a^VA: Veterans Affairs.

^b^CDC: Centers for Disease Control and Prevention.

**Table 4:** Veteran advocate feedback and informed revisions to the questionnaire.

Feedback theme	Summarized veteran advocate feedback	Veteran advocate–informed revisions made
Streamline length and reduce redundancy	Some questionnaire items were repetitive and long Consolidate overlapping questions to reduce burden	Removed multiple items assessing perceived comfort, effort, and satisfaction with accessing HIVST^a^ kits via VM^b^ (while retaining core constructs such as confidence, acceptability, convenience, and privacy)
Improve usability (navigation and mobile use)	User experience improvements (eg, back button, clearer required-item behavior, and mobile-friendly)	Added back button and slide page transitions and clarified response formats for key items to reduce user confusion (eg, converting select questions to “select all that apply” where multiple responses are plausible)
Response option gaps and sensitivity safeguards	Recommended ensuring nonresponse options for sensitive items (eg, “prefer not to answer”) Asked about whether “0 tests” should be an option for the initial VM access question	Retained “prefer not to answer” throughout the instrument Did not add “0 tests” because eligibility required accessing ≥1 HIVST kit via the VM, and the access slider begins at 1
Formatting clarity	Format changes (bold key information), clarify order, and refine response formats (eg, slider and cost question)	Reformatted the introduction to improve readability (eg, organizing survey content areas as brief bullet points rather than a dense paragraph) and adjusted layout and spacing

^a^HIVST: HIV self-testing.

^b^VM: vending machine.

**Table 5:** Veteran advocate feedback and informed revisions to qualitative interview guide.

Feedback theme	Summarized veteran advocate feedback	Veteran advocate–informed revisions made
Reduce length and redundancy and improve flow	Guide was long and repetitive Consolidate overlapping sections and clarify flow	Reorganized the guide into labeled sections (background and prior testing, using the vending machine, using the HIV self-test, follow-up and linkage, values and preferences, and expansion) Condensed overlapping questions by using targeted probes within questions
Clarify and distinguish vending machine access from the HIV self-testing experience	Recommended more clearly distinguishing questions about (1) obtaining the HIV self-test from the vending machine and (2) using the HIV self-test (including the testing experience and result interpretation), to avoid conflating 2 different steps	Reorganized the interview guide into separate, clearly labeled sections (“Using the Vending Machine” and “Using the HIV Self-Test”) Revised prompts and probes to align questions with the appropriate step
Trauma-informed, participant-centered framing	Recommended a more reassuring introduction (no right or wrong answers) and careful wording to avoid participants feeling coerced or judged	Added a participant-centered introduction emphasizing voluntary participation and autonomy (no right or wrong answers; option to skip questions, take a break, or stop at any time) to reduce pressure and support comfort
Address emotional responses and supports after self-testing	Recommended acknowledging that self-testing may provoke worry or anxiety and ensuring that the guide assesses support needs and preferred supports	Added an optional question on emotional reactions to results (with “okay to skip”) Expanded support probes to include crisis and mental health resources and clear next steps

For the recruitment flyer, veterans emphasized the need to clarify the flyer’s purpose and why participation matters beyond compensation, strengthen the call to action, and simplify eligibility and participation instructions. They also recommended reducing technical language and overall text density, updating the layout, and using imagery that more clearly reflects veterans and diverse representation. Finally, veterans supported the use of a QR code while recommending low-tech alternatives and clearer contact options and noted the importance of avoiding confusion between research recruitment and promotion of HIVST. Revisions to address these points are summarized in Table 2. Compared with the initial flyers, the revised flyer (Multimedia Appendix 8) substantially reduced procedural detail and reorganized content into brief, labeled sections that clearly separated survey versus interview pathways, highlighted compensation in plain language, and provided a simplified “getting started” call to action. Veterans also recommended making the paid nature of the study more visually salient (eg, adding dollar signs and/or images to emphasize compensation); however, these visual elements could not be incorporated due to institutional review board–related requirements.

Veteran advocates recommended streamlining the education packet to better align with at-home HIVST (rather than testing with a health care provider) and prioritize clear, actionable next steps (how to use the test, how to interpret results, and where to seek support); in response, we reduced the number and length of handouts, replaced clinic-oriented rapid oral testing materials with a self-test instruction sheet and a results and support handout, and condensed the community-based resource information while adding clear VA follow-up and crisis support resources (Multimedia Appendix 9). Revisions, including reducing the number of handouts and improving visual cohesion, are summarized in Table 3.

Veteran advocates recommended reducing questionnaire length and redundancy to minimize burden and improving clarity and usability; in response, we removed multiple items assessing perceived comfort, effort, and satisfaction with accessing HIV self-tests via the VM and clarified several question stems and response formats (eg, allowing “select all that apply” for HIV self-test use plans, testing location, and next steps) while retaining sensitivity safeguards such as “prefer not to answer” options (Multimedia Appendix 10). Content and formatting updates are described in Table 4.

For the qualitative interview guide, veterans perceived the guide as long, repetitive, and recommended consolidating overlapping sections and improving flow with clearer transitions. Feedback also supported more participant-centered, trauma-informed framing (eg, a reassuring introduction and language that reduces the risk of participants feeling judged or coerced). Veterans recommended optional questions about emotional reactions and support needs, and revisions also emphasized participant autonomy (eg, the ability to skip questions, take a break, or stop). Compared with the original interview guide, the revised guide (Multimedia Appendix 11) was reorganized into clearly labeled sections, streamlined to reduce redundancy, and strengthened with participant-centered, trauma-informed language (eg, reminders that participants may skip questions, take breaks, or stop), with added probes addressing privacy and surveillance concerns, emotional reactions to results, and support needs (Table 5).

Separate from veteran advocate feedback, the study team incorporated team-driven refinements identified during internal review to strengthen implementation-relevant domains, including more specific probes addressing privacy and surveillance concerns and barriers and facilitators to confirmatory testing and follow-up care. On the basis of early interview scheduling contacts, the study team also added questions to capture peer-to-peer distribution and support (eg, whether the HIV self-test was obtained for oneself or someone else, experiences helping another person use the test, and what resources would make peer support feasible and safe).

Across study materials, feedback consistently prioritized clarity, reduced burden, modernized graphics and formatting, and actionable next steps and resulted in concrete revisions to improve the comprehensibility and usability of veteran-facing materials.

## Discussion

### Principal Findings

This formative study provides a transparent example of prelaunch materials refinement for a VA-based HIVST evaluation. Structured input from a small purposive sample of veteran advocates living with HIV helped identify potential points of confusion and opportunities to improve participant-facing materials. Given the limited sample size, findings should be interpreted as targeted formative feedback on specific study materials rather than evidence of broader veterans’ preferences or the effectiveness of the revised materials. Within this scope, the feedback helped the study team revise recruitment, education, survey, and interview materials to improve clarity, reduce burden, and better communicate participation steps and follow-up options.

In addition to veteran advocate–informed revisions, the study team incorporated refinements identified through internal review and early operational learnings (eg, probes addressing privacy and confidentiality considerations, confirmatory testing and follow-up barriers and facilitators, and emerging peer-support scenarios). We describe these as team-driven updates to maintain clear attribution and transparency regarding the source of revisions. This distinction is important because formative evaluation often involves iterative cycles that integrate stakeholder feedback with implementation team learning as programs move toward launch [10].

These findings align with the formative evaluation and acceptability methods literature demonstrating that early, stakeholder-engaged approaches can improve the clarity, acceptability, and usability of participant-facing materials and study procedures prior to implementation [10,18]. Prior formative and qualitative studies of VM delivery models for HIVST similarly suggest that uptake and acceptability are shaped by end-user experience at the point of contact, including whether materials and processes are understandable and easy to navigate [24]. Participatory design work focused on HIVST communication tools also underscores the value of iteratively refining instructions and support for results interpretation to reduce user error and improve understanding [25].

For VA and similar health system settings, this work suggests that participant-facing materials should be treated as implementation components rather than administrative add-ons. In a low-barrier HIVST model, materials may shape whether veterans understand the purpose of the program, know how to use the test, feel comfortable completing study activities, and know where to seek confirmatory testing or support. Simple, low-cost adjustments, such as shorter text, clearer headings, fewer redundant questions, and materials organized around the real sequence of actions veterans take, may reduce friction during recruitment, self-testing, and follow-up.

More broadly, this formative activity contributes to efforts to expand low-barrier, patient-centered HIV prevention and screening options, particularly for populations who may experience stigma or access constraints in traditional clinical pathways. As VA and other health systems explore novel distribution models, including VM-based access points, this formative work highlights the importance of evaluating not only the physical availability of the tests but also how clearly programs communicate purpose, steps, and follow-up options.

This study has limitations. First, the formative sample was small, consisting of 4 veteran advocates, 3 of whom participated in the focus group and 1 of whom completed the written review only. The purpose of the activity was to refine study materials before launch, not to generate representative or generalizable findings about all veterans who may use VM-dispensed HIVST kits. Findings should therefore be interpreted as targeted formative feedback from a small advisory group.

Second, all veteran advocates were living with HIV. This was intentional because the study team sought input from veterans with lived experience navigating HIV-related stigma, testing, linkage to care, and VA HIV services. However, veterans living with HIV may have greater baseline HIV knowledge, more familiarity with testing and treatment systems, and different perceptions of HIVST materials than veterans who have never tested, test infrequently, or have limited HIV knowledge. Future materials testing should include veterans not living with HIV, including those with varying levels of HIV knowledge and prior testing experience.

Third, we did not collect sociodemographic characteristics. This decision was made to reduce identifiability in a very small group recruited through local HIV care networks. However, the absence of demographic information limits our ability to assess transferability across age, gender identity, race and ethnicity, sexual orientation, rurality, housing status, digital access, HIV testing history, and prior experience with VA care. Future work should include larger and more diverse samples that allow the reporting of participant characteristics while maintaining confidentiality.

Fourth, data were collected through a structured written review and one virtual focus group. This approach was practical for prelaunch materials refinement but provided limited opportunity for repeated rounds of feedback, usability testing, or formal assessment of comprehension. Fifth, our rapid, team-based consensus thematic approach did not include a formal codebook or independent line-by-line coding. This approach was appropriate for a small formative dataset focused on actionable revisions, but it may reduce reproducibility compared with more intensive qualitative analytic methods. We mitigated this limitation by maintaining an audit trail–based revision matrix linking domains to material changes. Finally, this study documents material refinement prior to launch and does not evaluate whether the revised materials improved downstream outcomes, including understanding or comprehension, recruitment yield, survey or interview completion, self-testing behavior, or linkage to confirmatory testing or care. These outcomes will be assessed in the parent implementation evaluation.

### Conclusions

In this formative prelaunch study, feedback from a small group of veteran advocates living with HIV was systematically mapped to revisions in recruitment, educational, survey, and interview materials for a VA VM-based HIVST study. Veteran advocates prioritized plain-language messaging, reduced burden, clearer separation between research participation and self-testing, actionable next steps, and support framing for veterans testing outside clinical encounters. Although findings are not intended to represent all veterans or demonstrate implementation impact, this work provides a transparent, adaptable example of how stakeholder feedback can be systematically mapped to veteran-facing HIVST material revisions before implementation.

## Data Availability

Deidentified individual participant data will not be made publicly available because the written feedback forms and focus group transcript contains potentially identifying information, and complete deidentification cannot be assured given the small sample and local recruitment context.

## Appendix

Multimedia Appendix 1 SRQR (Standards for Reporting Qualitative Research) checklist.

Multimedia Appendix 2 Deidentified research study recruitment flyer prior to the incorporation of veteran advocate feedback.

Multimedia Appendix 3 Deidentified research study educational materials prior to the incorporation of veteran advocate feedback.

Multimedia Appendix 4 Deidentified research study electronic questionnaire prior to the incorporation of veteran advocate feedback.

Multimedia Appendix 5 Deidentified research study qualitative interview guide prior to the incorporation of veteran advocate feedback.

Multimedia Appendix 6 Deidentified research study feedback instruction sheet for veteran advocates.

Multimedia Appendix 7 Feedback form for veteran advocates.

Multimedia Appendix 8 Deidentified research study recruitment flyer after the incorporation of veteran advocate feedback.

Multimedia Appendix 9 Deidentified research study educational materials after the incorporation of veteran advocate feedback.

Multimedia Appendix 10 Deidentified research study electronic questionnaire after the incorporation of veteran advocate feedback.

Multimedia Appendix 11 Deidentified research study qualitative interview guide after the incorporation of veteran advocate feedback.

## Abbreviations

HIVST: HIV self-testing
PI: principal investigator
RE-AIM: reach, effectiveness, adoption, implementation, and maintenance
SRQR: Standards for Reporting Qualitative Research
VA: Veterans Affairs
VHA: Veterans Health Administration
VM: vending machine
