# Supplementary material for: Optimizing Veteran-Facing Materials for Vending Machine-Dispensed HIV Self-Testing: Formative Qualitative Study
Source: JMIR Form Res. 2026 Jul 23;10:e93868. doi: 10.2196/93868 (PMC13448198; doi:10.2196/93868)
Supplement: Supplementary file 1 [file formative_v10i1e93868_app2.pdf]

University of California San Francisco

U.S. Department of Veterans Affairs

Veterans Health Administration
San Francisco VA Health Care System

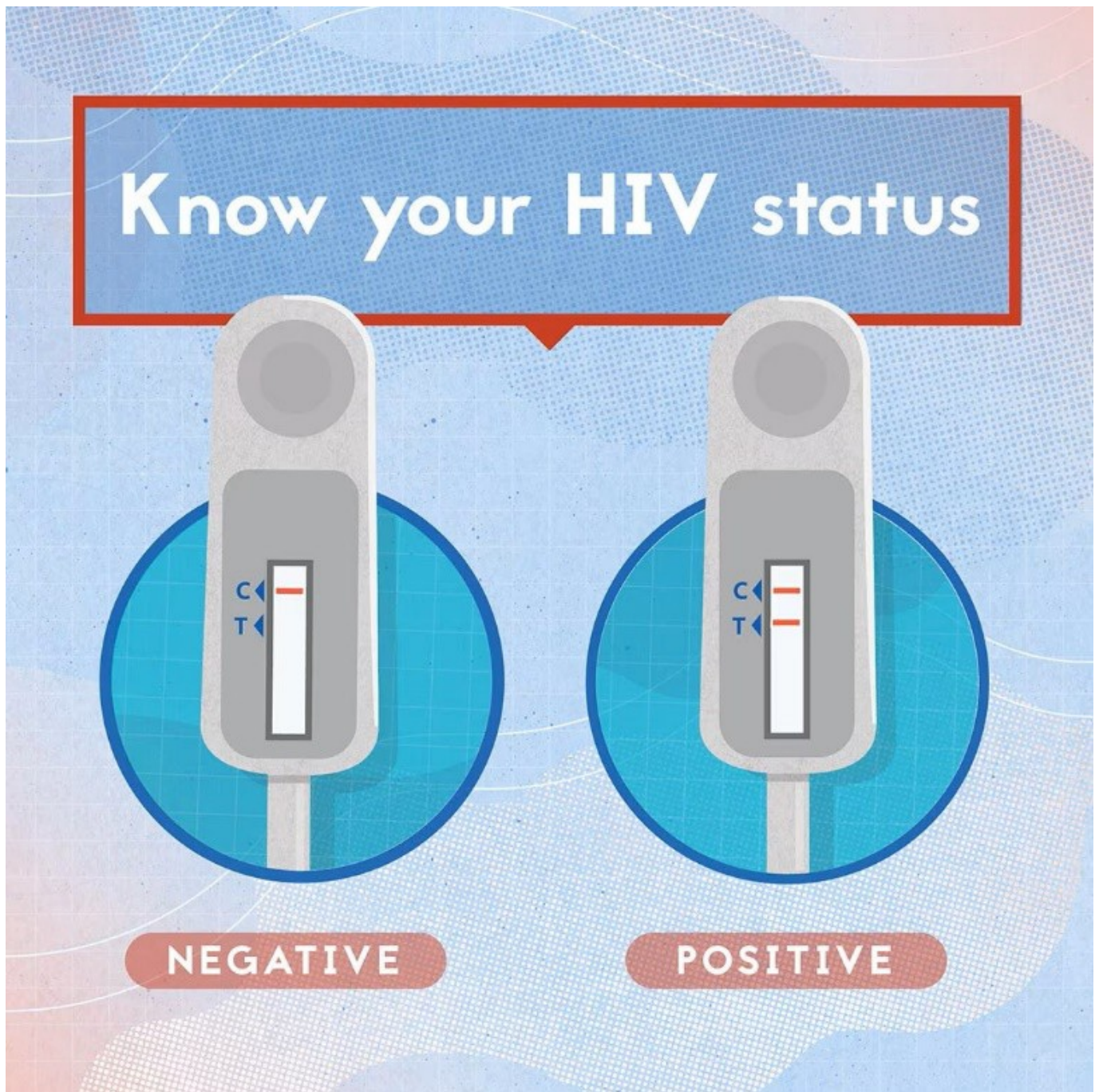

UCSF and the San Francisco VA Health Care System seek Veteran volunteers to participate in a paid research study.

Our goal is to learn about Veteran interest and experience using vending machines to access human immunodeficiency virus (HIV) self-tests.

Study participants will:

For Survey

- ✓ Use their assigned card or pin number to access to the VA Harm Reduction Vending Machine
- ✓ Obtain an HIV self-test from the VA Harm Reduction Vending Machine
- ✓ Look for the paid research study form attached to the HIV self-test
- ✓ Scan the QR code or go to the website provided on the form to take a confidential online survey (participants who forgo payment will be anonymized).
- ✓ (Optional): Provide your name and email at the end of the survey to receive a $20 electric gift card. There is no limit to the number of times the survey can be taken, but participants will only be paid for 2 surveys or up to $40.

For Interview

- ✓ Participants who use the HIV self-test may also refer themselves to take part in a one-time 1-hour interview.
- ✓ Interview participants will be paid a $100 gift card

Need access to the VA Harm Reduction Vending Machine? Have questions or concerns? Contact pharmacist [study PI, phone, email].

Interested in learning about our research team and projects? Scan this QR code or visit our web site at [insert website].

University of California San Francisco

U.S. Department of Veterans Affairs
Veterans Health Administration
San Francisco VA Health Care System

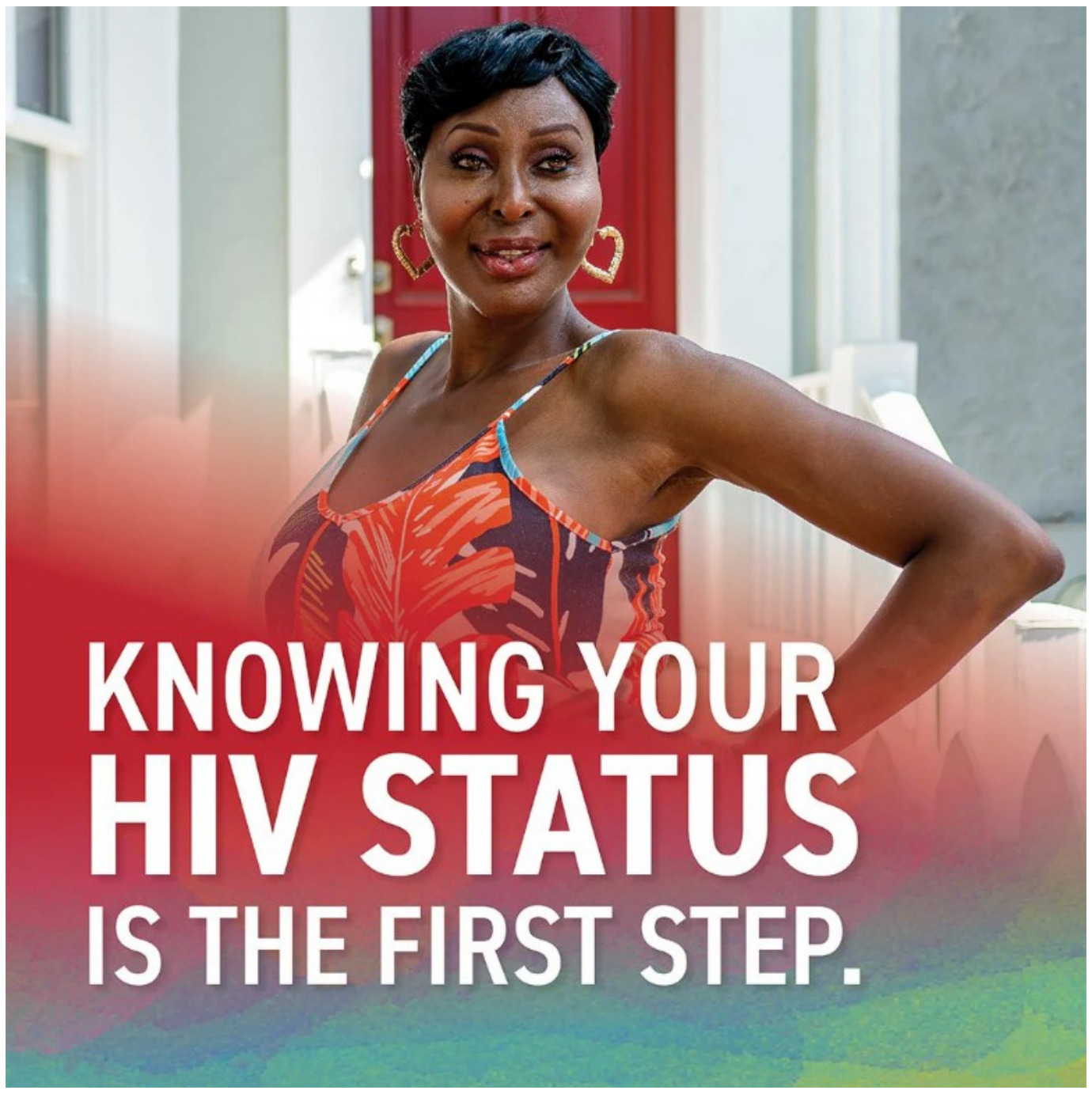

UCSF and the San Francisco VA Health Care System seek Veteran volunteers to participate in a paid research study.

Our goal is to learn about Veteran interest and experience using vending machines to access human immunodeficiency virus (HIV) self-tests.

Study participants will:

For Survey

- ✓ Use their assigned card or pin number to access to the VA Harm Reduction Vending Machine
- ✓ Obtain an HIV self-test from the VA Harm Reduction Vending Machine
- ✓ Look for the paid research study form attached to the HIV self-test
- ✓ Scan the QR code or go to the website provided on the form to take a confidential online survey (participants who forgo payment will be anonymized).
- ✓ (Optional): Provide your name and email at the end of the survey to receive a $20 electric gift card. There is no limit to the number of times the survey can be taken, but participants will only be paid for 2 surveys or up to $40.

For Interview

- ✓ Participants who use the HIV self-test may also refer themselves to take part in a one-time 1-hour interview.
- ✓ Interview participants will be paid a $100 gift card

Need access to the VA Harm Reduction Vending Machine? Have questions or concerns? Contact pharmacist [study PI, phone, email].

Interested in learning about our research team and projects? Scan this QR code or visit our web site at [insert website].

UCSF University of California San Francisco

VA | U.S. Department of Veterans Affairs

Veterans Health Administration
San Francisco VA Health Care System

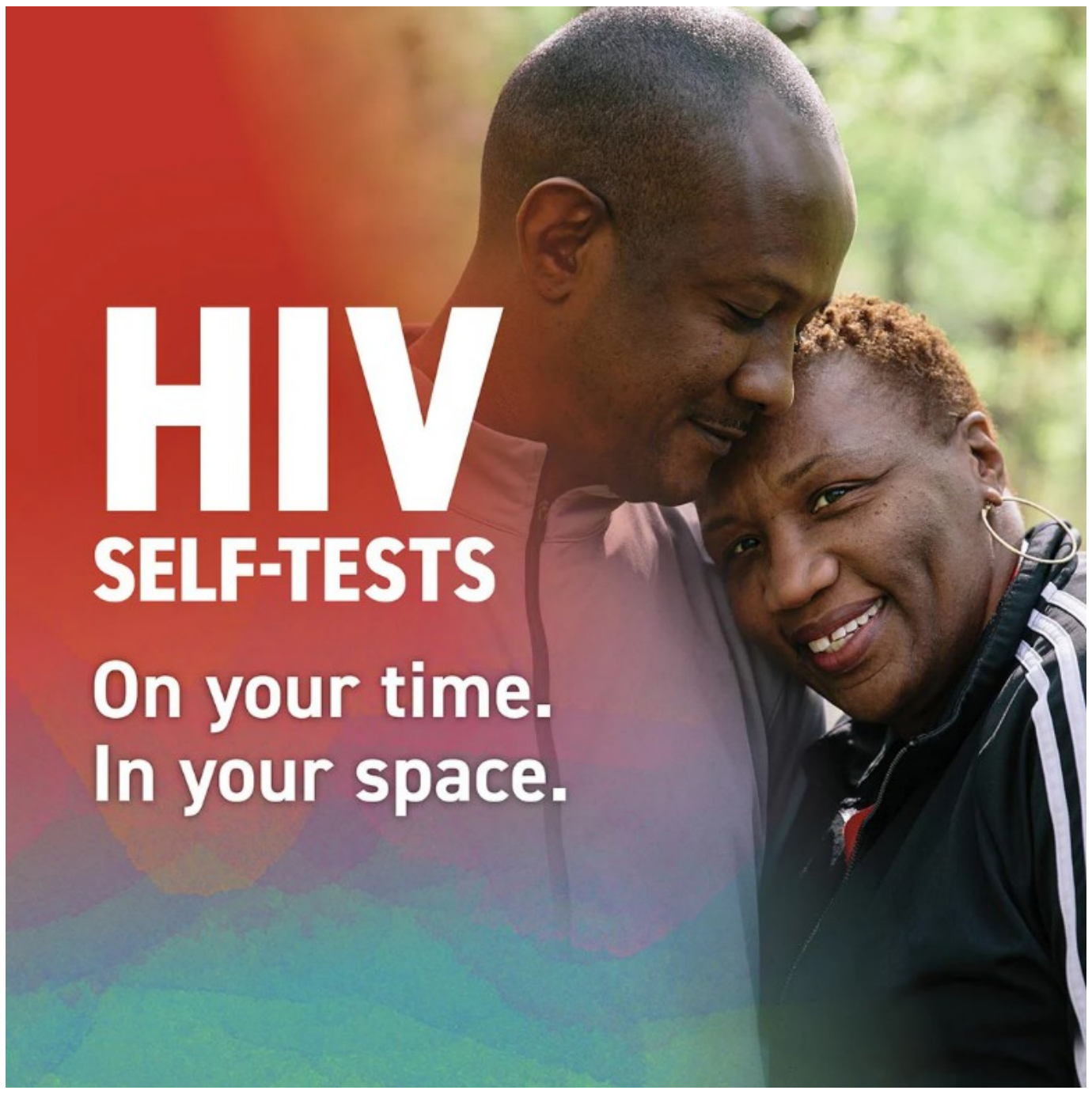

UCSF and the San Francisco VA Health Care System seek Veteran volunteers to participate in a paid research study.

Our goal is to learn about Veteran interest and experience using vending machines to access human immunodeficiency virus (HIV) self-tests.

Study participants will:

For Survey

- ✓ Use their assigned card or pin number to access to the VA Harm Reduction Vending Machine
- ✓ Obtain an HIV self-test from the VA Harm Reduction Vending Machine
- ✓ Look for the paid research study form attached to the HIV self-test
- ✓ Scan the QR code or go to the website provided on the form to take a confidential online survey (participants who forgo payment will be anonymized).
- ✓ (Optional): Provide your name and email at the end of the survey to receive a $20 electric gift card. There is no limit to the number of times the survey can be taken, but participants will only be paid for 2 surveys or up to $40.

For Interview

- ✓ Participants who use the HIV self-test may also refer themselves to take part in a one-time 1-hour interview.
- ✓ Interview participants will be paid a $100 gift card

Need access to the VA Harm Reduction Vending Machine? Have questions or concerns? Contact pharmacist [study PI, phone, email].

Interested in learning about our research team and projects? Scan this QR code or visit our web site at [insert website].

UCSF University of California San Francisco

VA | U.S. Department of Veterans Affairs
Veterans Health Administration
San Francisco VA Health Care System

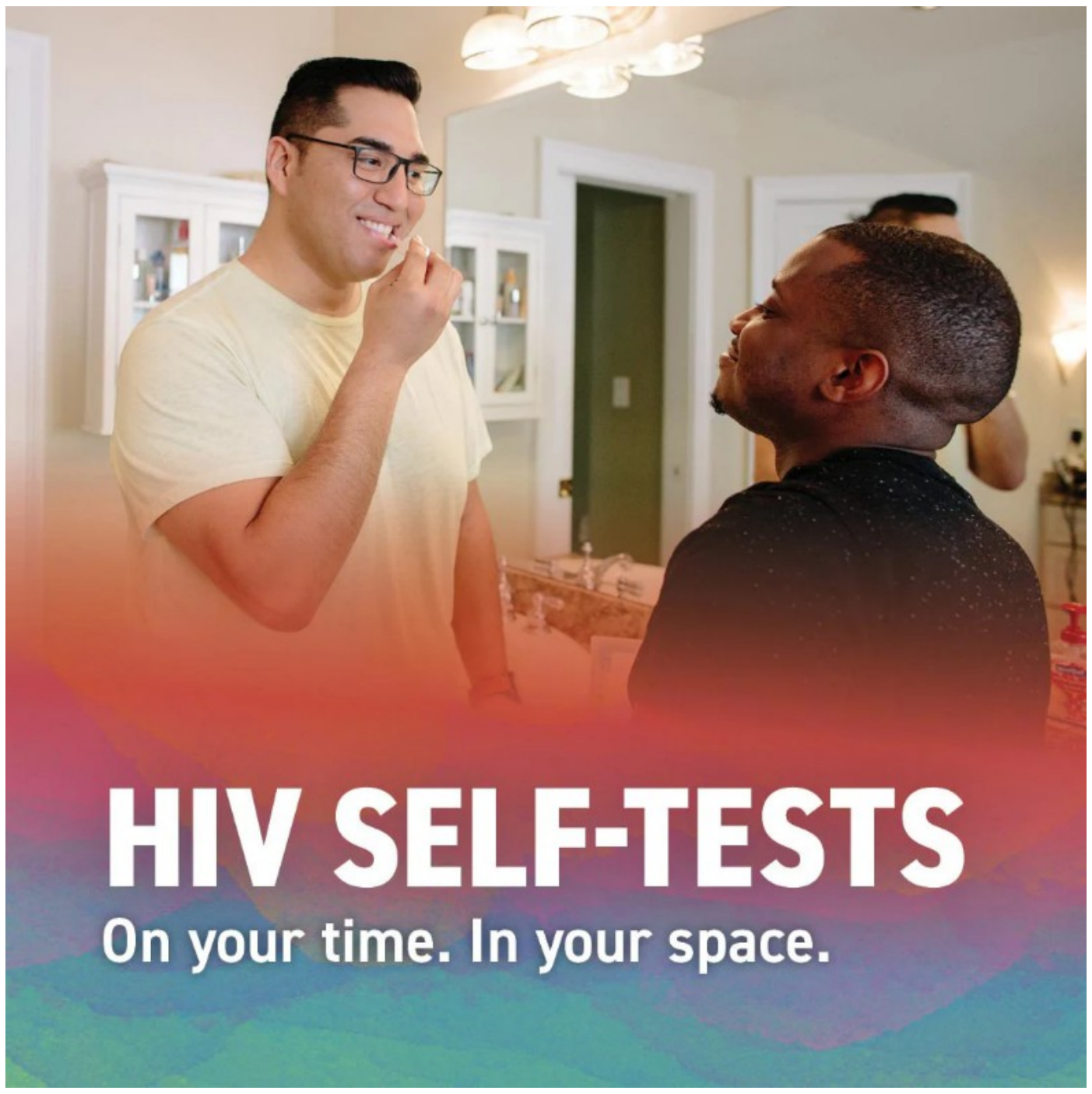

UCSF and the San Francisco VA Health Care System seek Veteran volunteers to participate in a paid research study.

Our goal is to learn about Veteran interest and experience using vending machines to access human immunodeficiency virus (HIV) self-tests.

Study participants will:

For Survey

- ✓ Use their assigned card or pin number to access to the VA Harm Reduction Vending Machine
- ✓ Obtain an HIV self-test from the VA Harm Reduction Vending Machine
- ✓ Look for the paid research study form attached to the HIV self-test
- ✓ Scan the QR code or go to the website provided on the form to take a confidential online survey (participants who forgo payment will be anonymized).
- ✓ (Optional): Provide your name and email at the end of the survey to receive a $20 electric gift card. There is no limit to the number of times the survey can be taken, but participants will only be paid for 2 surveys or up to $40.

For Interview

- ✓ Participants who use the HIV self-test may also refer themselves to take part in a one-time 1-hour interview.
- ✓ Interview participants will be paid a $100 gift card

Need access to the VA Harm Reduction Vending Machine? Have questions or concerns? Contact pharmacist [study PI, phone, email].

Interested in learning about our research team and projects? Scan this QR code or visit our web site at [insert website].

UCSF
University of California
San Francisco

VA

U.S. Department of Veterans Affairs
Veterans Health Administration
San Francisco VA Health Care System

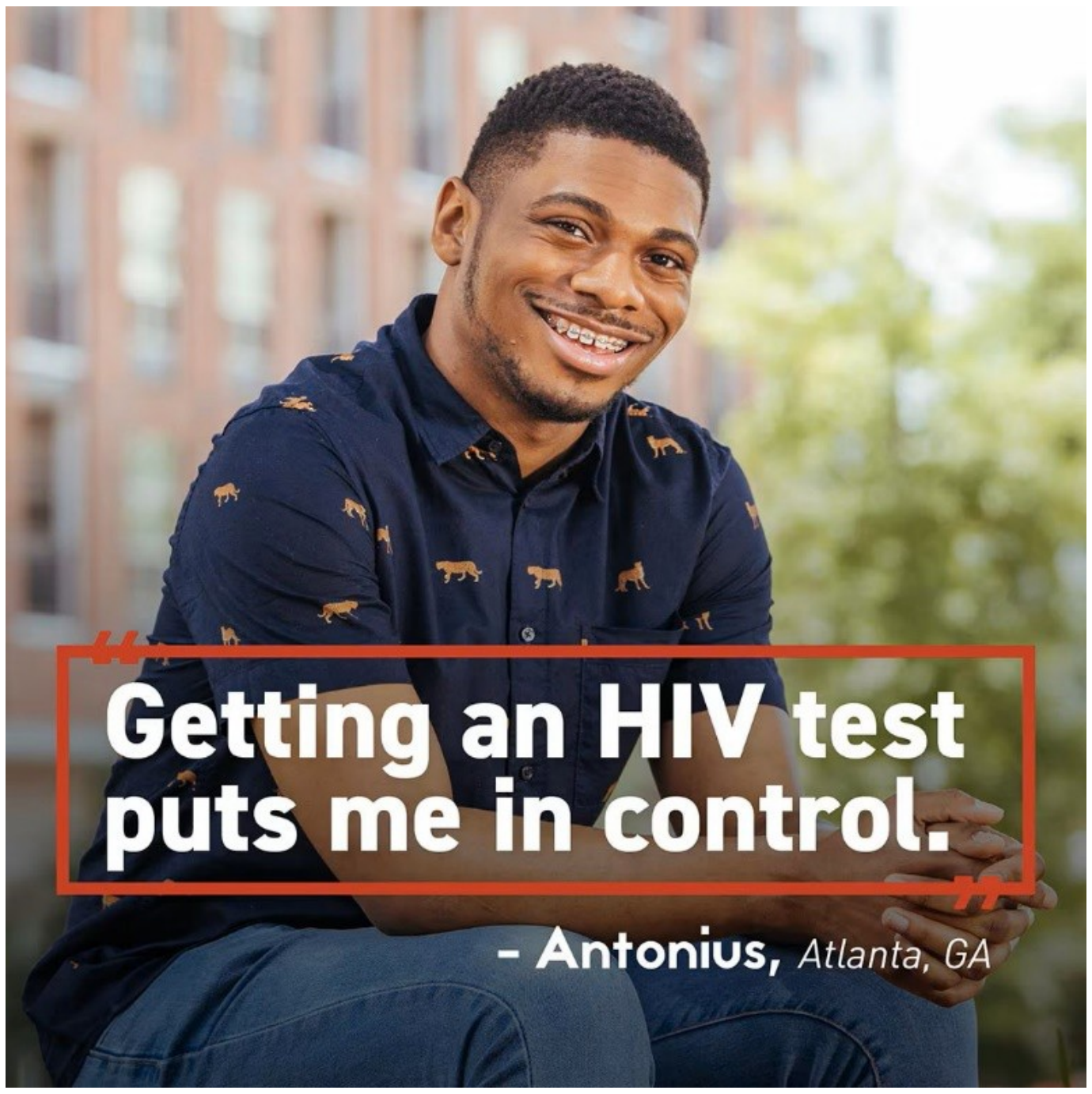

UCSF and the San Francisco VA Health Care System seek Veteran volunteers to participate in a paid research study.

Our goal is to learn about Veteran interest and experience using vending machines to access human immunodeficiency virus (HIV) self-tests.

Study participants will:

For Survey

- ✓ Use their assigned card or pin number to access to the VA Harm Reduction Vending Machine
- ✓ Obtain an HIV self-test from the VA Harm Reduction Vending Machine
- ✓ Look for the paid research study form attached to the HIV self-test
- ✓ Scan the QR code or go to the website provided on the form to take a confidential online survey (participants who forgo payment will be anonymized).
- ✓ (Optional): Provide your name and email at the end of the survey to receive a $20 electric gift card. There is no limit to the number of times the survey can be taken, but participants will only be paid for 2 surveys or up to $40.

For Interview

- ✓ Participants who use the HIV self-test may also refer themselves to take part in a one-time 1-hour interview.
- ✓ Interview participants will be paid a $100 gift card

Need access to the VA Harm Reduction Vending Machine? Have questions or concerns? Contact pharmacist [study PI, phone, email].

Interested in learning about our research team and projects? Scan this QR code or visit our web site at [insert website].

UCSF University of California San Francisco

VA | U.S. Department of Veterans Affairs
Veterans Health Administration
San Francisco VA Health Care System

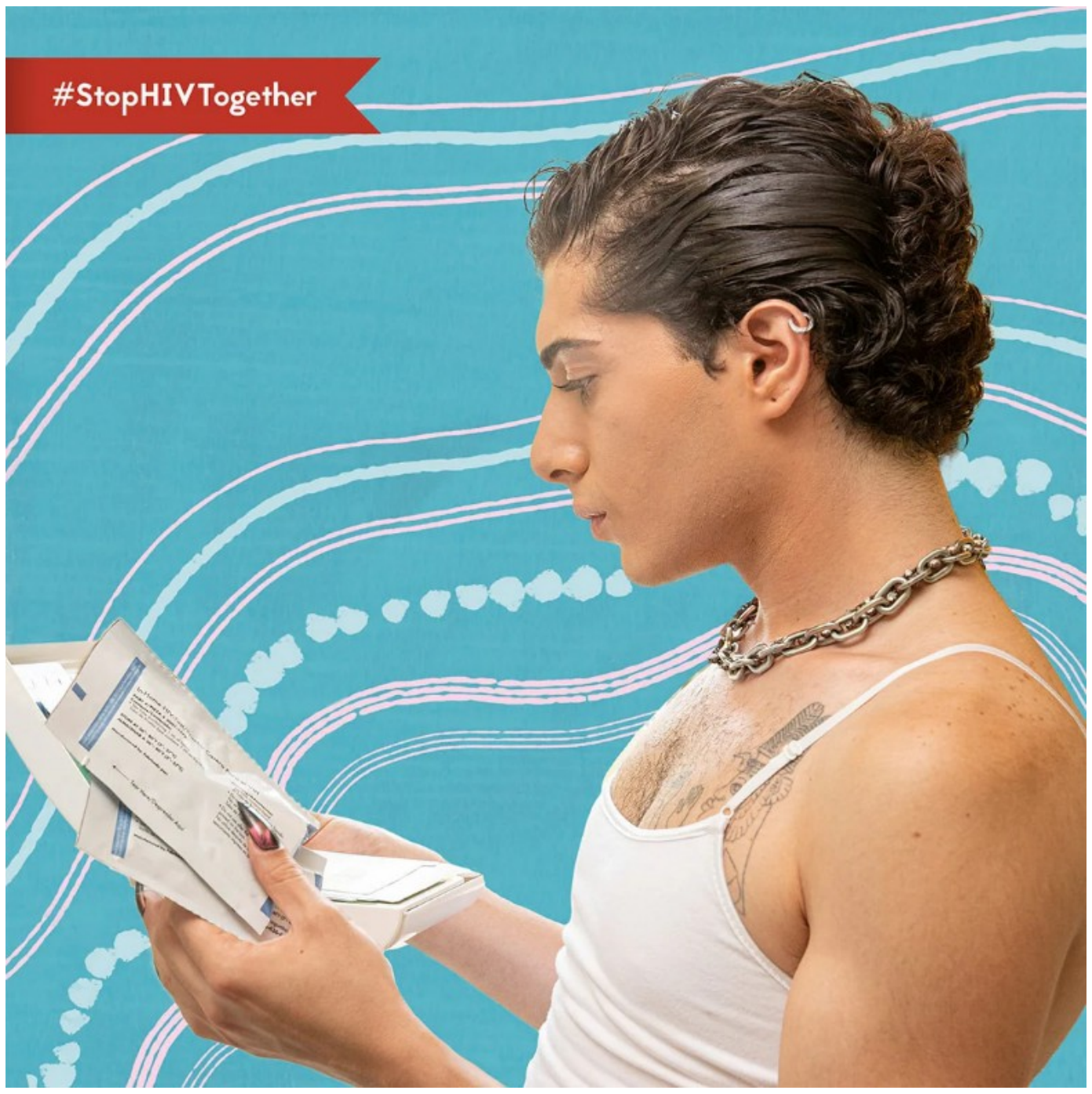

UCSF and the San Francisco VA Health Care System seek Veteran volunteers to participate in a **paid** research study.

Our goal is to learn about Veteran interest and experience using vending machines to access human immunodeficiency virus (HIV) self-tests.

Study participants will:

For Survey

- ✓ Use their assigned card or pin number to access to the VA Harm Reduction Vending Machine
- ✓ Obtain an HIV self-test from the VA Harm Reduction Vending Machine
- ✓ Look for the paid research study form attached to the HIV self-test
- ✓ Scan the QR code or go to the website provided on the form to take a confidential online survey (participants who forgo payment will be anonymized).
- ✓ (Optional): Provide your name and email at the end of the survey to receive a $20 electric gift card. There is no limit to the number of times the survey can be taken, but participants will only be paid for 2 surveys or up to $40.

For Interview

- ✓ Participants who use the HIV self-test may also refer themselves to take part in a one-time 1-hour interview.
- ✓ Interview participants will be paid a $100 gift card

Need access to the VA Harm Reduction Vending Machine? Have questions or concerns? Contact pharmacist [study PI, phone, email].

Interested in learning about our research team and projects? Scan this QR code or visit our web site at [insert website].

UCSF University of California San Francisco

VA | U.S. Department of Veterans Affairs

Veterans Health Administration
San Francisco VA Health Care System

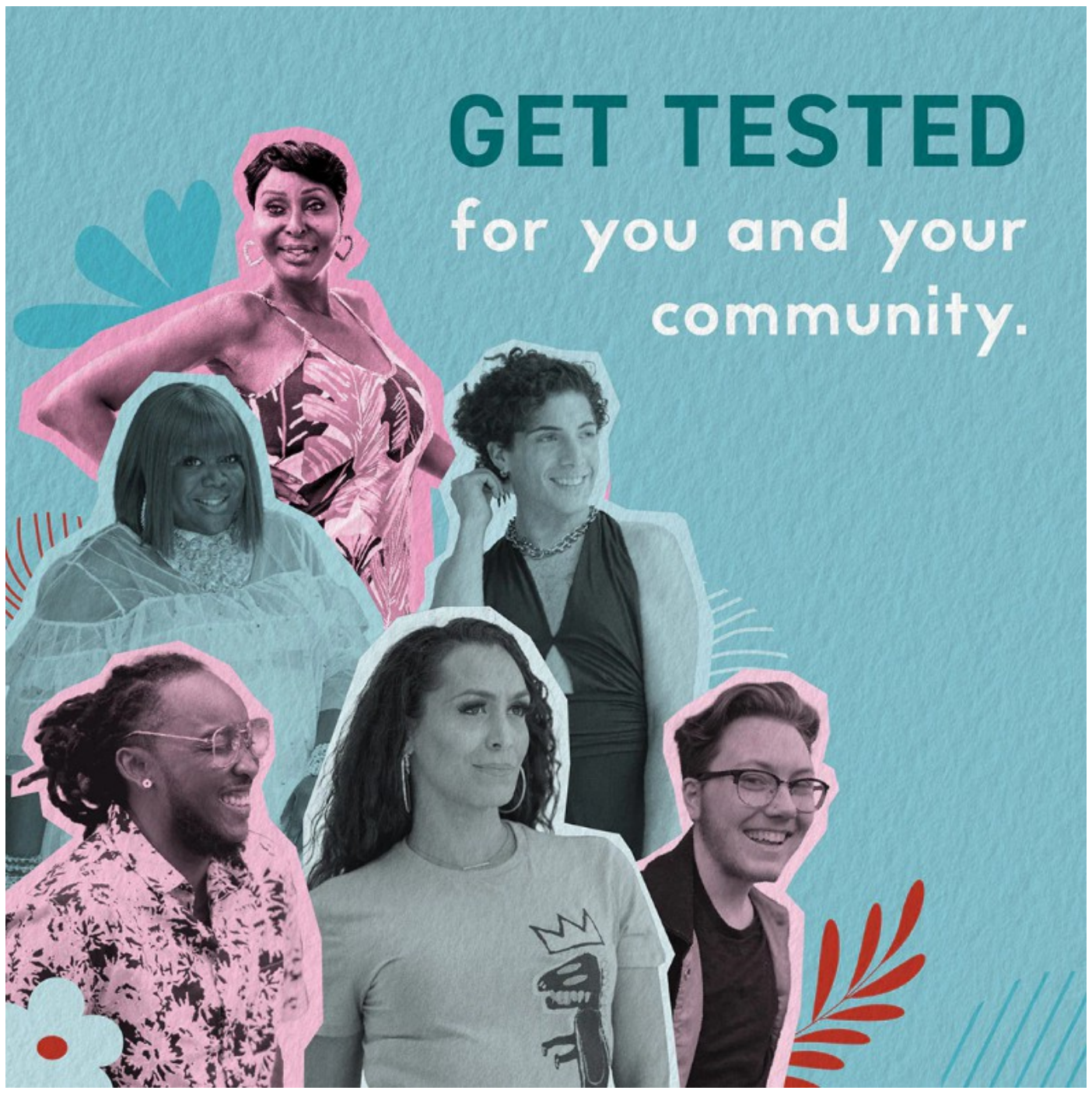

UCSF and the San Francisco VA Health Care System seek Veteran volunteers to participate in a paid research study.

Our goal is to learn about Veteran interest and experience using vending machines to access human immunodeficiency virus (HIV) self-tests.

Study participants will:

For Survey

- ✓ Use their assigned card or pin number to access to the VA Harm Reduction Vending Machine
- ✓ Obtain an HIV self-test from the VA Harm Reduction Vending Machine
- ✓ Look for the paid research study form attached to the HIV self-test
- ✓ Scan the QR code or go to the website provided on the form to take a confidential online survey (participants who forgo payment will be anonymized).
- ✓ (Optional): Provide your name and email at the end of the survey to receive a $20 electric gift card. There is no limit to the number of times the survey can be taken, but participants will only be paid for 2 surveys or up to $40.

For Interview

- ✓ Participants who use the HIV self-test may also refer themselves to take part in a one-time 1-hour interview.
- ✓ Interview participants will be paid a $100 gift card

Need access to the VA Harm Reduction Vending Machine? Have questions or concerns? Contact pharmacist [study PI, phone, email].

Interested in learning about our research team and projects? Scan this QR code or visit our web site at [insert website].
