# Supplementary material for: Optimizing Veteran-Facing Materials for Vending Machine-Dispensed HIV Self-Testing: Formative Qualitative Study
Source: JMIR Form Res. 2026 Jul 23;10:e93868. doi: 10.2196/93868 (PMC13448198; doi:10.2196/93868)
Supplement: Supplementary file 2 [file formative_v10i1e93868_app3.pdf]

HIV 101

Without treatment, HIV (human immunodeficiency virus) can make a person very sick and even cause death. Learning the basics about HIV can keep you healthy and prevent transmission.

HIV CAN BE TRANSMITTED BY

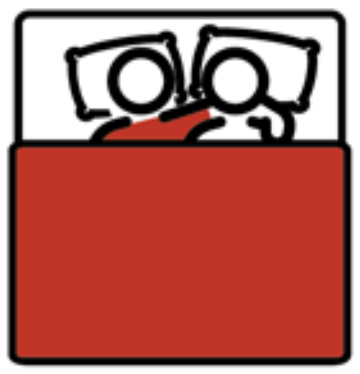

Sexual Contact

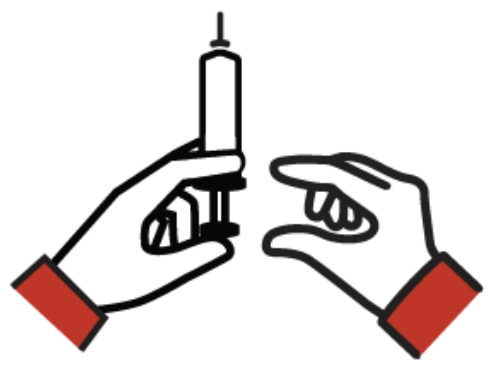

Sharing Needles to Inject Drugs

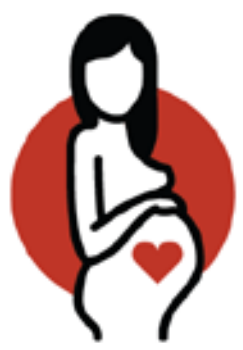

During Pregnancy, Birth, or Breast/Chestfeeding

HIV IS NOT TRANSMITTED BY

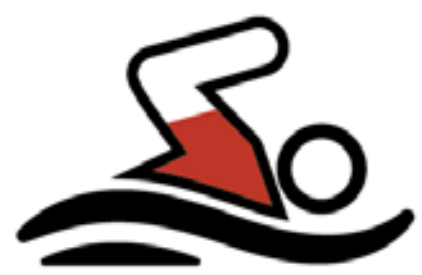

Air or Water

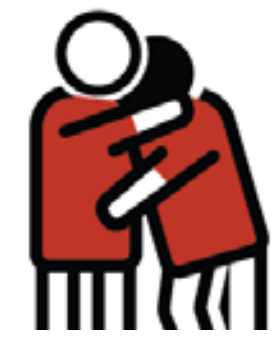

Saliva, Sweat, Tears, or Closed-Mouth Kissing

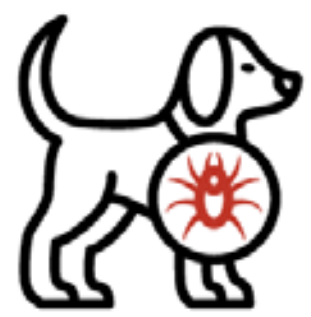

Insects or Pets

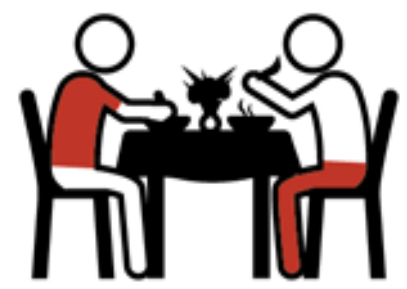

Sharing Toilets, Food, or Drinks

PROTECT YOURSELF FROM HIV

- Get tested at least once or more often if you have certain risk factors.
- Use condoms the right way every time you have anal or vaginal sex.
- Choose activities with little to no risk like oral sex.
- Don't inject drugs, or if you do, don't share needles, syringes, or other drug injection equipment.

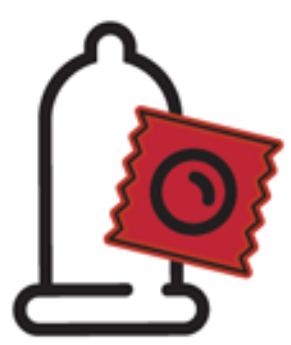

- If you engage in behaviors that may increase your chances of getting HIV, ask your health care provider if pre-exposure prophylaxis (PrEP) is right for you.

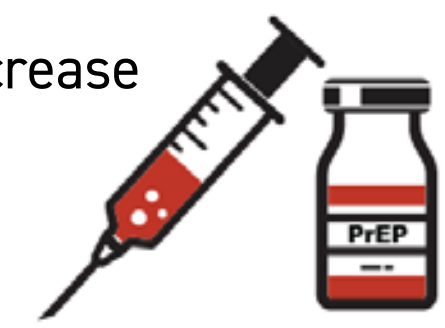

- If you think you've been exposed to HIV within the last 3 days, ask a health care provider about post-exposure prophylaxis (PEP) right away. PEP can prevent HIV, but it must be started within 72 hours.
- Get tested and treated for other STDs.

KEEP YOURSELF HEALTHY AND PROTECT OTHERS IF YOU HAVE HIV

- Find HIV care and stay in HIV care.
- Take your HIV treatment as prescribed.
- Get and keep an undetectable viral load. This is the best way to stay healthy and protect others.

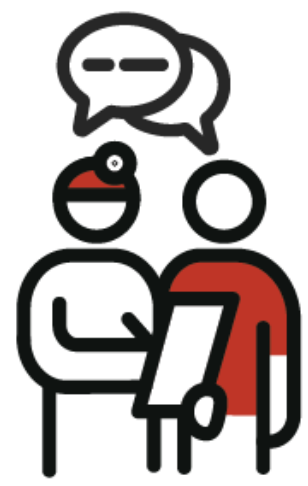

- If you have an undetectable viral load, you will not transmit HIV through sex.
- If your viral load is not undetectable—or does not stay undetectable—you can still protect your partners by using other HIV prevention options.

Scan to learn more!

LET'S STOP HIV TOGETHER™

Ending the HIV Epidemic

INFORMACIÓN BÁSICA SOBRE EL VIH

Sin tratamiento, el VIH (virus de la inmunodeficiencia humana) puede hacer que una persona esté muy enferma, e incluso causarle la muerte. Aprender lo básico sobre el VIH puede mantenerlo saludable y prevenir la transmisión de este virus.

EL VIH PUEDE SER TRANSMITIDO

- Mediante el contacto sexual
- Al compartir las agujas para inyectarse drogas
- Durante el embarazo, el parto o el pecho/lactancia materna

EL VIH NO SE TRANSMITE

- A través del aire o del agua
- Mediante la saliva, el sudor, las lágrimas o los besos con la boca cerrada
- Por los insectos o por las mascotas
- Al compartir el inodoro, los alimentos o las bebidas

PROTÉJASE DEL VIH

- Hágase la prueba al menos una vez o con más frecuencia si tiene ciertos factores de riesgo.
- Use condones de la manera correcta cada vez que tenga relaciones sexuales anales o vaginales.
- Elija actividades que impliquen poco o nada de riesgo, como las relaciones sexuales orales.
- No se inyecte drogas, pero si lo hace, no comparta las agujas, jeringas, u otro equipo de inyección de drogas.
- Si tiene comportamientos que pueden aumentar sus posibilidades de contraer el VIH, pregúntele a su proveedor de atenció médica si la profilaxis preexposicíon (PrEP) es adecuada para usted.
- Si cree que se ha expuesto al VIH dentro delos últimos 3 días, pregúntele de inmediato a un proveedor de atención médica acerca de la profilaxis posexposición (PEP). La PEP puede prevenir el VIH, pero debe comenzarse dentro de las 72 horas de la posible exposición.
- Hágase las pruebas de detección de otras ETS y reciba el tratamiento necesario.

SI TIENE EL VIH, MANTÉNGASE SALUDABLE Y PROTEJA A LOS DEMÁS

- Busque atención médica para el VIH y no deje de recibir la atención médica para el VIH.
- Tomar el tratamiento para el VIH según las indicaciones.
- Obtenga y mantenga una carga viral indetectable. Esta es la mejor manera de mantenerse saludable y proteger a los demás.
- Si tiene una carga viral indetectable, no transmitirá el VIH a su pareja sexual.
- Si su carga viral no es indetectable, o no permanece indetectable, aún puede proteger a sus parejas utilizando otras opciones de prevención del VIH.

¡Escanea para obtener más información!

DETENGAMOS JUNTOS EL VIH™ | Ending the HIV Epidemic

Para obtener más información, visite la página **www.cdc.gov/hiv/spanish.**

MLS-280326_r

March 2017 | www.hiv.va.gov

Rapid Oral HIV Test

Patient Fact Sheet

What is a rapid oral HIV test?

This is a test for HIV, the virus that causes AIDS. With a rapid oral test, results take about 20 minutes. There are also rapid tests that use a blood sample. Rapid tests are usually used when results need to be delivered within a few minutes, like in an outreach center or homeless clinic; otherwise a traditional blood test is used. The rapid oral test is quite good at detecting chronic (longstanding) HIV infection but not so good at detecting very recent HIV infection.

How does the rapid oral HIV test work?

When HIV enters the body, antibodies are produced. The test looks for HIV antibodies in your oral fluids. The test uses a swab to collect a sample from the inside of your mouth.

What happens when you agree to be tested?

- The test is explained to you by a health provider.
- A health provider will ask you to swab your gums with a special swab.
- Results are ready in about 20 minutes.
- You will learn your HIV result and discuss what it means. Your provider will give you information about how to protect yourself and others from HIV.
- If your test result is positive, you will do a second, different, test using a blood sample that is sent to the lab.
- Your test result will be confidential (results will only be discussed with you). Your test result will not affect your VA benefits.

What does a negative rapid oral HIV test result mean?

This means that HIV antibodies have not been found in your system. This could mean one of two things:

1. You do not have HIV, or
2. You have HIV but it was not detected by the rapid oral test (this is a false-negative result).

It can take up to 3 months for your system to produce enough antibodies to be detected by the rapid oral test. If you have engaged in activities that might put you at risk of HIV infection in the past 3 months, you should repeat the test in a few weeks, or talk with your VA provider about doing a standard blood test for HIV right away.

What does a positive rapid oral HIV test result mean?

This means that HIV antibodies may be in your system. Positive results on the rapid test must always be confirmed by doing a second HIV test. This is because sometimes (rarely) the rapid oral test gives a false-positive result. A person is considered to be HIV positive only if two different test results are positive. The second HIV test is a blood test that is sent to the lab; the results may take up to 1-2 weeks to return.

VA | U.S. Department of Veterans Affairs
Veterans Health Administration

If your positive result is confirmed by a second test, that means:

- You have HIV and should be evaluated right away for treatment
- Your testing site will refer you to a clinic that has expertise in caring for people with HIV. They may also refer you for counseling and other support services.
- HIV treatment (medication) greatly improves health, and can prevent transmission (infection) to others

Why should you get tested?

- Many HIV-positive people do not know they are infected with HIV, because they have never been tested.
- Knowing your HIV status helps you protect yourself and others.
- Getting diagnosed early can greatly improve your health. Although there is no cure for HIV, there are many effective medicines that can control it. Most people with HIV who take their medication every day can live long, healthy lives.
- If you test negative, you may feel less anxious after testing, and you may be more committed to preventing yourself from becoming infected.
- An HIV test is part of routine medical care.

For more information about HIV Testing, please visit **http://www.hiv.va.gov**

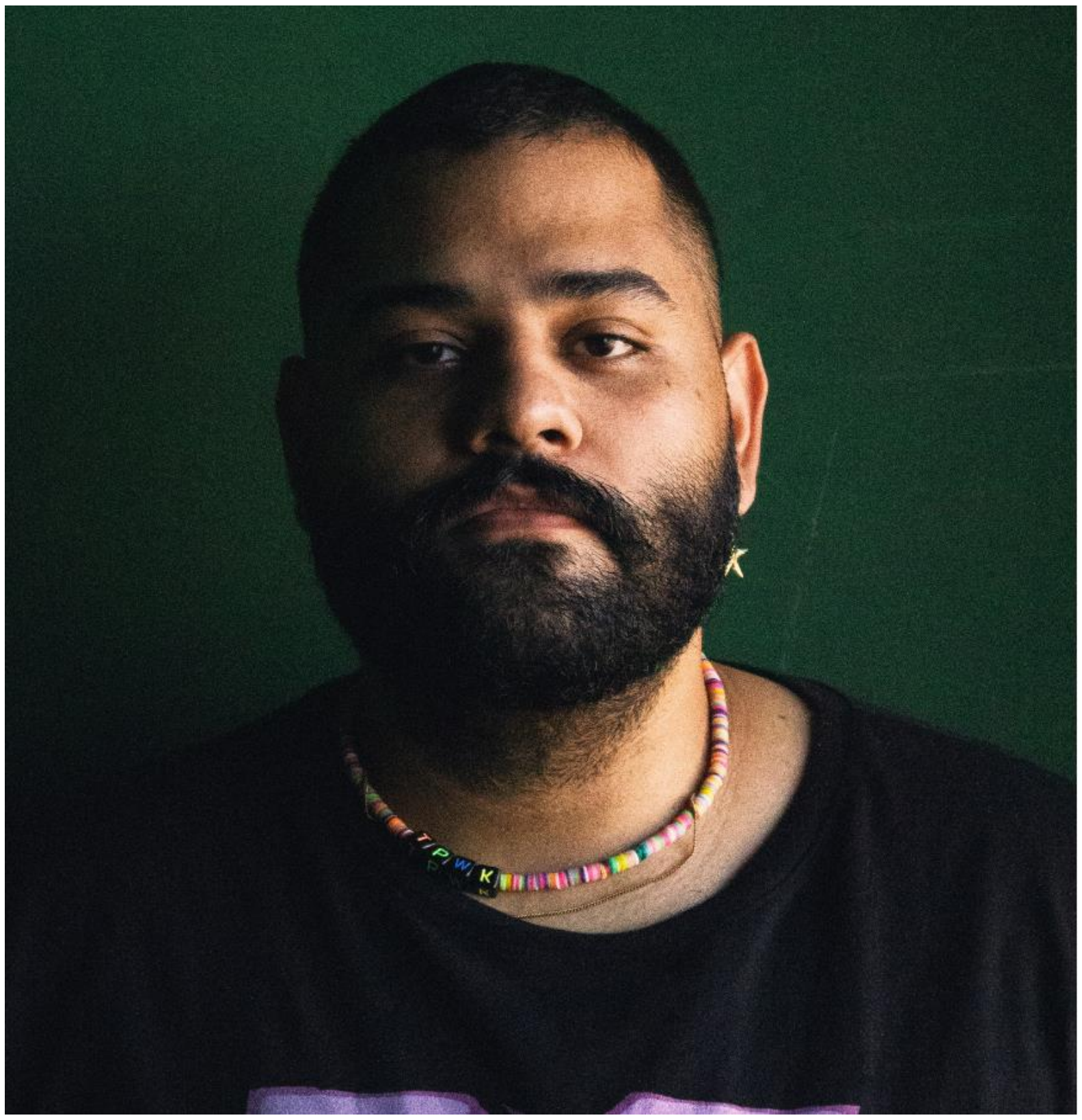

Testing that meets you where you are.

Literally.

TAKEMEHOME.ORG

What is TakeMeHome?

TakeMeHome is a **FREE** sexual health home testing program. The program provides free HIV tests, STI tests, and PrEP panel tests (where available) that you can take in a place you're most comfortable without going to a clinic or testing site.

How does TakeMeHome work?

TakeMeHome wants to make getting tested as easy and simple as possible. Go to the website: https://takemehome.org. You will be asked a few questions to see if you are eligible to use the free service. If you are eligible, you will be able order your home test kit in less than five minutes.

What comes in a kit?

TakeMeHome has a few different options. Only some may be available in your area. See the back side of this sheet for more details.

HIV FINGER STICK TEST

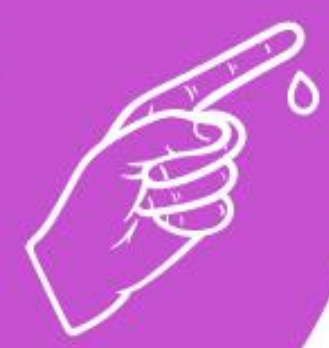

INCLUDES

- 1 HIV Fingerstick + Blood Test Card

TESTS FOR

- HIV

HIV ORAL SWAB TEST

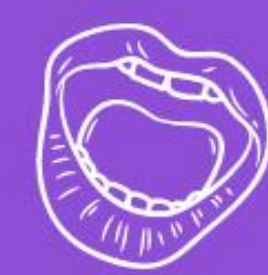

INCLUDES

- 1 OraQuick Oral Swab

TESTS FOR

- HIV

PREP PANEL TEST

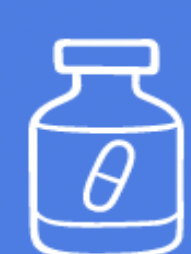

INCLUDES

- 1 PrEP Panel Test
- 1 HIV Fingerstick Test + Blood Test Card

TESTS FOR

- Creatinine Levels for PrEP Users (kidney function)
- HIV

STI + HIV TEST

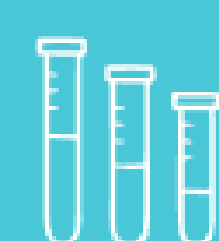

INCLUDES

- 1 Rectal Swab
- 1 Throat Swab
- 1 Urine Sample Cup
- 1 HIV Fingerstick Test + Blood Test Card

TESTS FOR

- Chlamydia, Gonorrhea (rectal)
- Chlamydia, Gonorrhea (throat)
- Chlamydia, Gonorrhea (urine)
- HIV
- Syphilis Hepatitis C*

Need HIV testing, sexual health, or harm reduction resources?

Contact your VA primary care provider or Harm Reduction Pharmacist [Name and Phone].

Here are some additional resources in Alameda County:

Organization	1. Berkeley Free Clinic	1. Needle Exchange Emergency Distribution (NEED)	2. Oakland Punks with Lunch	3. AIDS Healthcare Foundation	4. Hideout Honeycomb 510	5. AIDS Healthcare Foundation	6. Oakland LGBTQ Community Center, Glenn Burke Wellness Clinic	7. La Clinica	8. La Clinica	9. HIV Education & Prevention Project of Alameda County	10. Planned Parenthood
**Address**	2339 Durant Ave Berkeley, CA 94704		1924 Union St, Oakland 94706	400 30th St Oakland, CA 94609	310 8th St Oakland, CA 94607	238 E 18th St Oakland, CA 94606	3207 Lakeshore Ave Oakland, CA 94610	1030 International Blvd Oakland, CA 94606	3451 East 12th St Oakland, CA 94601	5323 Foothill Blvd Oakland, CA 94601	8480 Enterprise Way Oakland, CA 94621
**Phone**	510-548-2570	510-463-1843	510-371-4465	510-628-0949	510-972-4483	510-251-8678	510-882-2286	510-238-5400	510-535-3319	510-434-0307	510-746-4700
**HIV Testing**	✓			✓	✓	✓	✓	✓	✓	✓	✓
**HIV PrEP**				✓	✓	✓	✓	✓	✓		✓
**PEP**				✓	✓	✓	✓	✓	✓		✓
**STI Testing & Treatment**	✓			✓	✓	✓	✓	✓	✓		✓
**Condoms**		✓	✓	✓	✓	✓	✓	✓	✓	✓	✓
**HCV Testing & Treatment**	✓			✓	✓	✓	✓	✓	✓	✓	
**Abortion**											✓
**Birth control**								✓	✓		✓
**Emergency contraception**											✓
**Gender-affirming care**							✓	✓	✓		✓
**Pregnancy testing & planning**							✓	✓	✓		✓
**Sexual & reproductive concerns**							✓	✓	✓		✓

Organization	1. Berkeley Free Clinic	1. Needle Exchange Emergency Distribution (NEED)	2. Oakland Punks with Lunch	3. AIDS Healthcare Foundation	4. Hideout Honeycomb 510	5. AIDS Healthcare Foundation	6. Oakland LGBTQ Community Center, Glenn Burke Wellness Clinic	7. La Clinica	8. La Clinica	9. HIV Education & Prevention Project of Alameda County	10. Planned Parenthood
**Vaccines**	✓			✓		✓	✓	✓	✓		✓
**Wellness & preventive care**				✓		✓	✓	✓	✓		✓
**Substance use treatment**								✓	✓		
**Safer injection supplies & disposal**	✓	✓	✓							✓	
**Fentanyl testing supplies**	✓	✓	✓							✓	
**Naloxone**	✓	✓	✓							✓	
**Cost/Payment**	Free	Free	Free	Ins, Medicare	Free, Ins, low cost	Ins, Medicare		Ins, SS	Ins, SS	Free	Ins, SS, Medicaid

HIV = human immunodeficiency virus

STI = sexually transmitted infection

HIV PrEP = HIV pre-exposure prophylaxis, a medication to prevent HIV through sex or injection drug use

PEP = post-exposure prophylaxis, medication to treat HIV or STIs after possible exposure

HCV = hepatitis C virus

Ins = insurance accepted

SS = sliding scale

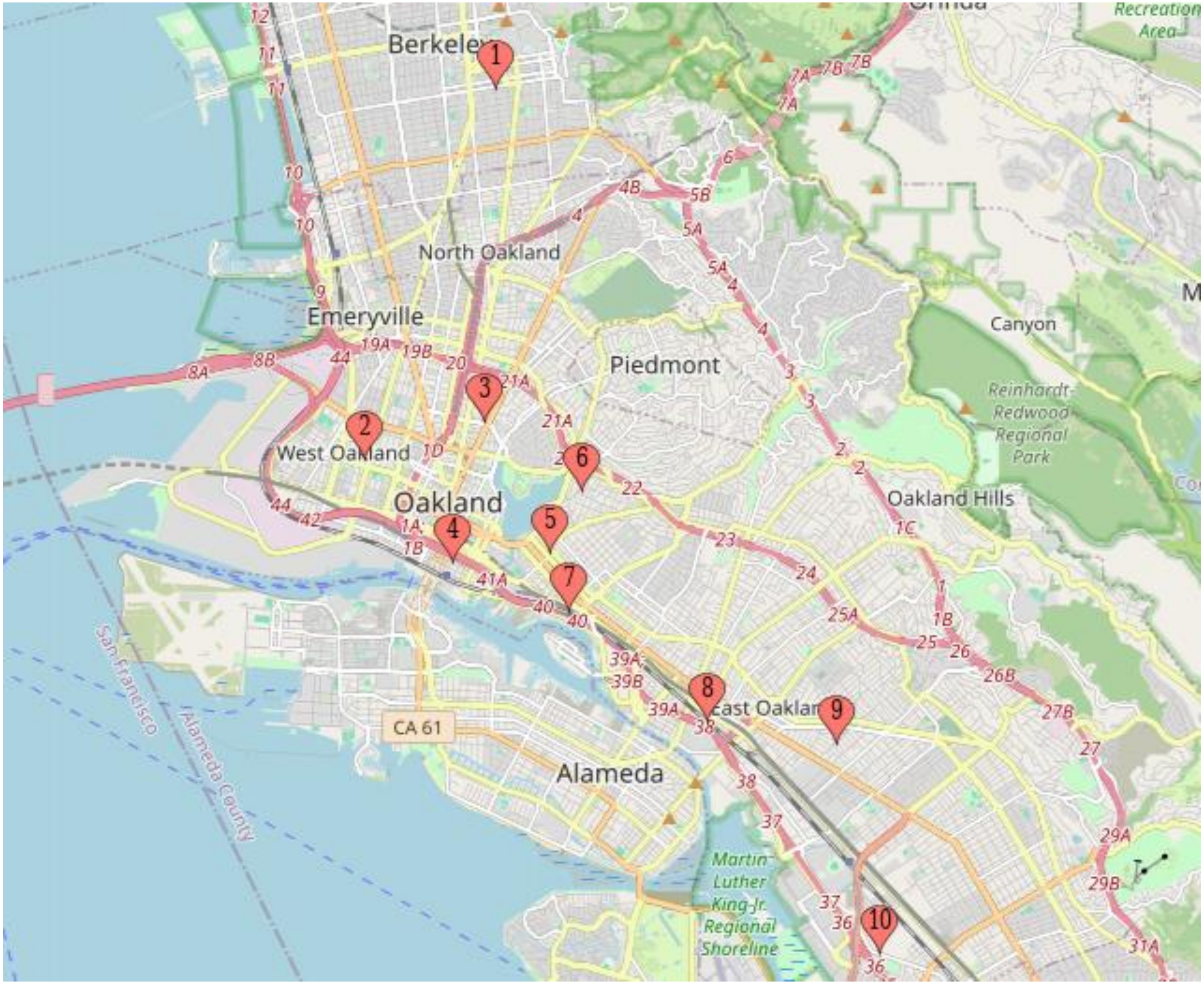

North Oakland
Emeryville
Piedmont
Oakland
Alameda
Canyon
Oakland Hills
Reinhardt-Redwood Regional Park
Martin Luther King Jr. Regional Shoreline
CA 61
San Francisco
Alameda County
Recreation Area
1
2
3
4
5
6
7
8
9
10

Need HIV testing, sexual health, or harm reduction resources?

Contact your VA primary care provider or Harm Reduction Pharmacist [Name and Phone].

Here are some additional resources in Humboldt County:

**Organization**	North Coast AIDS Project (NorCAP) Syringe Services	Humboldt County Dept. of Health & Human Services	Planned Parenthood Northern California	Eureka Community Health & Wellness Center	Redwood Community Health Center Family Medicine & Pregnancy Services	Arcata Community Health Center	Mckinleyville Community Health Center	Humboldt Area Center for Harm Reduction
**Address**	908 7th Street, Eureka 95501	529 I St Eureka, CA 95501	3225 Timber Fall Ct Ste B Eureka, CA 95503	2200 Tydd St Eureka, CA 95501	2350 Buhn St Ste A Eureka, CA 95501	1150 Foster Ave Arcata, CA 95521	1644 Central Ave Mckinleyville, CA 95519	Mobile outreach in Eureka and Arcata
**Phone**	707-268-2132	707-268-2108	707-442-5700	707-441-1624	707-443-4593	707-826-8610	707-839-3068	707-407-6013
**HIV Testing**	✓	✓	✓	✓	✓	✓	✓	
**HIV PrEP**	✓		✓	✓				
**PEP**			✓	✓				
**STI Testing & Treatment**		✓	✓	✓	✓	✓	✓	
**Condoms**	✓	✓	✓	✓	✓	✓	✓	
**HCV Testing & Treatment**	✓			✓				
**Abortion**			✓					
**Birth control**			✓					
**Emergency contraception**			✓					
**Gender-affirming care**			✓	✓				
**Pregnancy testing & planning**			✓	✓	✓			
**Sexual & reproductive concerns**			✓	✓	✓			
**Vaccines**		✓	✓	✓	✓	✓	✓	

Organization	North Coast AIDS Project (NorCAP) Syringe Services	Humboldt County Dept. of Health & Human Services	Planned Parenthood Northern California	Eureka Community Health & Wellness Center	Redwood Community Health Center Family Medicine & Pregnancy Services	Arcata Community Health Center	Mckinleyville Community Health Center	Humboldt Area Center for Harm Reduction
**Wellness & preventive care**			✓	✓	✓	✓	✓	
**Substance use treatment**	✓			✓	✓	✓	✓	
**Safer injection supplies & disposal**	✓							✓
**Fentanyl testing supplies**								✓
**Naloxone**	✓							✓
**Cost/Payment**	Free		Ins, Medicaid, SS	Ins, Medicaid, SS	Ins, Medicaid, SS	Ins, Medicaid, SS	Ins, Medicaid, SS	

HIV = human immunodeficiency virus

STI = sexually transmitted infection

HIV PrEP = HIV pre-exposure prophylaxis, a medication to prevent HIV through sex or injection drug use

PEP = post-exposure prophylaxis, medication to treat HIV or STIs after possible exposure

HCV = hepatitis C virus

Ins = insurance accepted

SS = sliding scale

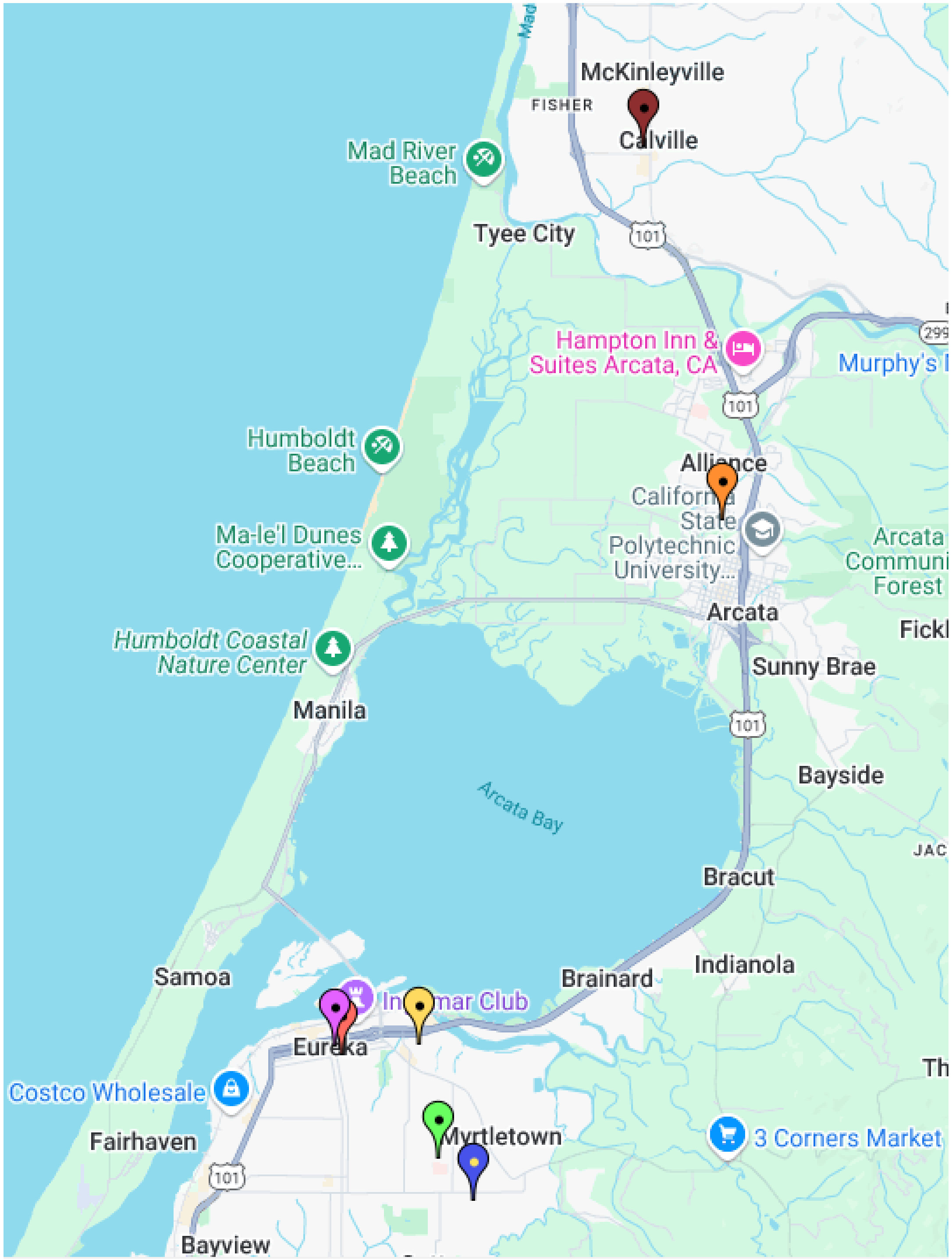
McKinleyville
FISHER
Calville
Mad River Beach
Tyee City
101
Hampton Inn & Suites Arcata, CA
Murphy's
299
Humboldt Beach
Alliance
California State Polytechnic University...
Ma-le'l Dunes Cooperative...
Arcata Community Forest
Arcata
Humboldt Coastal Nature Center
Sunny Brae
Manila
Bayside
Arcata Bay
Bracut
Samoa
Indianola
Brainard
Eureka
Costco Wholesale
Fairhaven
Myrtletown
3 Corners Market
Bayview

Need HIV testing, sexual health, or harm reduction resources?

Contact your VA primary care provider or Harm Reduction Pharmacist [Name and Phone].

Here are some additional resources in Lake & Mendocino Counties:

Organization	Planned Parenthood Northern California	Any Positive Change	Mendocino Community Health Clinic	Mendocino County AIDS/Viral Hepatitis Network (MCAVHN) Care and Prevention Network
Address	14671 Olympic Dr<br>Clearlake, CA 95422	8050 Lake St<br>Lower Lake, CA 95457	45 Hazel St<br>Willits, CA 95490	148 Clara Ave<br>Ukiah, CA 95482
Phone	707-995-2261	707-480-7319	707-456-9600	707-462-1932
HIV Testing	✓		✓	✓
HIV PrEP	✓		✓	Referrals
PEP	✓		✓	Referrals
STI Testing & Treatment	✓	Referrals	✓	
Condoms	✓	✓	✓	✓
HCV Testing & Treatment		Referrals	✓	✓
Abortion	✓			
Birth control	✓		✓	
Emergency contraception	✓			
Gender-affirming care	✓			
Pregnancy testing & planning	✓		✓	
Sexual & reproductive concerns	✓		✓	
Vaccines	✓		✓	
Wellness & preventive care	✓		✓	Referrals
Substance use treatment		Referrals	✓	✓
Safer injection supplies & disposal		✓		✓
Fentanyl testing supplies		✓		✓
Naloxone		✓		✓
Cost/Payment	Ins, Medicaid, SS	Free	Ins, Medicaid	Free

HIV = human immunodeficiency virus
STI = sexually transmitted infection
HIV PrEP = HIV pre-exposure prophylaxis, a medication to prevent HIV through sex or injection drug use
PEP = post-exposure prophylaxis, medication to treat HIV or STIs after possible exposure
HCV = hepatitis C virus
Ins = insurance accepted
SS = sliding scale

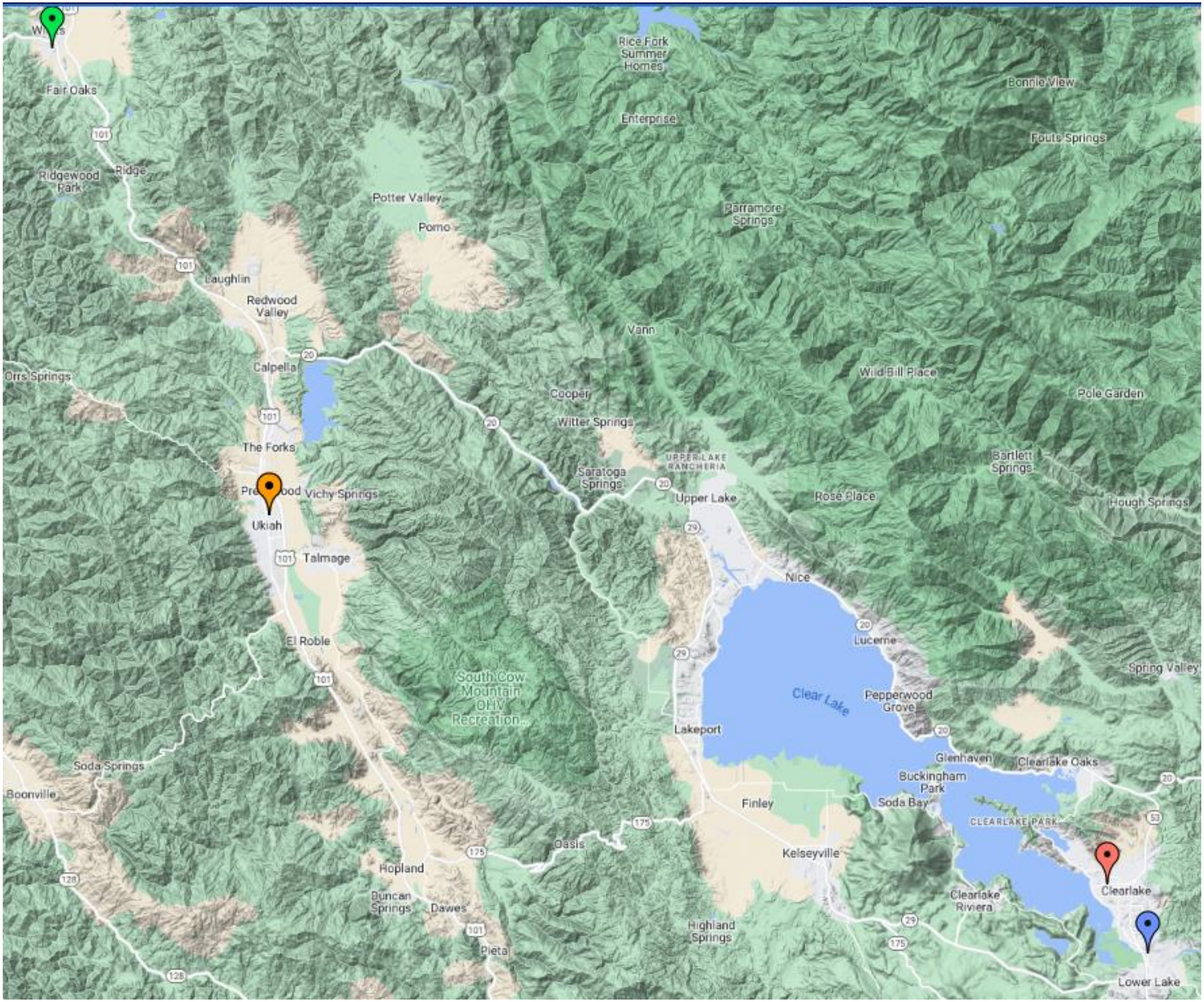

Fair Oaks
Ridgewood Park
Ridge
Laughlin
Redwood Valley
Potter Valley
Pomo
Rice Fork Summer Homes
Enterprise
Bonnie View
Fouts Springs
Parramore Springs
Vann
Calpella
Orrs Springs
Cooper
Witter Springs
Wild Bill Place
Pole Garden
The Forks
Vichy Springs
Saratoga Springs
UPPER LAKE RANCHERIA
Upper Lake
Rose Place
Bartlett Springs
Hough Springs
Ukiah
Talmage
Nice
Lucerne
El Roble
South Cow Mountain OHV Recreation...
Clear Lake
Pepperwood Grove
Spring Valley
Lakeport
Glenhaven
Clearlake Oaks
Buckingham Park
Soda Bay
CLEARLAKE PARK
Soda Springs
Boonville
Finley
Oasis
Kelseyville
Hopland
Clearlake
Clearlake Riviera
Duncan Springs
Dawes
Highland Springs
Pieta
Lower Lake

Need HIV testing, sexual health, or harm reduction resources?

Contact your VA primary care provider or Harm Reduction Pharmacist [Name and Phone].

Here are some additional resources in San Francisco County:

Cost/ Payment	Location	Address	Hours of Operation	HIV Testing	STI Testing & Treatment	HIV PrEP	PEP	Condoms	Mpox Vaccine	HCV Testing & Treatment
Free	San Francisco AIDS Foundation	470 Castro St<br>San Francisco, CA 94114	Tue-Sat 10A-7P	✓	✓	✓	✓	✓	✓	✓
Free	San Francisco Department of Public Health	356 7th St<br>San Francisco, CA 94103	Mon-Fri by appointment	✓	✓	✓	✓	✓	✓	
		2403 Keith St<br>San Francisco, CA 94124	Mon-Fri 8A-5P	✓	✓	✓			✓	✓
		230 Golden Gate Ave<br>San Francisco, CA 94102	Mon-Wed, Fri 8A-5P<br>Thurs 1P-5P	✓	✓	✓		✓		
		1525 Silver Ave<br>San Francisco, CA 94134	Mon-Fri 8A-6P<br>Sat 8A-4:30P	✓	✓			✓		
		1050 Wisconsin St<br>San Francisco, CA 94107	Mon-Wed, Fri 8A-5P<br>Thurs 8A-12P	✓	✓			✓		
Free	Glide	330 Ellis St<br>San Francisco, CA 94102	Every day 8:30A-5P	✓	✓			✓	✓	✓
Ins	Sutter Health	45 Castro St Ste 325<br>San Francisco, CA 94114	Mon-Fri 8A-5P	✓	✓	✓	✓	✓	✓	
SS	Mission Neighborhood Health Center	240 Shotwell St<br>San Francisco, CA 94110	Mon, Fri 8A-6P<br>Tue-Thur 8A-8P	✓	✓	✓		✓	✓	✓
SS, Ins	San Francisco Community Health Center	730 Polk St 4th Fl<br>San Francisco, CA 94109	Mon-Fri 8:45A-4:45P	✓	✓	✓	✓	✓		✓
SS, Ins	Lyon Martin Community Health	1735 Mission St<br>San Francisco, CA 94103	Mon 1P-5P<br>Tue 8:30A-7P<br>Wed 10:30A-7P<br>Thur, Fri 8:30A-5P	✓	✓	✓	✓	✓		✓
SS, Ins	AIDS Healthcare Foundation	518A Castro St<br>San Francisco CA 94114	Wed, Fri 8:30A-5:30P	✓	✓	✓	✓	✓		
SS, Ins	Planned Parenthood Northern California	1522 Bush St<br>San Francisco, CA 94109	Mon 11:30A-7P<br>Tue 8:30A-7P<br>Wed-Fri 8:30A-5P<br>Sat 8:30A-4:30P	✓	✓	✓	✓	✓		

Cost/ Payment	Location	Address	Hours of operation	HIV Testing	STI Testing & Treatment	HIV PrEP	PEP	Condoms	Mpox Vaccine	HCV Testing & Treatment
SS, Ins	Equity Health	229 7th St<br>San Francisco, CA 94103	Mon, Tue, Th 8A-5P<br>Wed 8A-7P<br>Fri 8A-3:30P	✓	✓			✓		✓
Ins	Mission Wellness Pharmacy	2424 Mission St<br>San Francisco, CA 94110	Mon-Fri 9A-6P<br>Sat 10A-2P	✓		✓	✓			✓
Ins	UCSF Medical Center	350 Parnassus St Ste 908<br>San Francisco, CA	Mon-Fri 8:30A-5P		✓	✓	✓			✓
Ins	San Francisco Health Network	3850 17th St<br>San Francisco, CA 94114	Mon-Fri 8A-6P	✓	✓	✓	✓			
SS, Ins	Native American Health Center	160 Capp St<br>San Francisco, CA 94110	Mon-Fri 8:30A-5P<br>Sat by appointment	✓	✓	✓		✓		
Ins	TM Clinic	909 Hyde St Ste 432<br>San Francisco, CA 94109	Mon-Fri 9A-5:30P	✓	✓	✓				
Ins	Dignity-GoHealth Urgent Care	199 West Portal Ave<br>San Francisco, CA 94127	Mon-Fri 8A-8P<br>Sat-Sun 9A-5P	✓	✓		✓			
		930 Cole St Ste 102<br>San Francisco, CA 94117		✓	✓		✓			
		2895 Diamond St<br>San Francisco, CA 94131		✓	✓		✓			
		1600 Market St<br>San Francisco, CA 94102		✓	✓		✓			
		2395 Lombard St<br>San Francisco, CA 94123		✓	✓		✓			
Ins/SS	Mission Neighborhood Health Center	4434 Mission St<br>San Francisco, CA 94112	Mon-Fri 8A-5P	✓	✓			✓		
		1580 Valencia St Ste 506<br>San Francisco, CA 94110	Mon-Fri 8:30A-5:30P	✓	✓					
		1663 Mission St Ste 603<br>San Francisco, CA 94103	Mon-Fri 8A-5P	✓		✓		✓		

Ins = insurance accepted
SS = sliding scale
HIV = human immunodeficiency virus
STI = sexually transmitted infection
HIV PrEP = HIV pre-exposure prophylaxis, a medication to prevent HIV through sex or injection drug use
PEP = post-exposure prophylaxis, medication to treat HIV or STIs after possible exposure
HCV = hepatitis C virus

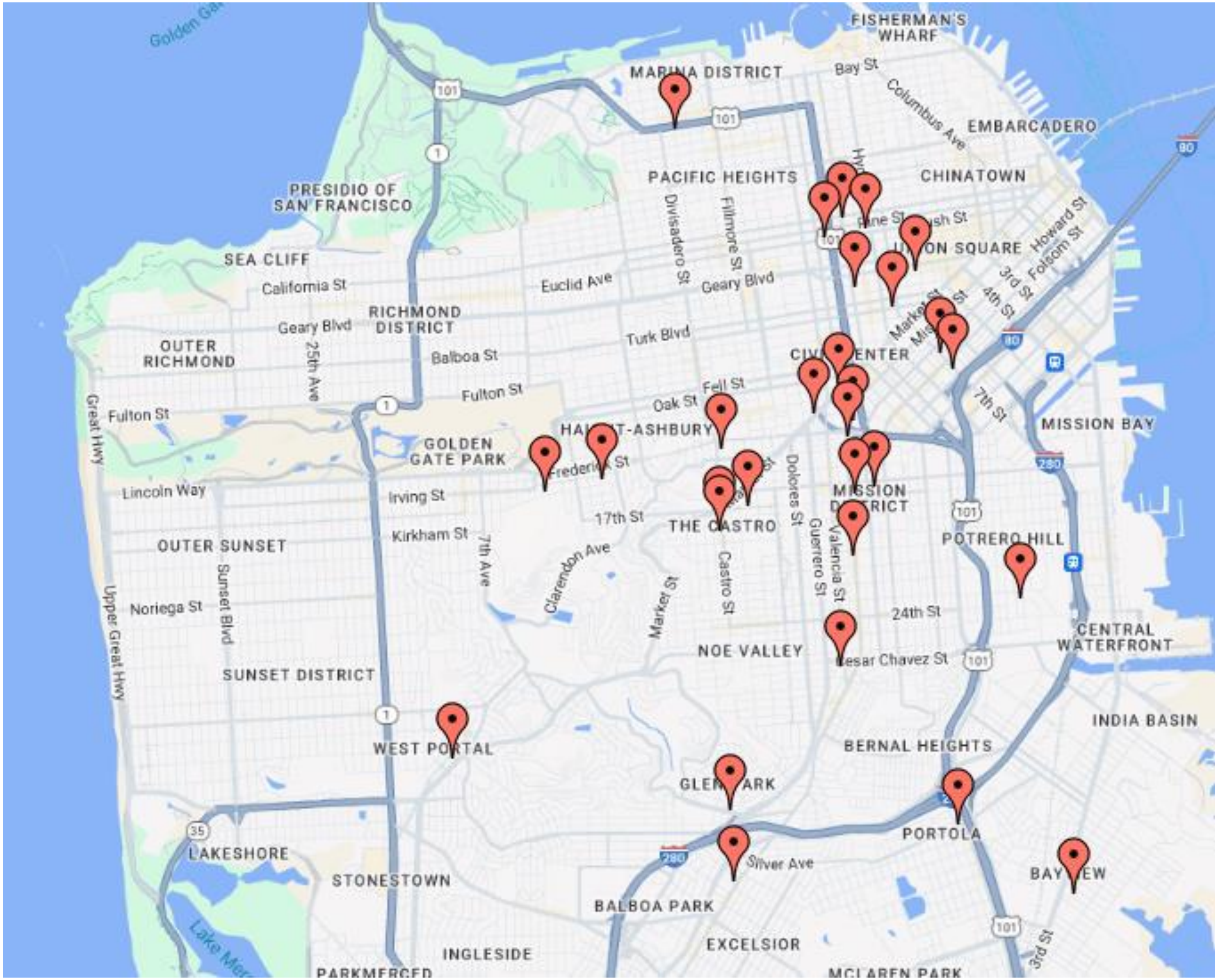
FISHERMAN'S WHARF
Bay St
Columbus Ave
EMBARCADERO
PACIFIC HEIGHTS
CHINATOWN
PRESIDIO OF SAN FRANCISCO
SEA CLIFF
California St
Euclid Ave
Divisadero St
Fillmore St
Geary Blvd
Howard St
Folsom St
3rd St
4th St
RICHMOND DISTRICT
Geary Blvd
OUTER RICHMOND
25th Ave
Balboa St
Turk Blvd
Fulton St
Fell St
Oak St
Fulton St
Great Hwy
GOLDEN GATE PARK
7th St
MISSION BAY
Lincoln Way
Irving St
17th St
THE CASTRO
Dolores St
Guerrero St
Valencia St
Kirkham St
7th Ave
OUTER SUNSET
Clarendon Ave
Market St
Castro St
POTRERO HILL
Noriega St
Sunset Blvd
Upper Great Hwy
24th St
NOE VALLEY
CENTRAL WATERFRONT
SUNSET DISTRICT
INDIA BASIN
BERNAL HEIGHTS
PORTOLA
LAKESHORE
Silver Ave
STONESTOWN
BALBOA PARK
EXCELSIOR
INGLESIDE
3rd St
101
1
80
280
35

Need HIV testing, sexual health, or harm reduction resources?

Contact your VA primary care provider or Harm Reduction Pharmacist [Name and Phone].

Here are some additional resources in Sonoma County:

Organization	Santa Rosa Community Health	Santa Rosa Community Health	Santa Rosa Community Health	Face to Face Sonoma County AIDS Network	Planned Parenthood Northern California	Sonoma County Indian Health Project	West County Health Centers	Providence Medical	Petaluma Health Center	Alliance Medical Center Inc	West County Health Centers
**Address**	751 Lombardi Ct Santa Rosa, CA 95407	1300 N Dutton Ave Santa Rosa, CA 95401	3569 Round Barn Cir Santa Rosa, CA 95403	873 2nd St Santa Rosa, CA 95404	1140 Sonoma Ave Bldg 3 Santa Rosa, CA 95405	144 Stony Point Rd Santa Rosa, CA 95401	652 Petaluma Ave Ste H Sebastopol, CA 95472	2323 Bethards Dr Santa Rosa, CA 95405	5900 State Farm Dr 2nd Fl Rohenert Park, CA 94928	8465 Old Redwood Hwy Ste 320 Windsor, CA 95492	3802 Main St Occidental, CA 95465
**Phone**	707-303-3600	707-303-3600	707-303-3600	707-544-1581	707-527-7656	707-521-4545	707-823-3166	707-542-1611	707-559-7600	707-431-1170	707-874-2444
**HIV Testing**	✓	✓	✓	✓	✓	✓	✓	✓	✓	✓	✓
**HIV PrEP**	✓	✓	✓	✓	✓		✓	✓	✓		
**PEP**	✓	✓	✓	✓	✓				✓		
**STI Testing & Treatment**	✓	✓	✓	✓	✓	✓	✓	✓	✓	✓	✓
**Condoms**	✓	✓	✓	✓	✓	✓	✓			✓	✓
**HCV Testing & Treatment**	✓	✓	✓					✓	✓		
**Abortion**					✓						
**Birth control**					✓					✓	
**Emergency contraception**					✓						
**Gender-affirming care**	✓	✓	✓		✓		✓				✓
**Pregnancy testing & planning**	✓	✓	✓		✓	✓	✓	✓	✓	✓	✓

Organization	Santa Rosa Community Health	Santa Rosa Community Health	Santa Rosa Community Health	Face to Face Sonoma County AIDS Network	Planned Parenthood Northern California	Sonoma County Indian Health Project	West County Health Centers	Providence Medical	Petaluma Health Center	Alliance Medical Center Inc	West County Health Centers
**Sexual & reproductive concerns**	✓	✓	✓		✓	✓	✓	✓	✓	✓	✓
**Vaccines**	✓	✓	✓		✓	✓	✓	✓	✓	✓	✓
**Wellness & preventive care**	✓	✓	✓		✓	✓	✓	✓	✓	✓	✓
**Substance use treatment**	✓	✓	✓			✓	✓		✓	✓	✓
**Safer injection supplies & disposal**				✓							
**Naloxone**				✓		✓					
**Cost/Payment**	Ins, Medicare, SS	Ins, Medicare, SS	Ins, Medicare, SS	Free	Ins, Medicaid, SS	Ins	Ins, Medicare, SS	Ins, Medicare	Ins, SS	Ins, SS	Ins, Medicare, SS

HIV = human immunodeficiency virus

STI = sexually transmitted infection

HIV PrEP = HIV pre-exposure prophylaxis, a medication to prevent HIV through sex or injection drug use

PEP = post-exposure prophylaxis, medication to treat HIV or STIs after possible exposure

HCV = hepatitis C virus

Ins = insurance accepted

SS = sliding scale

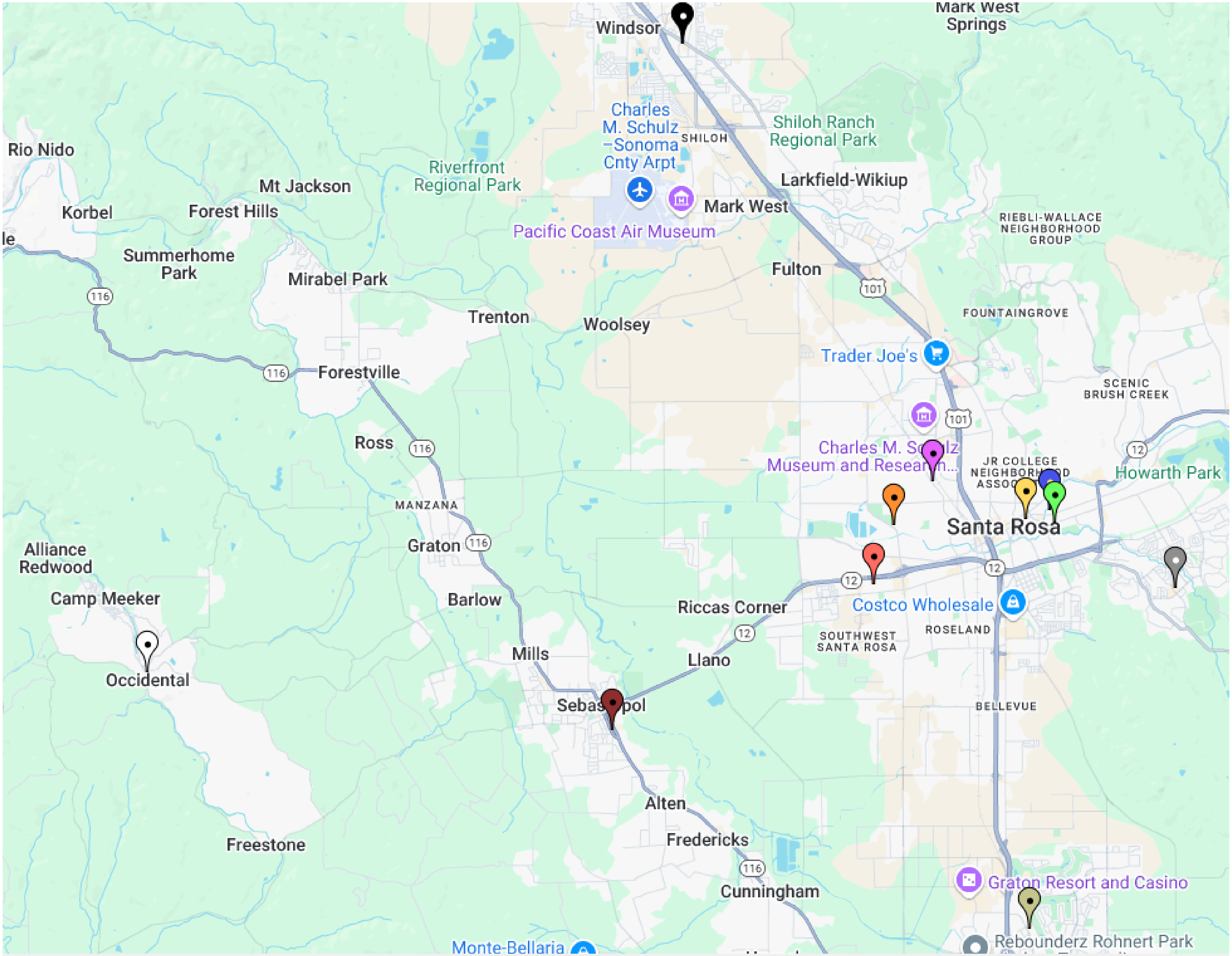

Windsor
Mark West Springs
Charles M. Schulz –Sonoma Cnty Arpt
SHILOH
Shiloh Ranch Regional Park
Rio Nido
Mt Jackson
Riverfront Regional Park
Larkfield-Wikiup
Korbel
Forest Hills
Mark West
Pacific Coast Air Museum
RIEBLI-WALLACE NEIGHBORHOOD GROUP
Summerhome Park
Mirabel Park
Fulton
101
116
FOUNTAINGROVE
Trenton
Woolsey
Trader Joe's
Forestville
SCENIC BRUSH CREEK
Ross
Charles M. Schulz Museum and Research...
JR COLLEGE NEIGHBORHOOD ASSOC
Howarth Park
MANZANA
Santa Rosa
Graton
Alliance Redwood
12
Camp Meeker
Barlow
Riccas Corner
Costco Wholesale
SOUTHWEST SANTA ROSA
ROSELAND
Mills
Llano
Occidental
Sebastopol
BELLEVUE
Alten
Freestone
Fredericks
Graton Resort and Casino
Cunningham
Monte-Bellaria
Rebounderz Rohnert Park
