# Supplementary material for: Optimizing Veteran-Facing Materials for Vending Machine-Dispensed HIV Self-Testing: Formative Qualitative Study
Source: JMIR Form Res. 2026 Jul 23;10:e93868. doi: 10.2196/93868 (PMC13448198; doi:10.2196/93868)
Supplement: Supplementary file 3 [file formative_v10i1e93868_app4.pdf]

UCSF
University of California
San Francisco

Introduction

We are asking you to take part in a paid research study being done by [study PI name] at the University of California, San Francisco, and the Veteran's Affairs Medical Center, San Francisco. Being in this study is optional. If you choose to be in the study, you will complete a survey. You will be paid $20 via an electronic VISA gift card. You may respond to the survey as many times as you'd like, but will only receive up to $40 in total compensation for your responses.

We will ask questions related to your demographics, previous HIV testing and results, HIV self-testing, access to HIV testing, experience using HIV self-tests, sexual history, behaviors that increase the chance of getting HIV, and drug use. This survey will help us learn more about the use of vending machines to dispense Human Immunodeficiency Virus (HIV) self-tests and their impact on expanding

access to Veterans. The survey will take about 20 minutes to complete.

You can skip questions that you do not want to answer by selecting the "prefer not to answer" option or stop the survey at any time. We will keep your answers confidential and will not share your personal information with anyone outside the research team.

Questions? Please contact [study PI name, email, and phone number]. If you have questions or concerns about your rights as a research participant, you can call the UCSF Institutional Review Board at [phone number].

If you want to participate in this study, select Agree below to start the survey. If you do not want to participate, you may close the browser window.

- ○ Agree
- ○ Disagree

Decline

Thank you for your interest. Based on your previous response, you have declined to participate. You may close your browser window.

Consent_Agree

How **many** HIV self-tests have you accessed from the vending machine? Drag the slider bar to record your response.

1 2 3 4 5 6 7 8 9 10 11 12 13 14 15 16 17 18 19 20

What do you **plan to do** with the most recent HIV self-test?

- I already used it to test myself for HIV
- I will use it to test myself for HIV in the future
- I will give/gave it to a sexual partner to test for HIV
- I will give/gave it to a friend, family member, or someone else I know to test for HIV
- Other, please describe:
- Prefer not to answer

Where did you use the HIV self-test?

- ○ At home
- ○ At work
- ○ In a personal vehicle
- ○ [ ] Other, please describe:
- ○ Prefer not to answer

What were your HIV self-test **results**?

- ○ Reactive (two red lines; positive result)
- ○ Not reactive (one red line; negative result)
- ○ Prefer not to answer

Based on your HIV self-test results, what do you plan to do **next**? Select all that apply.

- ☐ Get repeat testing
- ☐ Talk to a provider about HIV treatment
- ☐ Talk to a provider about HIV pre-exposure prophylaxis (HIV PrEP), a medication to prevent HIV through sex or injection drug use

☐ [ ] Other, please describe:

☐ Prefer not to answer

Before having access to the HIV self-test, **when** was your last HIV test?

○ Less than 3 months ago
○ 3-6 months ago
○ >6 months to <1 year ago
○ 1-3 years ago
○ More than 3 years ago
○ I have never been tested for HIV
○ Prefer not to answer

In the past, what **routes** have you been tested for HIV? Select all that apply.

☐ I used an oral swab self-test
☐ I self-administered a finger stick (blood sample) test using a lancet
☐ A healthcare provider administered a finger stick (blood sample) test using a lancet
☐ A healthcare provider drew blood from a vein
☐ [ ] Other, please describe:
☐ Not sure

☐ Prefer not to answer

What is your **most preferred** method for getting tested for HIV?

- ○ I use an oral swab self-test
- ○ I self-administer a finger stick (blood sample) test using a lancet
- ○ A healthcare provider administers a finger stick (blood sample) test using a lancet
- ○ A healthcare provider draws blood from a vein
- ○ [ ] Other, please describe:
- ○ I would not get tested
- ○ Not sure
- ○ Prefer not to answer

If this HIV oral swab self-test **was not available**, how would you get tested for HIV? Select all that apply.

- ☐ I would get tested in a Veterans Affairs setting (e.g., hospital, outpatient clinic)
- ☐ I would get tested in a community setting (e.g., hospital, outpatient clinic)
- ☐ I would purchase a self-test (e.g., in a pharmacy, online store)
- ☐ I would order a free test online (e.g., TakeMeHome)
- ☐ [ ] Other, please describe:

☐ I would not get tested

☐ Not sure

☐ Prefer not to answer

If there was a cost, how much would you be **able to pay** for HIV testing? Drag the slider bar to record your response.

Cost (USD)

0 5 10 15 20 25 30 35 40 45 50 55 60 65 70 75 80 85 90 95 100

How likely are you to **recommend** HIV self-testing to a friend or a colleague?

Very likely	Likely	No Opinion	Unlikely	Very unlikely
○	○	○	○	○

How **comfortable** did you feel accessing the HIV self-test from the vending machine?

Very comfortable	Comfortable	No Opinion	Uncomfortable	Very uncomfortable
○	○	○	○	○

How **confident** did you feel accessing the HIV self-test from the vending machine?

Very confident	Confident	No Opinion	Unconfident	Very unconfident
○	○	○	○	○

How **acceptable** was it accessing the HIV self-test from the vending machine?

Very acceptable	Acceptable	No Opinion	Unacceptable	Very unacceptable
○	○	○	○	○

How much **effort** did it take to access the HIV self-test from the vending machine?

No effort at all	A little effort	No Opinion	A lot of effort	Huge effort
○	○	○	○	○

How **convenient** was accessing the HIV self-test from the vending machine?

Very convenient	Convenient	No opinion	Inconvenient	Very inconvenient
○	○	○	○	○

How **satisfied** were you with was accessing the HIV self-test from the vending machine?

Very satisfied	Satisfied	No opinion	Dissatisfied	Very dissatisfied
○	○	○	○	○

How **private** was accessing the HIV self-test from the vending machine?

	Very private	Private	No Opinion	Not private	Completely not private
	○	○	○	○	○

Please rate your level of **agreement** with the following statement: Accessing the HIV self-test from the vending machine has improved my ability to test for HIV.

	Strongly agree	Agree	No Opinion	Disagree	Strongly disagree
	○	○	○	○	○

What **other types** of self-tests would you recommend adding to the vending machines? Select all that apply.

- ☐ Chlamydia/gonorrhea tests
- ☐ Syphilis tests
- ☐ Pregnancy tests
- ☐ COVID-19 tests
- ☐ Other, please describe
- ☐ None
- ☐ Prefer not to answer

What **barriers** are there to using the HIV self-test? Select all that apply.

- ☐ Difficulty using the test
- ☐ Fearful of the results
- ☐ It interferes with my other health priorities
- ☐ It will impact my mental health
- ☐ Unable to access follow-up testing and treatment
- ☐ [ ] Other, please describe: (free text)
- ☐ None
- ☐ Not sure
- ☐ Prefer not to answer

What is your **age** in years?

[ ]

In research, we often must present demographic information in categories. We understand these labels are limiting. If you had to select one of the options below, which one best describes your **gender identity**?

- ○ Man

- ○ Woman
- ○ Agender
- ○ Gender fluid
- ○ Gender queer
- ○ Gender questioning
- ○ Māhū, or muxe, or two spirit
- ○ Non-binary/ Gender non-conforming
- ○ [ ] Another term best describes my gender:
- ○ Prefer not to answer

Do you identify as **transgender**?

- ○ Yes
- ○ No
- ○ Prefer not to answer

What labels best describe your **race/ethnicity**? Select all that apply.

- ☐ American Indian, Native American, or Alaska Native
- ☐ Arab, Middle Eastern, or North African
- ☐ Asian or Asian American
- ☐ Black or African American
- ☐ Hispanic, Latinx, Latine, Latino, or Latina

- ☐ Native Hawaiian or Other Pacific Islander
- ☐ White or European American
- ☐ [   ] Some other race, ethnicity, or origin:
- ☐ Don't know
- ☐ Prefer not to answer

What term best describes your **sexual orientation**?

- ○ Asexual or aromantic
- ○ Bisexual
- ○ Demisexual
- ○ Gay
- ○ Lesbian
- ○ Pansexual
- ○ Queer
- ○ Questioning
- ○ Sexually fluid
- ○ Straight or Heterosexual
- ○ [   ] Another term best describes my sexual orientation:
- ○ Prefer not to answer

Where have you **lived** in the past 6 months? Select all that

apply.

- ☐ Apartment, house, or room
- ☐ With friend or family (e.g., couch-surfing)
- ☐ Motel or hotel
- ☐ Long-term institution (e.g., nursing home)
- ☐ Short-term institution (e.g., hospital, rehabilitation center, drug treatment center)
- ☐ Homeless shelter
- ☐ Outside (e.g., street, beach, park, vehicle, abandoned building)
- ☐ Other: ____________
- ☐ Prefer not to answer

I **currently live** in an area best described as:

- ○ Rural
- ○ Suburban
- ○ Urban
- ○ Prefer not to answer

Have you ever served on **active duty** in the US Armed Forces, Reserves, or National Guard?

- ○ On active duty in the past, but not now
- ○ Currently on active duty

- ○ Only on active duty for training in the Reserves or National Guard
- ○ Never served in the military
- ○ Prefer not to answer

Which branch(es) did you serve? Select all that apply.

- ☐ Air Force
- ☐ Army
- ☐ Coast Guard
- ☐ Marine Corps
- ☐ Navy
- ☐ National Guard
- ☐ Reserves
- ☐ Space Force
- ☐ Prefer not to answer

What is your **service-connected disability** rating?

- ○ 0%
- ○ 10-20%
- ○ 30-40%
- ○ 50-60%
- ○ 70% or higher
- ○ Not sure
- ○ Prefer not to answer

Thinking about the **past 6 six months**, do any of the following apply to you? Select all that apply.

- ☐ I had anal/vaginal sex with someone who has HIV
- ☐ I had anal/vaginal sex with someone and do not know if they have HIV
- ☐ I had anal/vaginal sex without a condom
- ☐ I had a sexually transmitted infection (e.g., chlamydia, gonorrhea, syphilis)
- ☐ I exchanged sex for money, drugs, food, or a place to stay
- ☐ I had sex with someone who injected substances (e.g., heroin, fentanyl, cocaine, methamphetamine)
- ☐ I had sex while using alcohol or drugs (e.g., heroin, fentanyl, cocaine, methamphetamine)
- ☐ I Injected substances (e.g., heroin, fentanyl, cocaine, methamphetamine)
- ☐ I shared needles/injection equipment with another person
- ☐ None of the above
- ☐ Prefer not to answer

Before today, have you **heard of** any of the following medications? Select all that apply.

- ☐ HIV PrEP, a medication to prevent HIV through sex or injection drug use
- ☐ HIV PEP, a medication that can be taken within 3 days of possible exposure to HIV to prevent infection
- ☐ DoxyPEP, a medication that can be taken by men who have sex with men and transgender women to prevent chlamydia, gonorrhea, and syphilis

- [ ] None of the above
- [ ] Prefer not to answer

Would you be **willing** to take any of the following medications if offered/available to you? Select all that apply.

- [ ] HIV PrEP, a medication to prevent HIV through sex or injection drug use
- [ ] HIV PEP, a medication that can be taken within 3 days of possible exposure to HIV to prevent infection
- [ ] DoxyPEP, a medication that can be taken by men who have sex with men and transgender women to prevent chlamydia, gonorrhea, and syphilis
- [ ] None of the above
- [ ] Prefer not to answer

Have you **taken** any of the following medications in the **past year**? Select all that apply.

- [ ] HIV PrEP, a medication to prevent HIV through sex or injection drug use
- [ ] HIV PEP, a medication that can be taken within 3 days of possible exposure to HIV through sex or injection drug use
- [ ] DoxyPEP, a medication that can be taken by men who have sex with men and transgender women to prevent chlamydia, gonorrhea, and syphilis
- [ ] None of the above
- [ ] Prefer not to answer

Powered by Qualtrics
