# Supplementary material for: Optimizing Veteran-Facing Materials for Vending Machine-Dispensed HIV Self-Testing: Formative Qualitative Study
Source: JMIR Form Res. 2026 Jul 23;10:e93868. doi: 10.2196/93868 (PMC13448198; doi:10.2196/93868)
Supplement: Supplementary file 4 [file formative_v10i1e93868_app5.pdf]

Qualitative Interview Guide/Script

Introduction:

Hello, my name is ___. I am a researcher/pharmacist/pharmacy student/___ from the University of California, San Francisco (UCSF), and San Francisco VA. We are conducting a paid research study evaluating experiences and perspectives of Veterans who accessed and used a HIV self-test dispensed via harm reduction vending machines.

Interviewer: *Obtain informed consent for interview and recording.*

Before we proceed, I would like to review the informed consent with you. Your participation is voluntary, and your responses will be kept confidential. This interview will last for approximately 60 minutes. With your consent, we will do an interview that is digitally audio-recorded, and you will complete a brief web-based demographics survey. Your name and identifying information will not be associated with your responses. Please review this consent form, and let me know if you have any questions, concerns, or would like a copy of the form.

Experiences with HIV Testing:

1. What comes to mind when you think about HIV testing?
    - Probe: words, phrases, images
2. What has been your experience testing for HIV in the past?
    - Prompt: Did you go to a medical provider, VA clinic, or public health clinic? Was it a blood draw or finger stick test? Was it a good or bad experience, and why?

Knowledge of Self-Testing:

3. Before accessing the HIV self-test from the vending machine, what had you heard about HIV self-testing?
    - Prompt: How did you hear about it, for example, an advertisement on TV or in a store?

Interest Accessing via Vending Machine:

4. What made you interested in accessing a HIV self-test from the vending machine?
    - Probe: to try something new, to test yourself for HIV, to give to someone else, it was free

Experience Accessing via Vending Machine:

5. What was your experience like accessing a HIV self-test from the vending machine?
    - Prompt: Was it quick an easy? Did you experience any challenges using the vending machine?

Concerns Accessing via Vending Machine:

6. Did you have any concerns about privacy when accessing the HIV self-test from the vending machine?
    - Prompt: What specific concerns? How could we address those concerns?

7. Did you have any concerns about stigma when accessing the HIV self-test from the vending machine?
    - <u>Prompt</u>: What specific concerns? How could we address those concerns?

<u>Experience Self-Testing</u>:

8. What was your experience like testing yourself for HIV?
    - <u>Prompt</u>: Did you test at home? Were you alone or with someone else?

<u>Ease of Self-Test Use</u>:

9. How easy or difficult to use was the HIV self-test?
    - <u>Prompt</u>: What made it easy or difficult to use? How could it be made easier?

<u>Motivations for Self-Testing</u>:

10. What motivated you to use the HIV self-test?
    - <u>Probe</u>: to try something new, to test yourself for HIV, it was free

<u>Willingness to Pay for Self-Testing</u>:

11. If the HIV self-test was not free, how much would you be willing to pay?
    - <u>Probe</u>: $10, $20, $30

<u>Preferences for Testing</u>:

12. Do you prefer testing yourself for HIV at home over getting tested in other settings, like at a VA or public health clinic?
    - <u>Probe</u>: why

<u>Concerns for Self-Testing</u>:

13. What concerns did you have when testing yourself for HIV?
    - <u>Probe</u>: worry about testing accuracy, knowing the results, or having HIV; unsure of what to do if results were positive, where or how to get HIV treatment

<u>Support Needs</u>:

14. What support would you find most helpful before using a HIV self-test?
    - <u>Probe</u>: a dedicated person to call, specific educational materials, video instructions

<u>Follow-up</u>:

15. What did you do after using the HIV self-test?
    - <u>Prompt</u>: Did you seek additional testing to confirm the results? Did you contact a medical provider? Did you contact a friend or family member for support?
16. What would motivate you to seek additional testing to confirm the HIV self-test results?
    - <u>Prompt</u>: if results were positive, to confirm you properly performed the test, if additional testing was easy to access, if it was free

Recommendations for Other Self-Test Distribution via Vending Machines:

17. What other types of self-tests would you like added to the vending machines?
    - Probe: pregnancy, COVID-19, syphilis, chlamydia/gonorrhea

Interviewer:

That concludes our interview. Is there anything else you would like to share with the research team at this time?

Thank you so much for your participation. As a reminder, your responses are confidential, and your insights are valuable to our research. If you have any questions, feel free to ask. Thank you again.
