# Supplementary material for: Optimizing Veteran-Facing Materials for Vending Machine-Dispensed HIV Self-Testing: Formative Qualitative Study
Source: JMIR Form Res. 2026 Jul 23;10:e93868. doi: 10.2196/93868 (PMC13448198; doi:10.2196/93868)
Supplement: Supplementary file 5 [file formative_v10i1e93868_app8.pdf]

STUDY VOLUNTEERS NEEDED

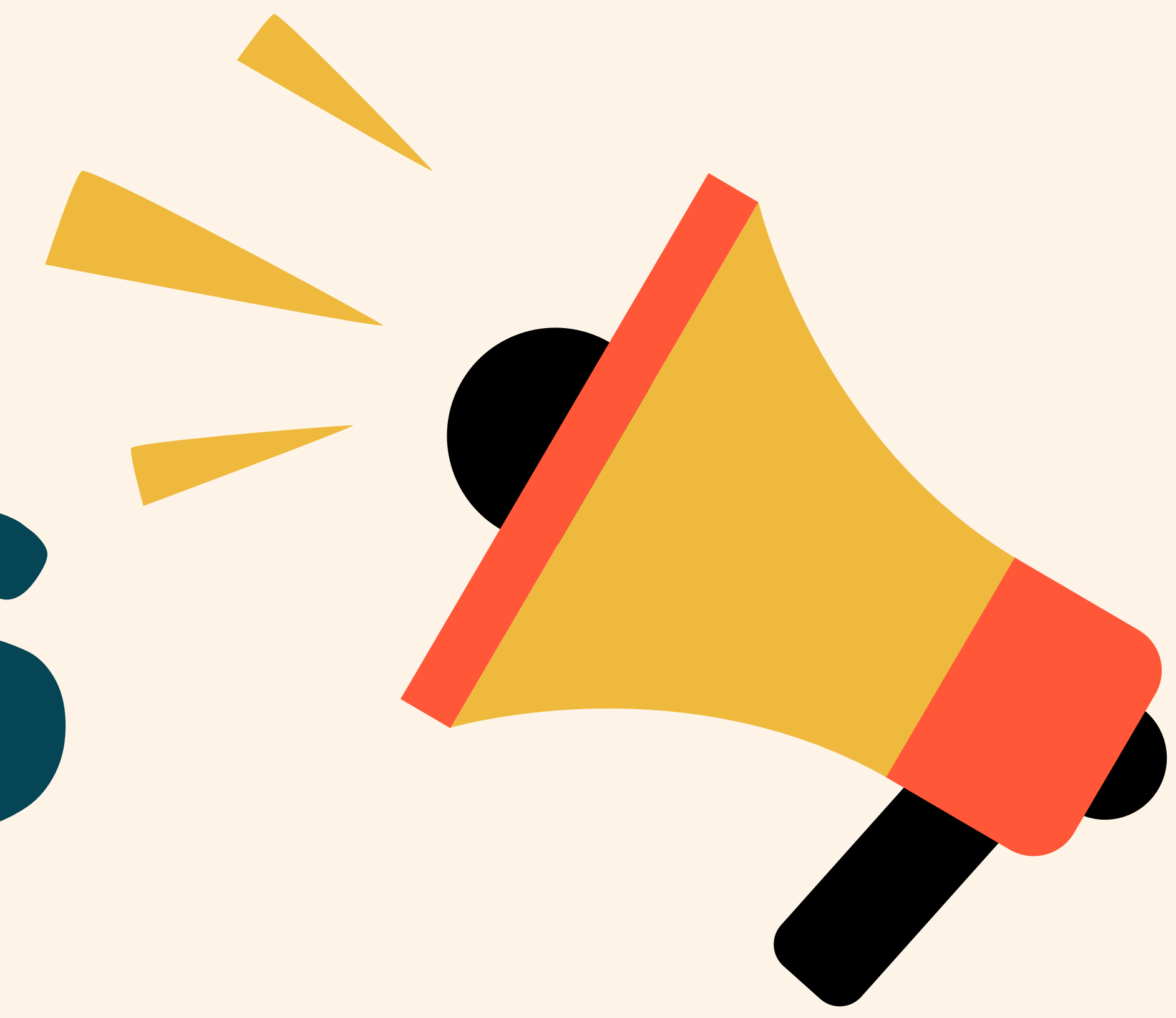

This research study aims to learn about Veteran interest and experience using vending machines to access and use HIV self-tests.

WHO IS ELIGIBLE

- All participants must be Veterans.

WHAT IS REQUIRED

- All participants will access a human immunodeficiency virus (HIV) self-test from the harm reduction vending machine.
- Participants may complete a survey and/or an optional interview.
- Survey participants will complete a confidential online survey about their experience accessing an HIV self-test from the vending machine.
- Interview participants will complete a 1-hour interview about their experience using the HIV self-test.

GETTING STARTED

- Survey participants scan the QR code on the HIV self-test to get started.
- Interview participants contact [study PI, phone, email].

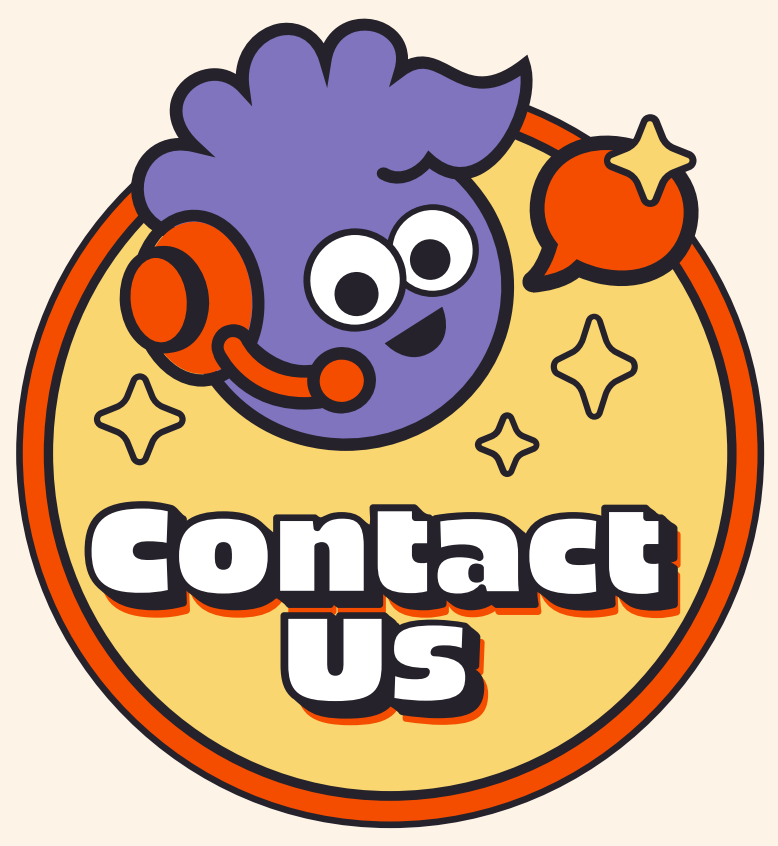

SCAN ME!

PAYMENT

- Survey participants will receive a $20 electronic gift card (max 2 gift cards).
- Interview participants will receive a $100 gift card.

VA | U.S. Department of Veterans Affairs
Veterans Health Administration
San Francisco VA Health Care System
UCSF
