# Supplementary material for: Optimizing Veteran-Facing Materials for Vending Machine-Dispensed HIV Self-Testing: Formative Qualitative Study
Source: JMIR Form Res. 2026 Jul 23;10:e93868. doi: 10.2196/93868 (PMC13448198; doi:10.2196/93868)

HIV 101

Without treatment, HIV (human immunodeficiency virus) can make a person very sick and even cause death. Learning the basics about HIV can keep you healthy and prevent transmission.

HIV CAN BE TRANSMITTED BY

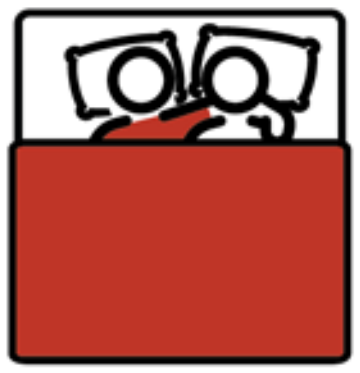

Sexual Contact

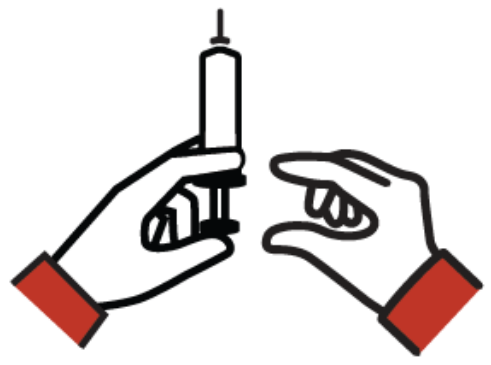

Sharing Needles to Inject Drugs

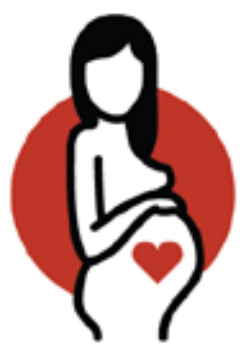

During Pregnancy, Birth, or Breast/Chestfeeding

HIV IS NOT TRANSMITTED BY

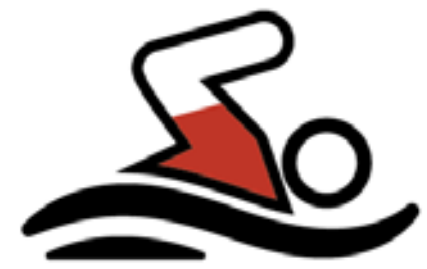

Air or Water

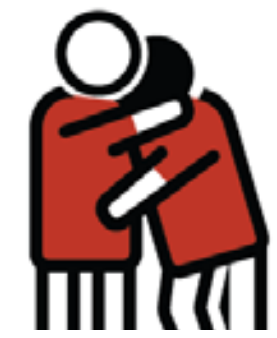

Saliva, Sweat, Tears, or Closed-Mouth Kissing

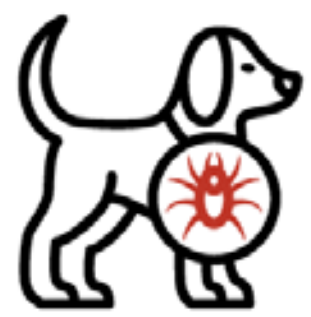

Insects or Pets

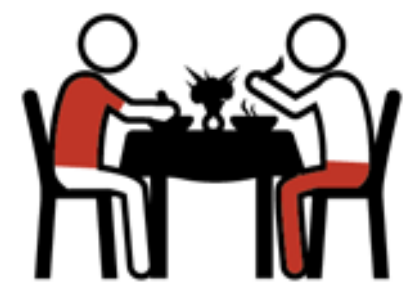

Sharing Toilets, Food, or Drinks

PROTECT YOURSELF FROM HIV

- Get tested at least once or more often if you have certain risk factors.
- Use condoms the right way every time you have anal or vaginal sex.
- Choose activities with little to no risk like oral sex.
- Don't inject drugs, or if you do, don't share needles, syringes, or other drug injection equipment.

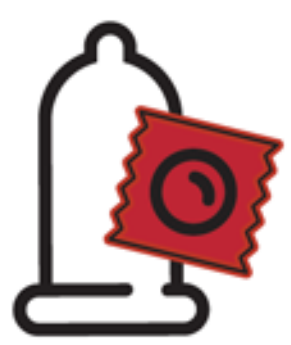

- If you engage in behaviors that may increase your chances of getting HIV, ask your health care provider if pre-exposure prophylaxis (PrEP) is right for you.

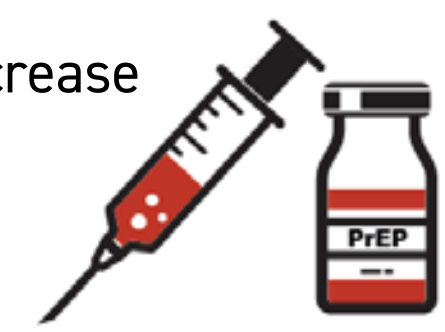

- If you think you've been exposed to HIV within the last 3 days, ask a health care provider about post-exposure prophylaxis (PEP) right away. PEP can prevent HIV, but it must be started within 72 hours.
- Get tested and treated for other STDs.

KEEP YOURSELF HEALTHY AND PROTECT OTHERS IF YOU HAVE HIV

- Find HIV care and stay in HIV care.
- Take your HIV treatment as prescribed.
- Get and keep an undetectable viral load. This is the best way to stay healthy and protect others.

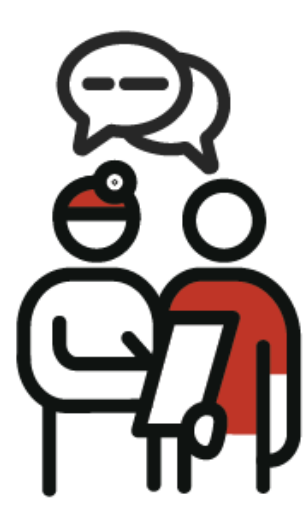

- If you have an undetectable viral load, you will not transmit HIV through sex.
- If your viral load is not undetectable—or does not stay undetectable—you can still protect your partners by using other HIV prevention options.

Scan to learn more!

LET'S STOP HIV TOGETHER™

Ending the HIV Epidemic

For more information, please visit **www.cdc.gov/hiv.**

INFORMACIÓN BÁSICA SOBRE EL VIH

Sin tratamiento, el VIH (virus de la inmunodeficiencia humana) puede hacer que una persona esté muy enferma, e incluso causarle la muerte. Aprender lo básico sobre el VIH puede mantenerlo saludable y prevenir la transmisión de este virus.

EL VIH PUEDE SER TRANSMITIDO

- Mediante el contacto sexual
- Al compartir las agujas para inyectarse drogas
- Durante el embarazo, el parto o el pecho/lactancia materna

EL VIH NO SE TRANSMITE

- A través del aire o del agua
- Mediante la saliva, el sudor, las lágrimas o los besos con la boca cerrada
- Por los insectos o por las mascotas
- Al compartir el inodoro, los alimentos o las bebidas

PROTÉJASE DEL VIH

- Hágase la prueba al menos una vez o con más frecuencia si tiene ciertos factores de riesgo.
- Use condones de la manera correcta cada vez que tenga relaciones sexuales anales o vaginales.
- Elija actividades que impliquen poco o nada de riesgo, como las relaciones sexuales orales.
- No se inyecte drogas, pero si lo hace, no comparta las agujas, jeringas, u otro equipo de inyección de drogas.
- Si tiene comportamientos que pueden aumentar sus posibilidades de contraer el VIH, pregúntele a su proveedor de atenció médica si la profilaxis preexposicíon (PrEP) es adecuada para usted.
- Si cree que se ha expuesto al VIH dentro delos últimos 3 días, pregúntele de inmediato a un proveedor de atención médica acerca de la profilaxis posexposición (PEP). La PEP puede prevenir el VIH, pero debe comenzarse dentro de las 72 horas de la posible exposición.
- Hágase las pruebas de detección de otras ETS y reciba el tratamiento necesario.

SI TIENE EL VIH, MANTÉNGASE SALUDABLE Y PROTEJA A LOS DEMÁS

- Busque atención médica para el VIH y no deje de recibir la atención médica para el VIH.
- Tomar el tratamiento para el VIH según las indicaciones.
- Obtenga y mantenga una carga viral indetectable. Esta es la mejor manera de mantenerse saludable y proteger a los demás.
- Si tiene una carga viral indetectable, no transmitirá el VIH a su pareja sexual.
- Si su carga viral no es indetectable, o no permanece indetectable, aún puede proteger a sus parejas utilizando otras opciones de prevención del VIH.

¡Escanea para obtener más información!

DETENGAMOS JUNTOS EL VIH™

Ending the HIV Epidemic

How to Use the OraQuick HIV Self-Test

1. Open the Test Tube pouch.
Remove the Test Tube lid.
Insert the Test Tube into the stand.

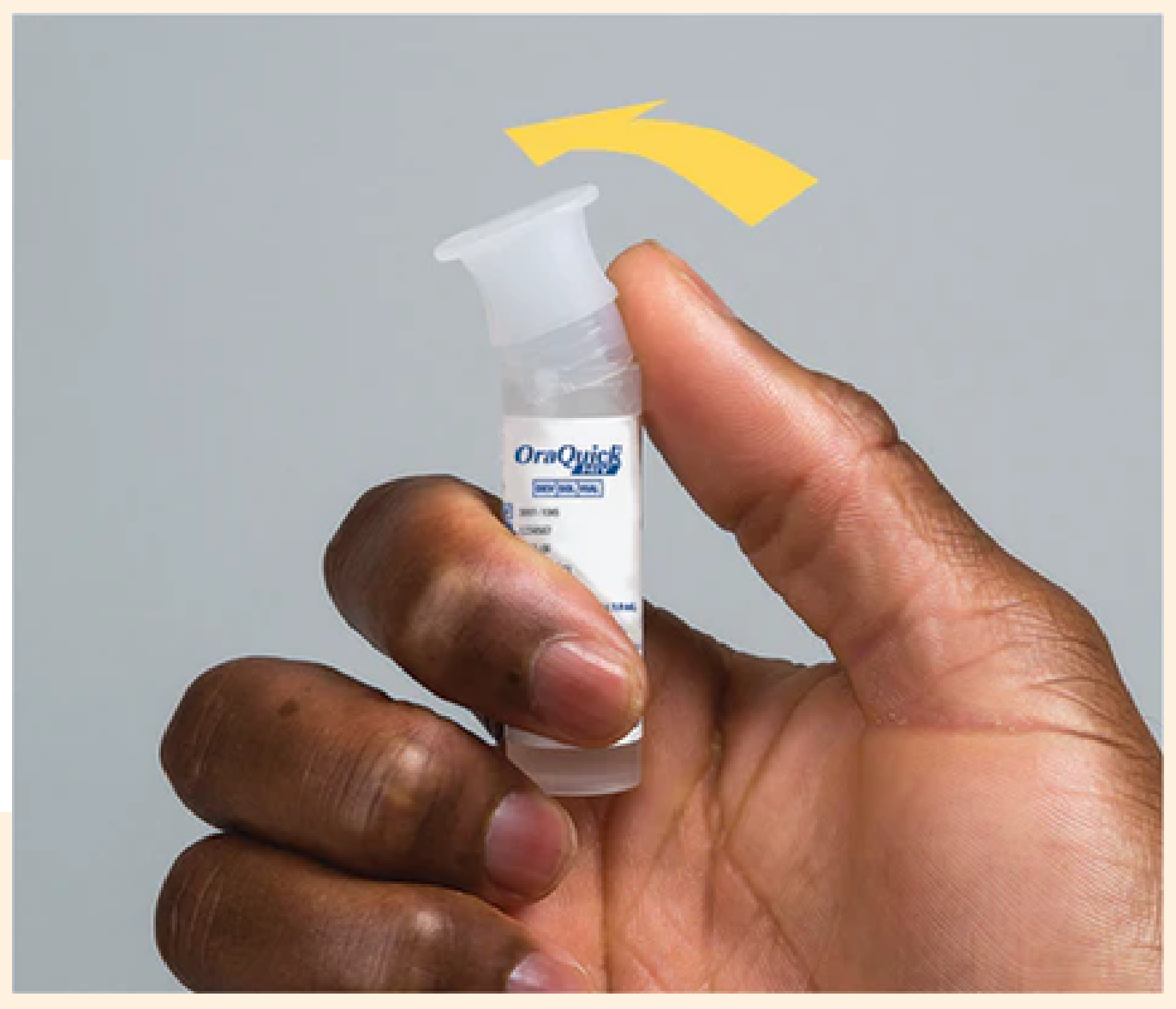

2. Open the Test Stick pouch.
Hold onto the plastic end.
Avoid touching the flat pad.

3. Press the flat pad of the Test Stick on your upper gums.
Swab in one direction.

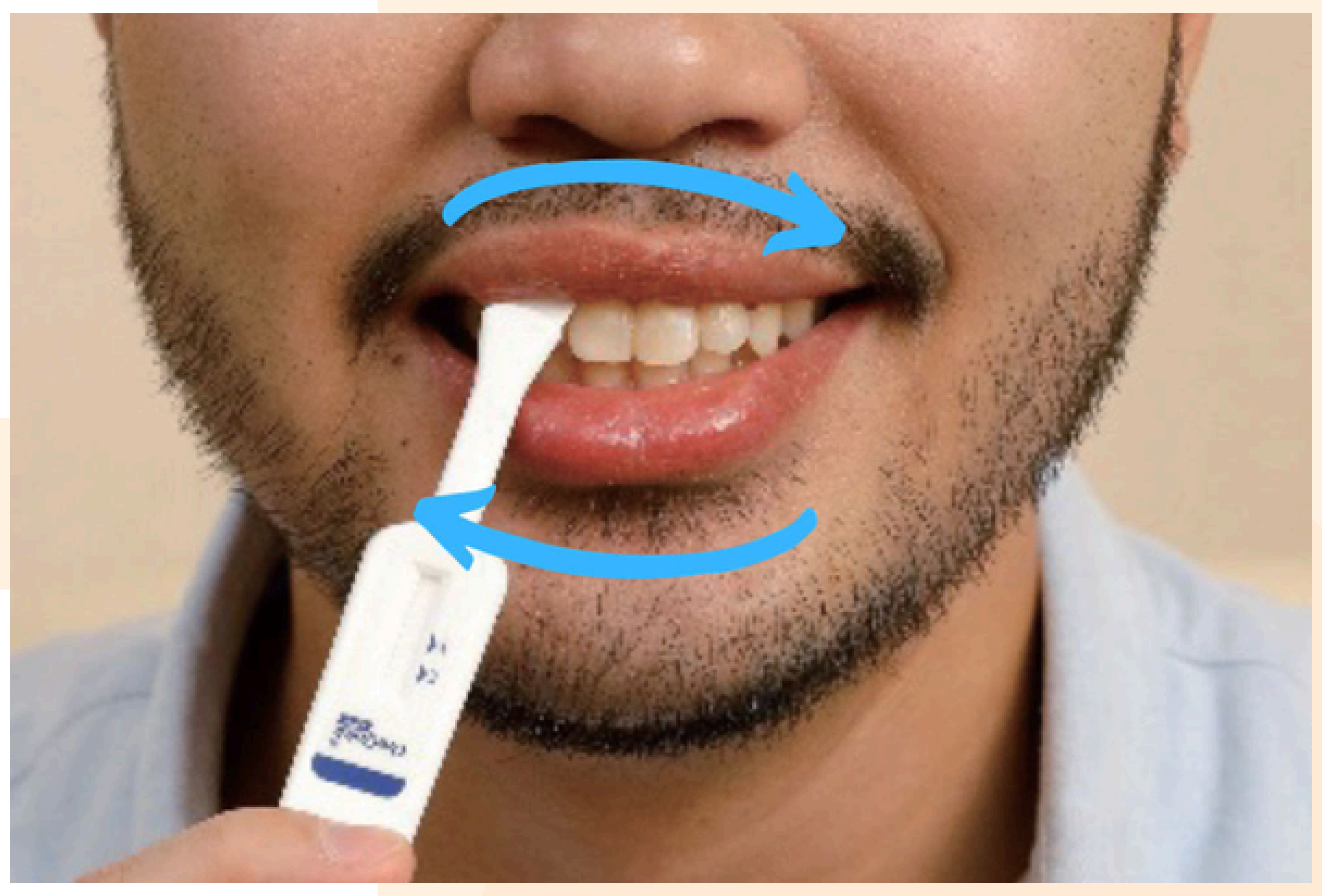

4. Press the flat pad of the Test Stick on your lower gums.
Swab in one direction.

5. Put the Test Stick flat pad in the Test Tube.
Set a timer for 20 minutes.
Read results between 20-40 minutes.

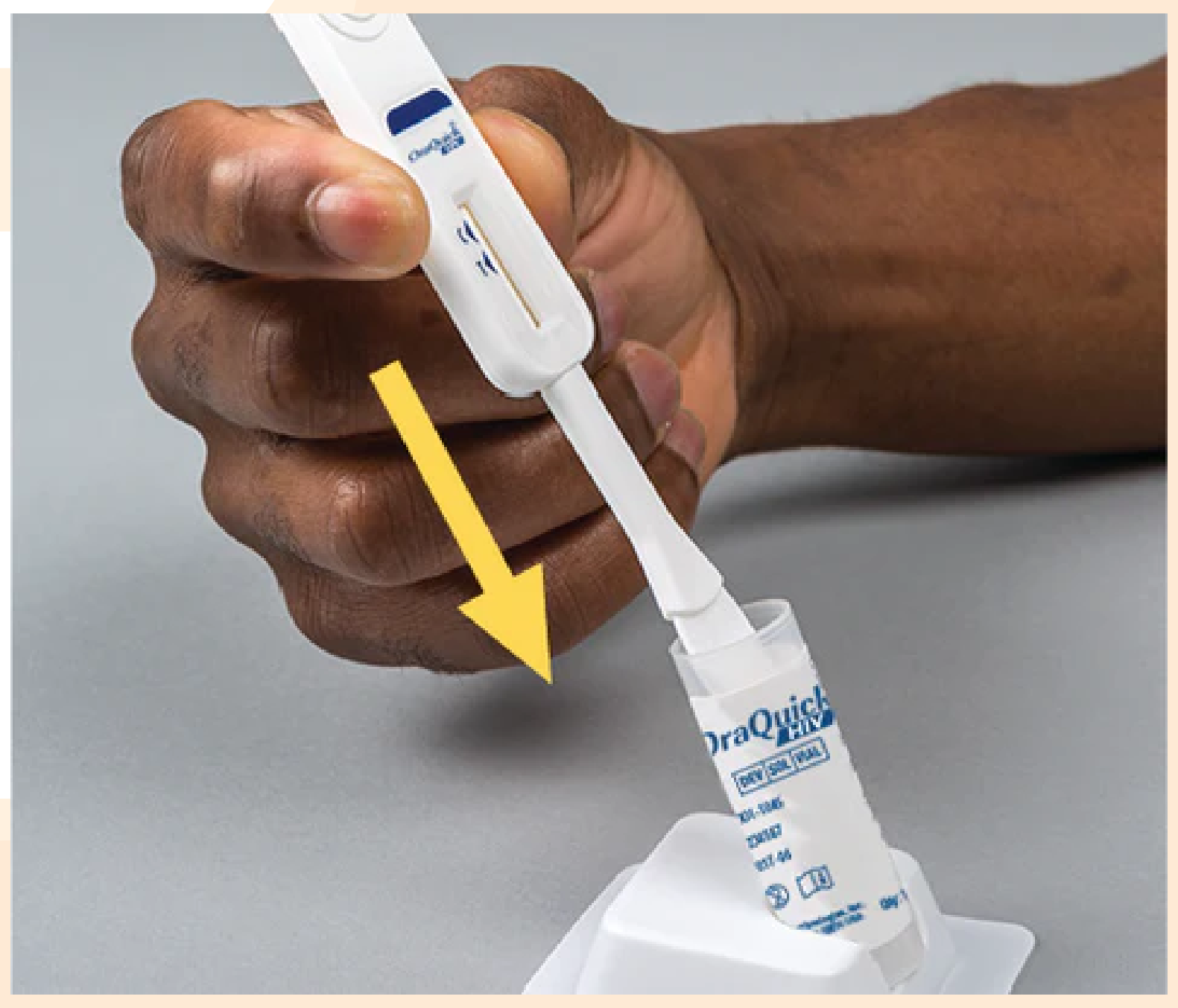

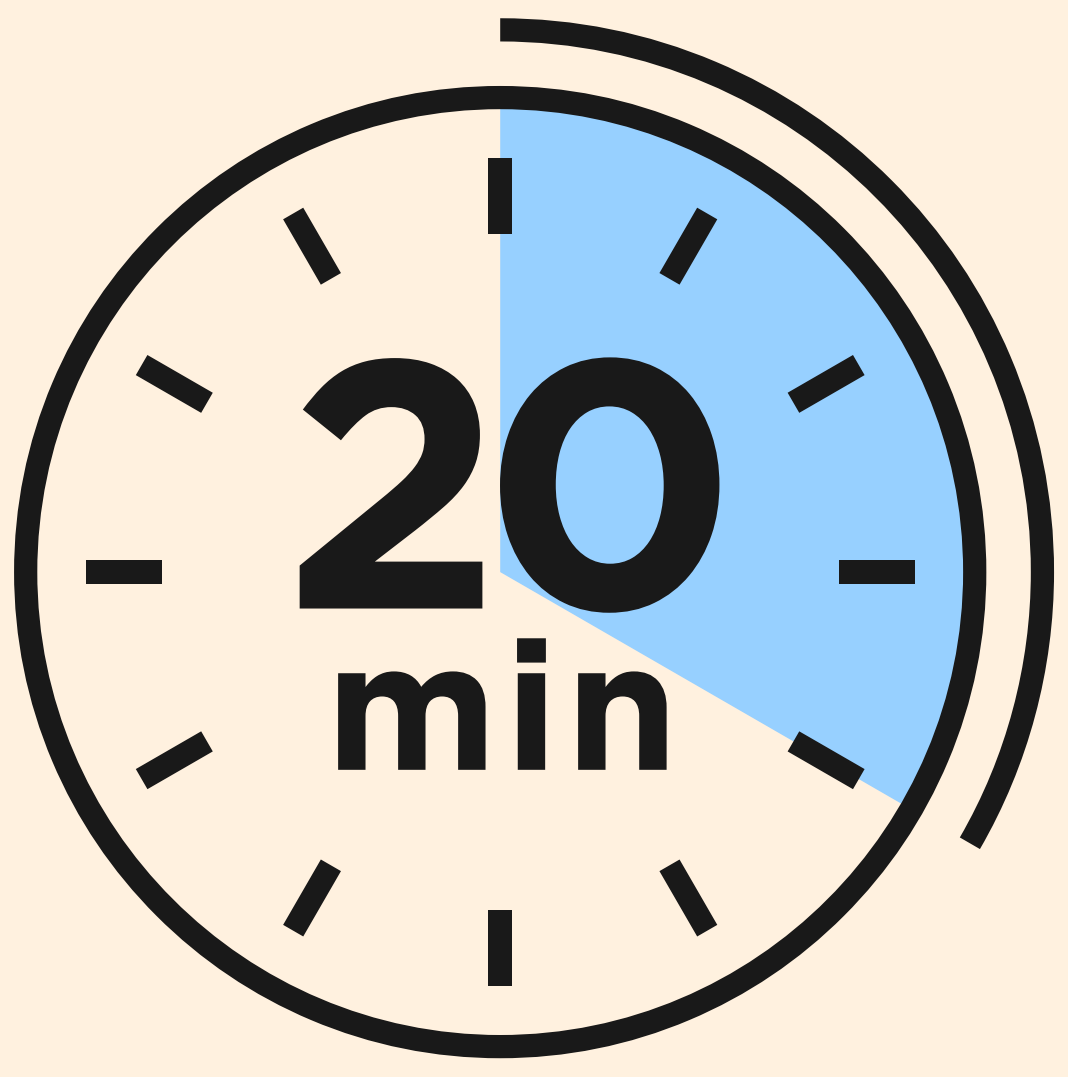

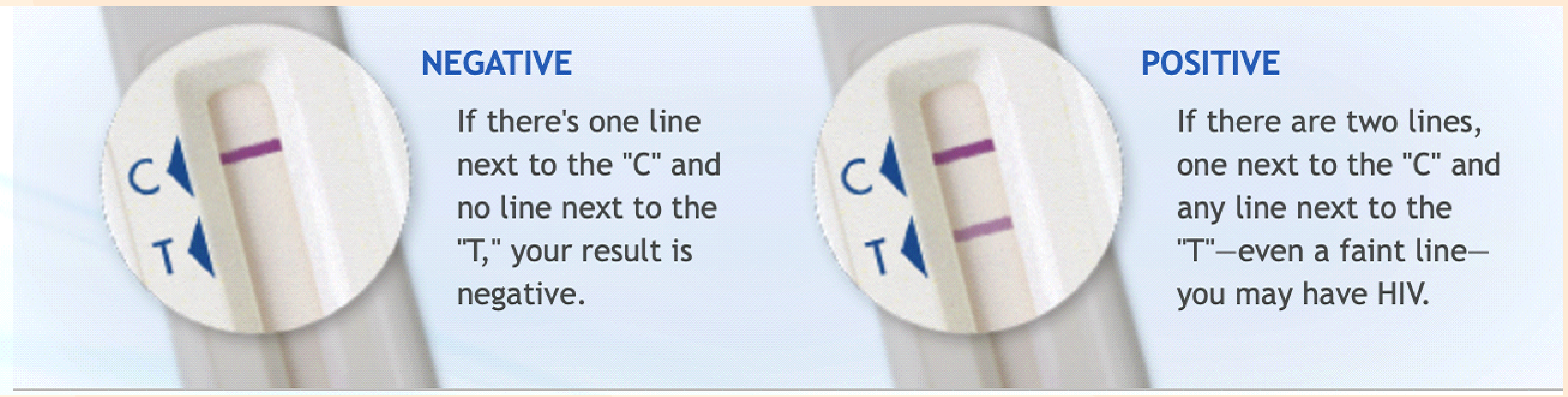

See inside box for detailed instructions.
Scan QR code for video instructions or access here: https://youtu.be/X9LZwswsF80

SCAN ME !

Understanding Your HIV Test Results and Seeking Support

What does a NEGATIVE result mean?

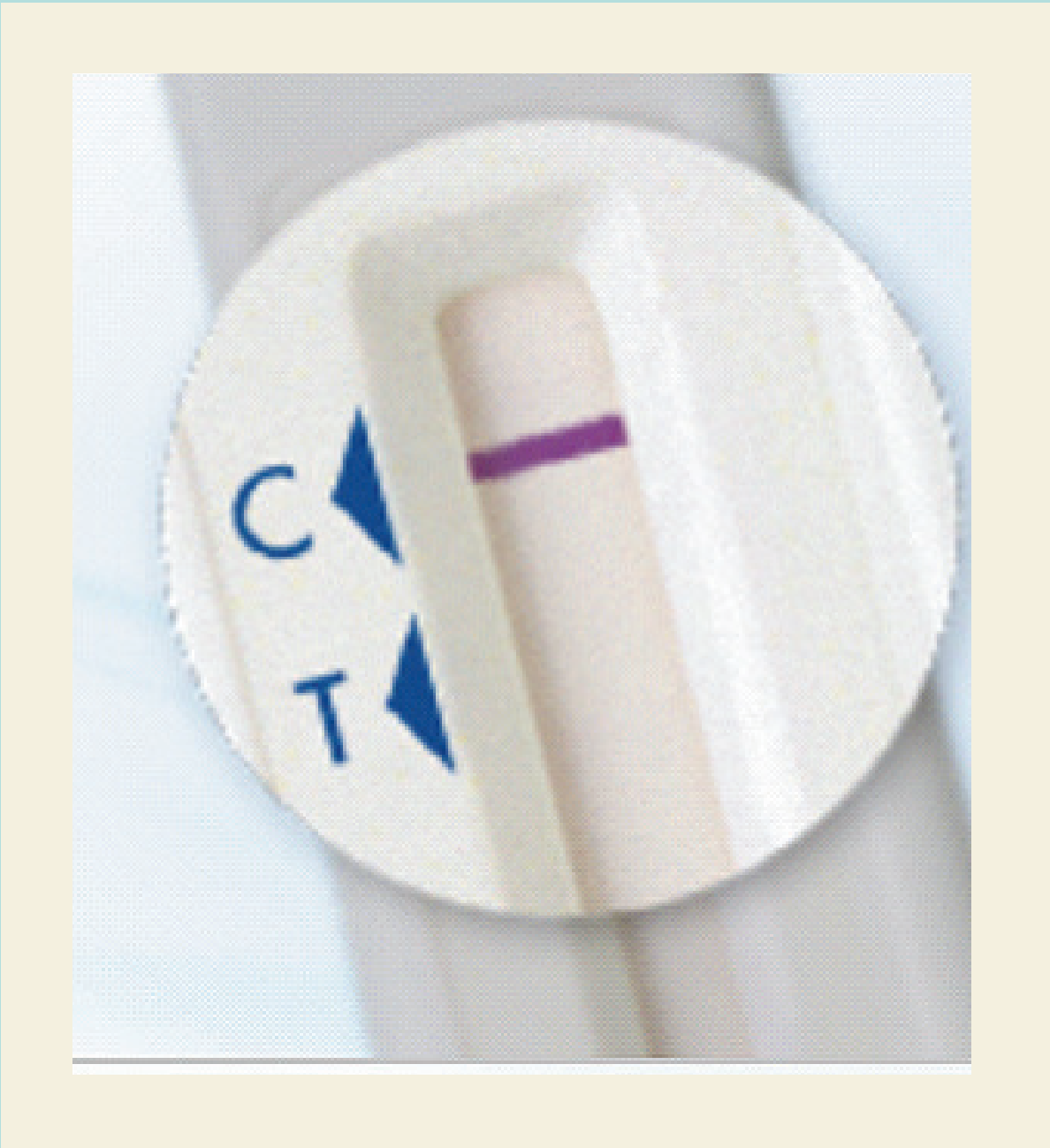

HIV antibodies were not found. This could mean:

- You do not have HIV, or
- You may have HIV, but it was not detected. This is a false-negative result.

It can take 3 months for your body to produce enough HIV antibodies to be detected. If you may have been exposed to HIV in the past 3 months, repeat the test in a few weeks.

What can I do to prevent HIV?

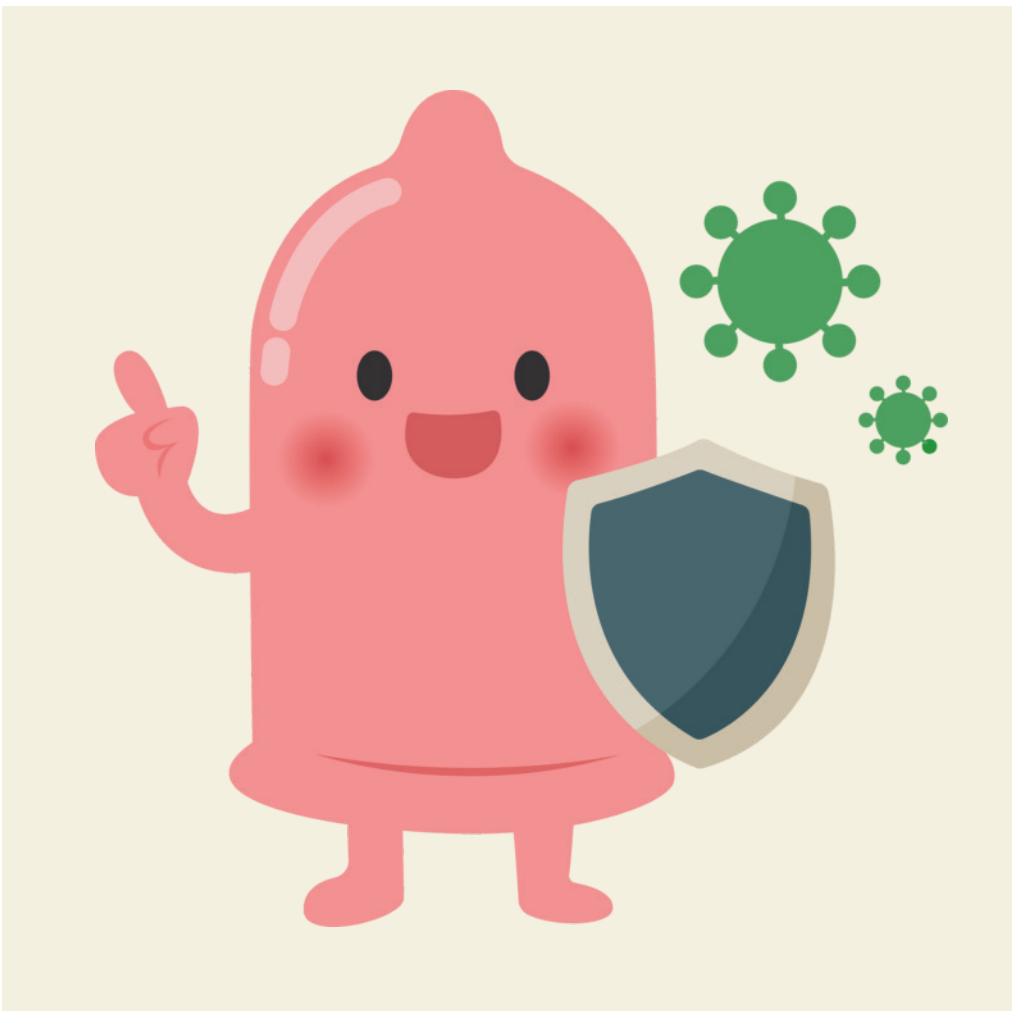

- Use condoms to prevent HIV, viral hepatitis, and other sexually transmitted infections.
- Use water or silicon-based lubricant to prevent condom breakage.
- Take PrEP (pre-exposure prophylaxis), a medication that can prevent you from getting HIV if you are exposed to the virus through sex or injection drug use.
- Oral PrEP can be taken daily or on demand around the time of sex.
- Injectable PrEP can be taken every 2 months or every 6 months.

What does a POSITIVE result mean?

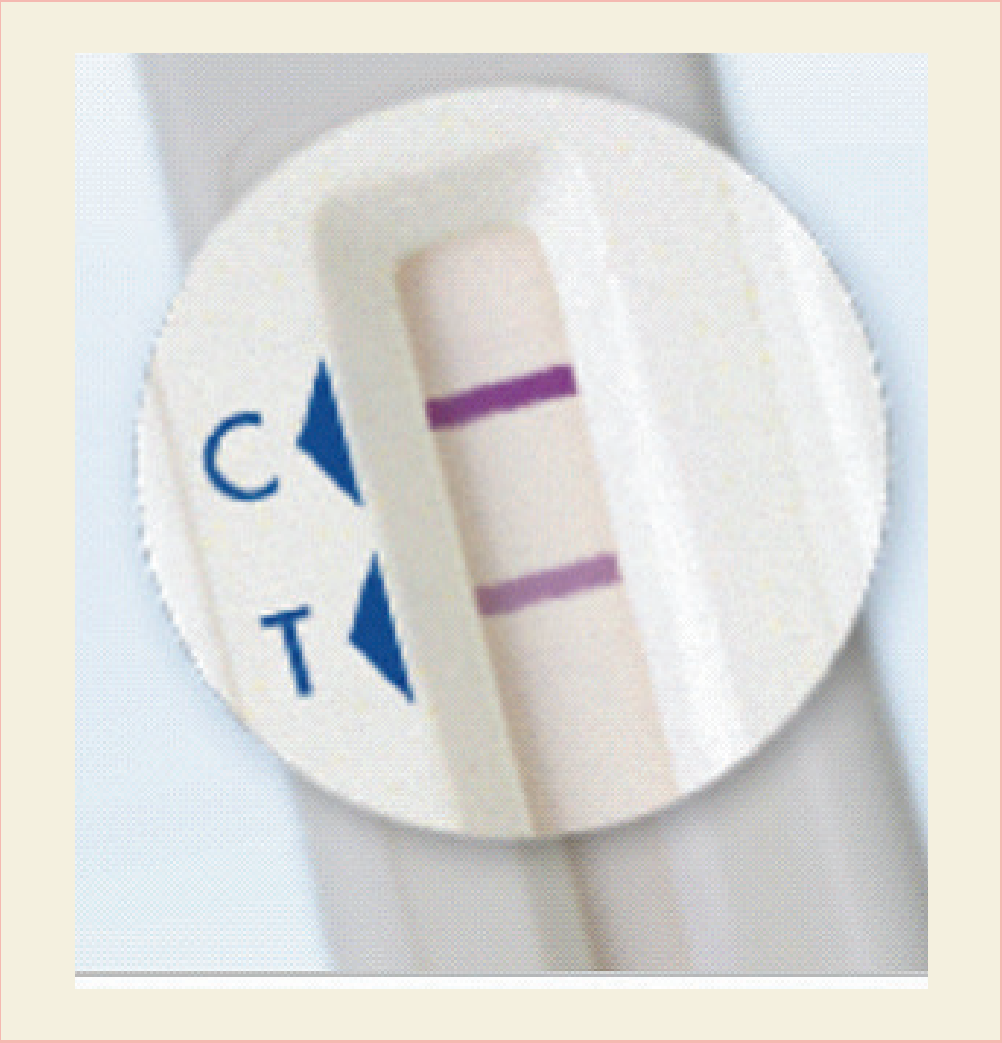

HIV antibodies may be in your body. Positive results must be confirmed by doing a second test for HIV in your blood. On rare occasions the test can give a false-positive result.

What should I do after a POSITIVE test?

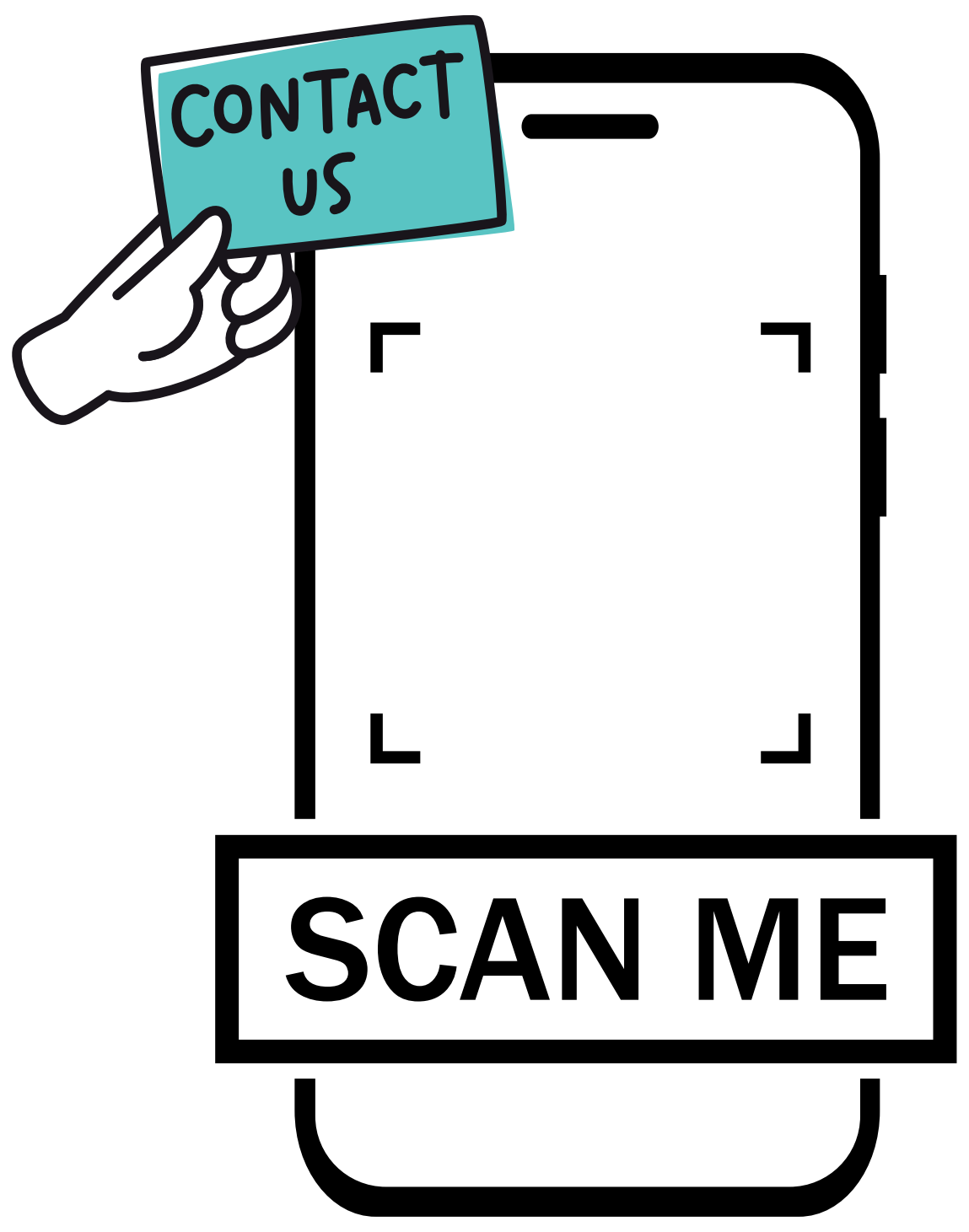

Contact your VA primary care provider or **pharmacist [study PI and phone]** to schedule follow-up testing.

IfyouhaveHIV,youshouldtake**medicine to treat HIV**assoonaspossibleto improveyourhealthandpreventtransmittingHIVtootherpeople.

HIV medicine can reduce the amount of HIV in your blood (called viral load) to a level so low that a test can't detect it. This is known as an "**undetectable viral load**" or "**undetectable**".

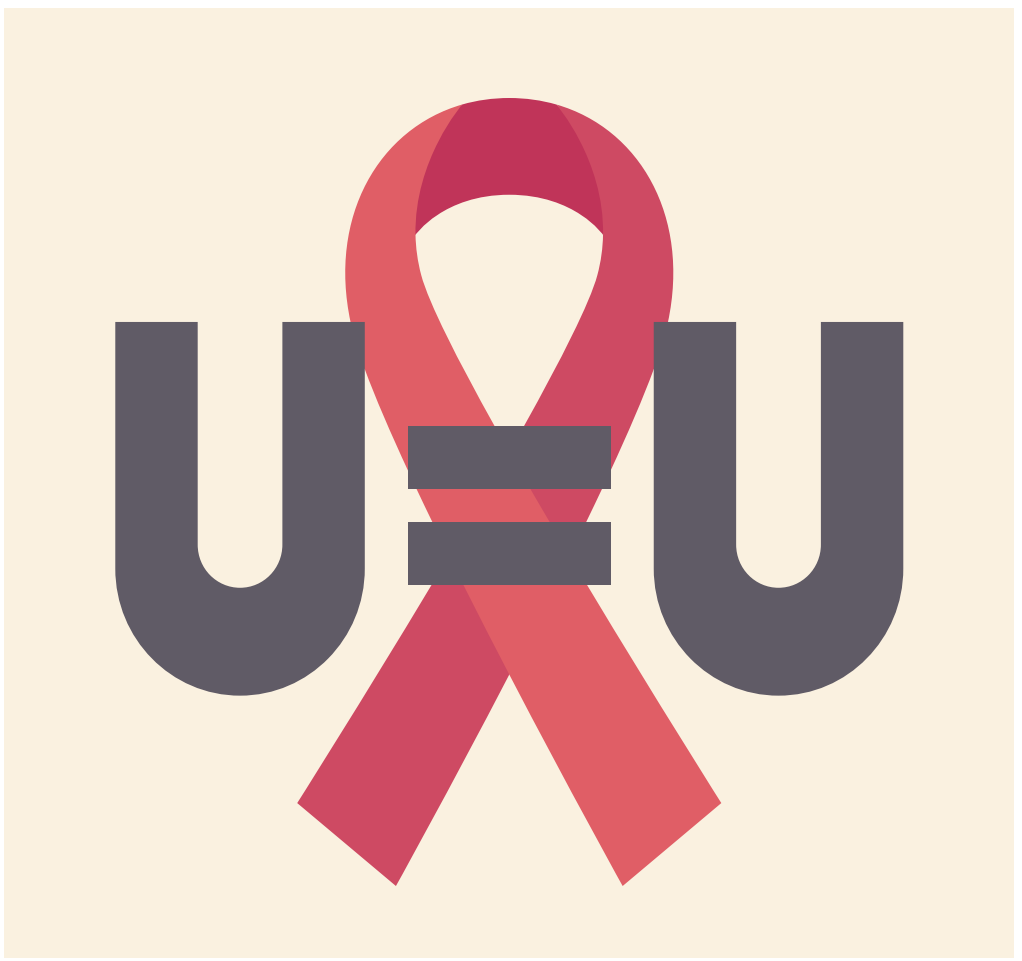

If you get and keep an undetectable viral load, you will not transmit HIV through sex. **Undetectable = Untransmittable (U = U).**

Most people can get an undetectable viral load **within 6 months of treatment.**

Understanding Your HIV Test Results and Seeking Support

Need help, information, or resources?

- Using the HIV self-test or understanding the results
- Getting additional HIV testing
- Getting HIV PrEP, condoms, or lubricant
- Contact your VA primary care provider or **call pharmacist [study PI and phone]**.

Contact Us

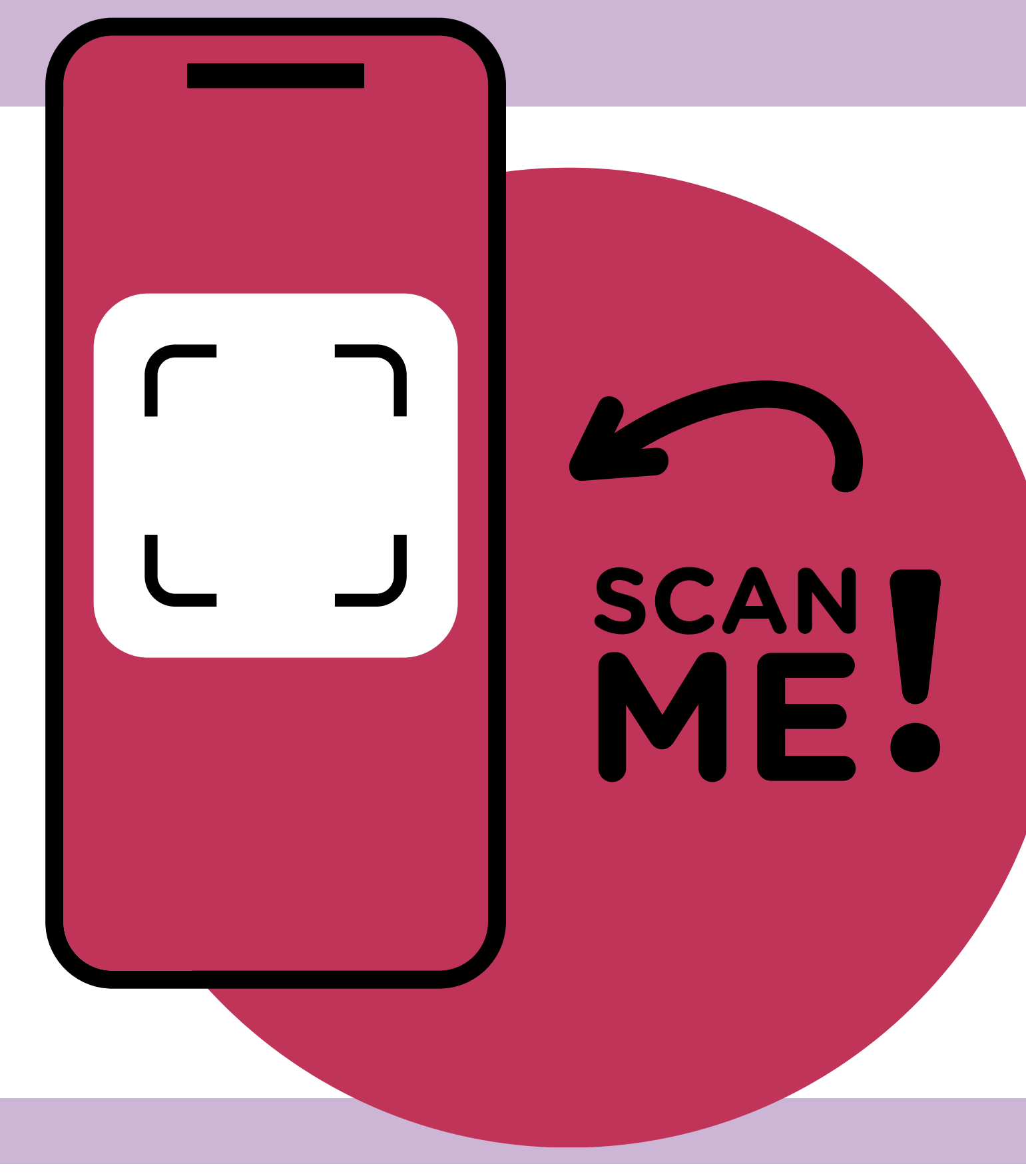

Need confidential crisis support?

- Dial 988 then press 1, or
- Text 838255
- Available 24/7 to support Veterans and their loved ones

Veterans Crisis Line
DIAL 988 then PRESS 1

Not a Veteran? Ineligible for VA care?

FREE resources are available in a community near you. These programs can connect you to HIV testing, prevention, and treatment services.

- **Alameda County:**
  - Berkeley Free Clinic (existing clients only): 510-548-2570
  - Honeycomb Hideout 510: 510-972-4483
  - HIV Education and Prevention Project of Alameda County: 510-434-0307
- **Humboldt County:**
  - North Coast AIDS Project Syringe Services: 707-268-2132
- **Lake County:**
  - Any Positive Change: 707-480-7319
- **Mendocino County**:
  - Mendocino County AIDS/Viral Hepatitis Network: 707-462-1932
- **San Francisco County:**
  - SF AIDS Foundation:
    - 470 Castro St: 415-581-1600
    - Mobile services in Bayview, Duboce Triangle, Mission District, and Tenderloin: 415-487-8043
  - San Francisco City Clinic: 628-317-6600
  - GLIDE: 415-674-5180
- **Santa Rosa County:**
  - Face to Face Sonoma County AIDS Network: 707-544-1581
- **Request HIV, viral hepatitis, and sexually transmitted infection self-test(s) to be mailed to your address:** https://takemehome.org

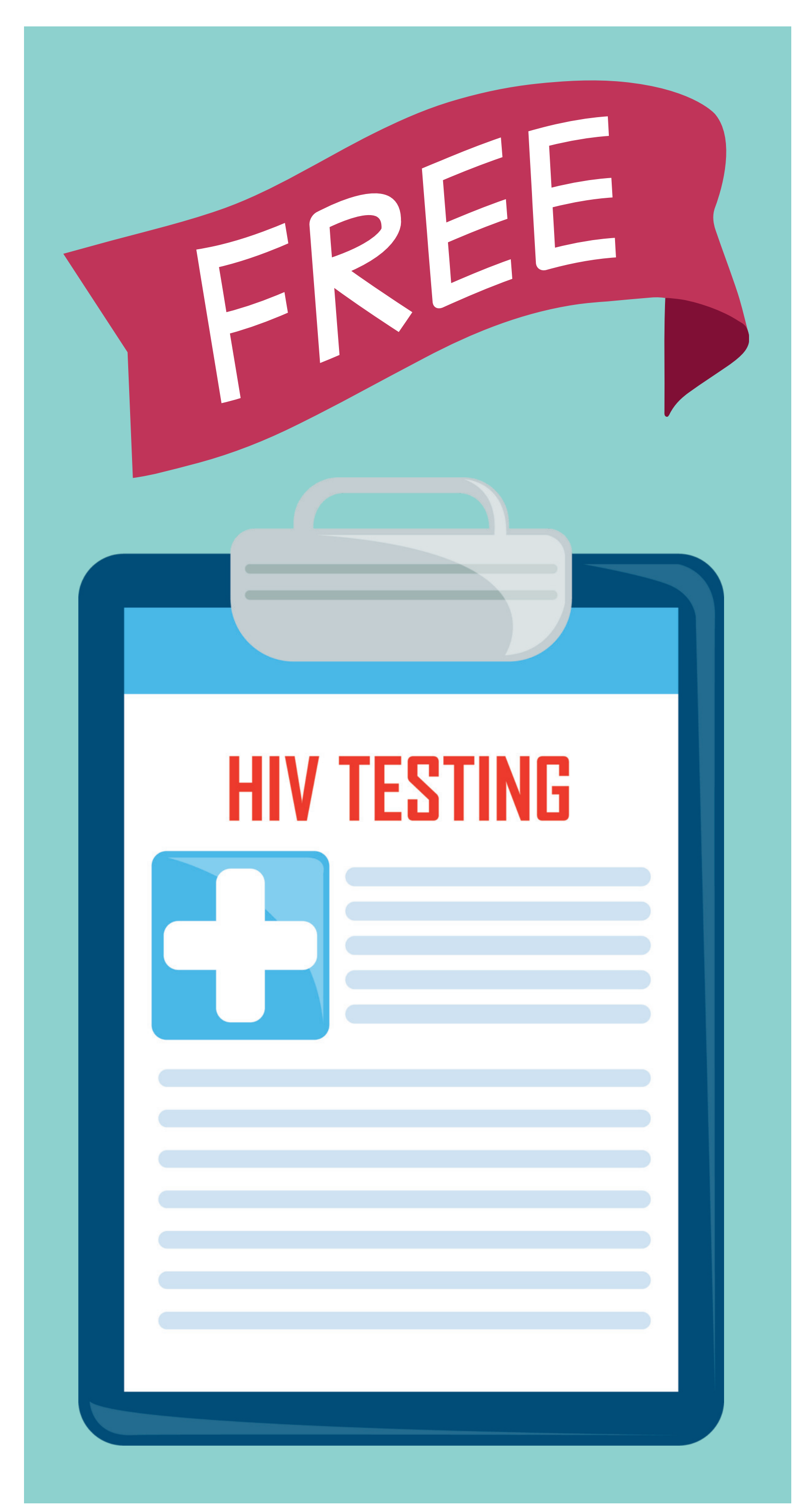

Supplement: Supplementary file 6 [file formative_v10i1e93868_app9.pdf]
