# Supplementary material for: Optimizing Veteran-Facing Materials for Vending Machine-Dispensed HIV Self-Testing: Formative Qualitative Study
Source: JMIR Form Res. 2026 Jul 23;10:e93868. doi: 10.2196/93868 (PMC13448198; doi:10.2196/93868)
Supplement: Supplementary file 9 [file formative_v10i1e93868_app11.docx]

**Qualitative Interview Guide/Script**

**Introduction:**

“Hello, my name is ___. I am a researcher/pharmacist/pharmacy student/___ from the University of California, San Francisco (UCSF), and the San Francisco VA. We are conducting a paid research study evaluating experiences and perspectives of Veterans who accessed and used a HIV self-test dispensed via harm reduction vending machines.”

**Interviewer:** *Obtain informed consent for interview and recording.*

“Before we proceed, I would like to review the informed consent with you. Your participation is voluntary, and your responses will be kept confidential. This interview will last for approximately 60 minutes. With your consent, we will do an interview that is audio-recorded, and you will complete a brief online demographics survey. Your name and identifying information will not be associated with your responses. Please review this consent form, and let me know if you have any questions, concerns, or would like a copy of the form.”

“Before we start, I want to remind you that there are no right or wrong answers—what matters most is your honest thoughts and experience. You can skip any question, take a break, or stop at any time. Are you ready to start?”

**Section A. Background and Prior HIV Testing**

“First, I’m going to ask you a few questions about HIV testing in general.”

1. “When you think about HIV testing, what comes to mind?”
   - Probe: words; phrases; images
2. “Tell me about your past HIV testing experiences?”
   - Probe: where (VA, public health, home); type (blood draw, fingerstick); what went well and what didn’t
3. “Before using the vending machine HIV self-test, what had you heard about HIV self-testing (if anything)?”
   - Probe: how you learned about it (advertisement on TV or in a store)

**Section B. Using the Vending Machine**

“Now I’m going to ask you about getting the HIV self-test from the vending machine.”

4. “Did you get an HIV self-test from the vending machine for yourself, someone else, or both?”
   - Probe: for a friend, family member, or another Veteran

*If someone else accessed the HIV self-test from the vending machine and gave it to the Veteran, skip to section C.*

5. “What led you to get an HIV self-test from the vending machine?”
   - Probe: convenient; private; free; curiosity; test yourself for HIV
6. “Walk me through what it was like accessing the HIV self-test from the vending machine.”
   - Probe: easy or challenging; anything confusing; what would improve the process
7. “What concerns did you have when accessing the HIV self-test from the vending machine?”
   - Probe: lack of privacy; fear of surveillance (e.g., video cameras, property management, security officer, other residents); stigma

**Section C. Peer Support**

“I’m going to ask you a few questions about helping others use one of the HIV self-tests. If it doesn't apply to you, just say so.”

8. “Have you helped anyone else use one of the HIV self-tests?”

*If yes:*

a) “Can you walk me through how you helped—What did helping look like?”

- Probe: explaining instructions; setting up materials; timing/reading results; emotional support; helping them decide on next steps

b) “What was that experience like for you?”
   - Probe: confidence; comfort level; worrying about saying/doing the right thing; concerns
   - Prompt: “What stood out most?” “How did it make you feel?” “What made you feel that way?”
c) “What resources would’ve made it easier to help someone else?”

- Probe: tip-sheet; video; hotline/real person to call; on-site training

9. “In your view, what role, if any, could Veterans play in helping other Veterans access or use HIV self-tests from the vending machine?”

- Probe: what would make peer support safe/helpful; what boundaries should be set; training/resource needs

**Section D. Using the HIV Self-Test**

“My next few questions are about your experience using the HIV self-test.”

10. “Tell me about your experience using the HIV self-test?”
    - Probe: setting/environment; what you used to guide you (instructions)
11. “Did anyone help you use the HIV self-test?

*If yes:*

a) “Walk me through what that was like for you.”
   - Probe: helpful/unhelpful; comfort; trust; privacy; feeling supported vs pressured
b) “What kind of help would you want or not want if you were going to do a self-test with someone else again?”
c) “How did having someone else involved affect how you felt before/during/after seeing the result?”
- Probe: anxiety; reassurance; embarrassment; motivation for follow-up

12. “What parts were easy, and what parts were hard?”
    - Prompt: What made it easy or difficult to use? How could it be made easier?
13. “What concerns, if any, came up while you were testing or interpreting the result?”
    - Probe: testing accuracy; understanding results; what to do next; worry/anxiety
14. “How did you feel when you saw your result?”
    - Prompt: It’s okay to skip this question.
15. “What support would have been most helpful before or after testing?”
    - Probe: real person to call; clear next steps; video instructions; crisis/mental health resources

**Section E. Follow-up Actions and Linkage**

16. “After you used the HIV self-test, what did you do next (if anything)?”
    - Probe: additional testing to confirm the results; contact a medical provider; talk to someone for support
17. “What would make it easier to get confirmatory testing or follow-up care, if needed?”
    - Probe: free; low-cost; easy scheduling; quick appointment; discreet locations; transportation; reminders

**Section F. Values and Preferences**

18. “How do you compare home testing to clinic-based testing? What do you prefer and why?”
19. “If the HIV self-test was not free, what would you be willing to pay (if anything)?”
    - Probe: $10, $20, $30

**Section G. Expansion**

20. “What other types of self-tests would you want available in the vending machine and why?”

“That concludes our interview. Is there anything else you would like to share with the research team at this time?

Thank you so much for your participation. As a reminder, your responses are confidential, and your insights are valuable to our research. If you have any questions, feel free to ask. Thank you again.”
