# Supplementary material for: Optimizing Veteran-Facing Materials for Vending Machine-Dispensed HIV Self-Testing: Formative Qualitative Study
Source: JMIR Form Res. 2026 Jul 23;10:e93868. doi: 10.2196/93868 (PMC13448198; doi:10.2196/93868)
Supplement: Supplementary file 10 [file formative_v10i1e93868_app6.docx]

Dear Veteran,

Thank you for your interest and willingness to participate in our focus group. We would like to hear your ideas and opinions about materials we plan to use in an upcoming paid research study at UCSF and San Francisco VA Medical Center. In this study, we aim to learn about Veteran interests, experiences, and perspectives accessing and using HIV self-tests from 15 harm reduction vending machines.

The focus group will consist of a moderator, moderator assistant, and 4 Veterans living with HIV. We will require approximately 5 hours of your time, including:

- 4 hours for review of materials
- 1 hour for the focus group discussion

In addition, you will have the opportunity to participate in written products, such as conference abstracts/presentations and a manuscript publication in a peer-reviewed journal. You will be compensated with a $1,000 honorarium for your time and expertise via mailed check.

To participate, please complete the following steps:

1. Complete the W9 form to receive your payment.
   a. Page 1:
      i. Section 1: Add your personal or business information.
      ii. Section 2: Add your email only. Do not change other fields.
      iii. Section 3: Check relevant boxes if completing as a business.
      iv. Section 4: Skip.
      v. Section 5: Add your name, title, date, and sign electronically.
   b. Page 2: Skip.
   c. Page 3: Skip.
   d. Email the completed W9 form to [research study team]. He will work with you directly if any changes are needed.
2. Before the focus group, review the attached research study materials.
   a. Recruitment fliers
   b. Education handouts
      i. HIV 101
      ii. Rapid Oral HIV Test Fact Sheet
      iii. TakeMeHome Handout
      iv. HIV testing and treatment resources by county: Alameda, Humboldt, Lake and Mendocino, San Francisco, and Sonoma Counties
   c. Qualtrics survey
      i. Direct link:
      ii. Or via QR Code:
      iii. Electronic/paper copy is attached for reference
   d. Qualitative interview guide
3. Document your feedback on the research study materials in the attached feedback form. Email the completed form **before the focus group** to [research study team].
4. Attend the 1-hour focus group scheduled **Thursday September 18^th^ at 5-6pm via Zoom**. Be prepared to discuss what you documented in the feedback form.

If you have any questions, concerns, or are unable to attend, please contact [research study team]. Otherwise, we look forward to meeting with you!

Sincerely,

[research study team]
