# Supplementary material for: Optimizing Veteran-Facing Materials for Vending Machine-Dispensed HIV Self-Testing: Formative Qualitative Study
Source: JMIR Form Res. 2026 Jul 23;10:e93868. doi: 10.2196/93868 (PMC13448198; doi:10.2196/93868)
Supplement: Supplementary file 11 [file formative_v10i1e93868_app7.docx]

**Appendix C. Reviewer Feedback From**

| **Product To Review** | **Questions for Reviewers** | **Reviewer Feedback**  **Instructions: Type or write your responses here.** |
| --- | --- | --- |
| **Recruitment Fliers** | - Do the fliers sufficiently answer these key questions about the research study?   - What is the study about?   - What do you want from me?   - Why should I care?   - Who is eligible to participate?   - Where/how do I get involved? |  |
|  | - Are the images/pictures on the fliers relevant? - Are the images/pictures on the fliers representative of:   - Diverse populations   - Veterans   - People living with HIV |  |

| **Product To Review** | **Questions for Reviewers** | **Reviewer Feedback**  **Instructions: Type or write your responses here.** |
| --- | --- | --- |
| **Recruitment Fliers** | - Are the fliers eye catching? Do they grab your attention? - Do the fliers have a good balance of images and white space? - Do you like the colors and fonts used on the fliers? |  |
|  | - Are the fliers easy to read? - Is the content on the fliers easy to understand? - Is the language on the fliers too technical? |  |

| **Product To Review** | **Questions for Reviewers** | **Reviewer Feedback**  **Instructions: Type or write your responses here.** |
| --- | --- | --- |
| **Recruitment Fliers** | - Are the fliers easy to act on? - Is having the QR code useful?   - Is the website useful? - Is there another/better way to share information about the study? |  |
|  | - What are some things we could do to improve the fliers? - How could we make the fliers more engaging |  |

| **Product To Review** | **Questions for Reviewers** | **Reviewer Feedback**  **Instructions: Type or write your responses here.** |
| --- | --- | --- |
| **Education Handouts** | - What were your overall impressions of the handouts? - What aspects of the handouts did you find more or less relevant? |  |
|  | - Were the handouts clear and easy to understand? - Were there any handouts that were confusing or unclear? |  |

| **Product To Review** | **Questions for Reviewers** | **Reviewer Feedback**  **Instructions: Type or write your responses here.** |
| --- | --- | --- |
| **Education Handouts** | - Were the handouts relevant? - Was the content in the handouts an appropriate level? Do we need to provide more educational information? - What handouts, if any, would you suggest we remove? - What types of handouts should we add?” |  |
|  | - What are some things we could do to improve the handouts? - How could we make the handouts more engaging? |  |

| **Product To Review** | **Questions for Reviewers** | **Reviewer Feedback**  **Instructions: Type or write your responses here.** |
| --- | --- | --- |
| **Qualtrics Survey** | - How would you describe your overall experience completing this survey? - What were your first impressions? - What aspects of the survey did you find more or less relevant? |  |
|  | - Were the questions clear and easy to understand? - Were there any questions that were confusing or unclear? - Was the survey flow and structure easy to follow? |  |

| **Product To Review** | **Questions for Reviewers** | **Reviewer Feedback**  **Instructions: Type or write your responses here.** |
| --- | --- | --- |
| **Qualtrics Survey** | - Were the survey questions relevant? - Were there any questions that were too intrusive?   - Was asking HIV self-test results intrusive? - What questions, if any, would you suggest we remove? - What questions, if any, would you suggest we change the wording? - What questions should we add? |  |
|  | - How long did it take you to complete the survey? - What type of device did you use to complete the survey? - Did you experience any technical issues? - How easy or difficult was the survey to complete? - What could be done to make the survey easier and faster to complete? |  |

| **Product To Review** | **Questions for Reviewers** | **Reviewer Feedback**  **Instructions: Type or write your responses here.** |
| --- | --- | --- |
| **Qualtrics Survey** | - What are some things we could do to improve the survey? - How could we make the survey more engaging? - How could we make the survey more user-friendly? |  |

| **Product To Review** | **Questions for Reviewers** | **Reviewer Feedback**  **Instructions: Type or write your responses here.** |
| --- | --- | --- |
| **Interview Guide** | - What were your overall impressions of the interview questions? - What aspects of the interview questions did you find more or less relevant? |  |
|  | - “Were the questions clear and easy to understand? - Were there any questions that were confusing or unclear? - Do the interview questions flow well? |  |
| **Product To Review** | **Questions for Reviewers** | **Reviewer Feedback**  **Instructions: Type or write your responses here.** |
| **Interview Guide** | - Were the interview questions relevant? - Were there any questions that were too intrusive? - What questions, if any, would you suggest we remove? - What questions, if any, would you suggest we change the wording? - What questions should we add?” |  |
|  | - What are some things we could do to improve the interview questions? - How could we make the interview more engaging? |  |
